# Cannabis use associated with lower mortality among hospitalized Covid-19 patients using the national inpatient sample: an epidemiological study

**DOI:** 10.1186/s42238-024-00228-w

**Published:** 2024-04-06

**Authors:** Joseph-Kevin Igwe, Ugo Alaribe

**Affiliations:** 1 Department of Medicine, Baylor College of Medicine, 1 Baylor Plaza, Houston, TX 77030 USA; 2 Caribbean Medical University School of Medicine, 5600 N River Rd Suite 800, Rosemont, IL 60018 USA

**Keywords:** Covid-19, Cannabis, Acute respiratory distress syndrome (ARDS), Mortality, Acute pulmonary embolism, Inpatient outcomes, Epidemiology

## Abstract

**Background:**

Prior reports indicate that modulation of the endocannabinoid system (ECS) may have a protective benefit for Covid-19 patients. However, associations between cannabis use (CU) or CU not in remission (active cannabis use (ACU)), and Covid-19-related outcomes among hospitalized patients is unknown.

**Methods:**

In this multicenter retrospective observational cohort analysis of adults (≥ 18 years-old) identified from 2020 National Inpatient Sample database, we utilize multivariable regression analyses and propensity score matching analysis (PSM) to analyze trends and outcomes among Covid-19-related hospitalizations with CU and without CU (N-CU) for primary outcome of interest: Covid-19-related mortality; and secondary outcomes: Covid-19-related hospitalization, mechanical ventilation (MV), and acute pulmonary embolism (PE) compared to all-cause admissions; for CU vs N-CU; and for ACU vs N-ACU.

**Results:**

There were 1,698,560 Covid-19-related hospitalizations which were associated with higher mortality (13.44% vs 2.53%, *p* ≤ 0.001) and worse secondary outcomes generally. Among all-cause hospitalizations, 1.56% of CU and 6.29% of N-CU were hospitalized with Covid-19 (*p* ≤ 0.001). ACU was associated with lower odds of MV, PE, and death among the Covid-19 population. On PSM, ACU(N(unweighted) = 2,382) was associated with 83.97% lower odds of death compared to others(N(unweighted) = 282,085) (2.77% vs 3.95%, respectively; aOR:0.16, [0.10–0.25], *p* ≤ 0.001).

**Conclusions:**

These findings suggest that the ECS may represent a viable target for modulation of Covid-19. Additional studies are needed to further explore these findings.

**Supplementary Information:**

The online version contains supplementary material available at 10.1186/s42238-024-00228-w.

## Key points

*Question:* Is cannabis use associated with adverse Covid-19-related outcomes among hospitalized patients?

*Findings:* History of cannabis use was associated with significantly lower odds of Covid-19-related hospitalization, mechanical ventilation, and death compared to those without history of cannabis use.

*Meaning:* Our study findings suggest that the endocannabinoid system may represent a viable target for modulation of moderate and severe Covid-19 disease (Covid-19). Additional prospective studies are needed to further explore these findings.

**Supplementary Information:**

The online version contains supplementary material available at 10.1186/s42238-024-00228-w.

## Introduction

Cannabis has become increasingly legalized for recreational and medicinal use. As of November 8, 2023, 24 U.S. states (and the District of Columbia) have enacted measures to regulate non-medical adult cannabis use, and 38 states permit the use of medical cannabis (State Medical Marijuana Laws (National Conference of State Legislatures website) 2023) According to the CDC (Centers for Disease Control), 48 million people used cannabis at least once in 2019 (Centers for Disease Control and Prevention 2021). It is also estimated that 2 million US adults who have cardiovascular disease have used cannabis at least once (DeFilippis et al. 2020).

Amongst the many cannabinoids biosynthesized by the cannabis sativa plant, two of the most studied and more abundant are Δ9-tetrahydrocannabinol (THC/ Δ9-THC/dronabinol), the principal psychoactive component of cannabis, and cannabidiol (CBD) (Chayasirisobhon 2020). THC functions as a cannabinoid receptor 1 (CB1) and cannabinoid receptor 2 (CB2) partial agonist, while CBD is a non-competitive negative allosteric modulator of CB1 and CB2, with reports indicating that it potently modulates cannabinoid receptor responses to THC and endogenous cannabinoid 2‐arachidonylglycerol (Chayasirisobhon 2020; Laprairie et al. 2015; Pertwee et al. 2002; Pertwee 2008). Cannabinoid receptors 1 and 2 (CB_1_ and CB_2_) are distributed in multiple tissue beds and cell types (Cosentino et al. 2022). Cannabinoid effects related to activation of the CB_1_ receptor shift serum milieu towards sympathetic activation, cytokine production, and increased interleukin (IL) production (DeFilippis et al. 2020; Skipina et al. 2022; Auer et al. 2018; Wei et al. 2022). On the other hand, its function at CB_2_ receptors, localized primarily in immune cells – the tonsils, spleen, peripheral blood mononuclear cells (PBMC), microglia, and thymus—and function as an inhibitor of adenylate cyclase highlight its role in regulation of inflammation. Additionally, multiple studies have confirmed the presence of CB_2_ mRNA and protein activation components across different human lymphocytes (Pertwee 2008; Cosentino et al. 2022; Miller and Stella 2008; Rieder et al. 2010; Huang et al. 2020; Rossi et al. 2020; Wang et al. 2020a; Howlett et al. 2002).

The plasma half-life of THC is approximately 1–3 days in those with occasional cannabis use and 5–13 days with chronic, regular cannabis use; and CBD half-life is 18–32 h. However, both CBD and THC are highly lipophilic with a long elimination half-life within adipocytes, and under normal conditions, they passively diffuse from fat back into blood (Chayasirisobhon 2020; Ellis Jr et al. 1985; Gunasekaran et al. 2009). Prior reports indicate that THC can be observed in fat cells biopsy up to 28 days after last use (Johansson et al. 1989), and the presence of THC has been reported in the urine among chronic, heavy user of cannabis (a majority (60.5%) of whom reported ≥ 1 time daily cannabis use with a history of marijuana use 2–21 years) after 77 days, despite extended periods of abstinence (Ellis Jr et al. 1985). Notably, the accumulation, storage, and release of THC and its metabolites in adipose tissue, and its serum concentrations over time and in specific settings are not stagnant. Gunasekaran et al. demonstrated that sympathetic activation of catecholamine-induced lipolysis may enhance the transient shifts in the serum THC concentrations, increasing over time in relation to extent and duration of adrenocorticotropic hormone (ACTH) stimulation or food deprivation (Gunasekaran et al. 2009). As such, the functional activity of THC may not be limited to its acute effects, and its functional components stored in adipocytes and released into the serum may function to modulate the endocannabinoid system (ECS) long after last use. In this respect, the long-term storage and release of THC may play a role in mitigating severe acute respiratory syndrome coronavirus 2 (SARS-CoV-2) viral infection as well as the associated outcomes of disease among those patients hospitalized with Covid-19.

The recent study by Nguyen et al. analyzing molecular and cellular changes associated with CBD and SARS-CoV-2 suggests that CBD acts early in the infection cycle, in a post-entry step, effectively inhibiting viral RNA expression and reversing viral-induced changes in host gene expression. Notably, these effects were noted for the original SARS-CoV-2 variant and other variants (alpha, Beta, and gamma), with significantly more potent antiviral response for CBD compared to structurally closely related congeners THC, cannabidiolic acid (CBDA), cannabidivarin (CBDV), cannabichromene (CBC), or cannabigerol (CBG). In the study, they demonstrated a 60% and 99% reduction in induction of viral genes responsible for spike, envelope, and nucleocapsid proteins associated with inactive CBD homologs and cannabidiol, respectively; and a significant negative association with SARS-CoV-2–positive test results among patients with active CBD use (Epidiolex (cannabidiol) 100 mg/ml oral solution) among matched groups of patients using the National COVID Cohort Collaborative dataset (Nguyen et al. 2002). Their findings demonstrated that CBD use, or ECS modulation may play a role in modulation of effects related to Covid-19.

### Study objective

Prior studies have been subject to limited sample size and limited control of confounding factors related to cannabis use; and others have elucidated associated risk factors for severe Covid-19, noting obesity, chronic kidney disease, and diabetes as chief co-morbid conditions with increased risk for more severe Covid-19 (Abe et al. 2021; Lopez et al. 2021; Hirsch et al. 2020; Shover et al. 2022; Cheng et al. 2020; Rubin et al. 2020; Costela-Ruiz et al. 2020; Briand-Mésange et al. 2020; He et al. 2021; Govender et al. 2021; Chen et al. 2021, 2023; Al-Hakeim et al. 2023; Chandrashekhar Joshi and Pozzilli 2022; Nunn et al. 2022). What is more, the increased risk and strong associations between new-onset diabetes and insulin resistance during acute-phase Covid-19 disease and chronically among those with long-term symptoms of Covid-19 implicate metabolic dysregulation as a key component in the pathophysiological mechanism of Covid-19 (Abe et al. 2021; Lopez et al. 2021; Hirsch et al. 2020; Shover et al. 2022; Cheng et al. 2020; Rubin et al. 2020; Costela-Ruiz et al. 2020; Briand-Mésange et al. 2020; He et al. 2021; Govender et al. 2021; Chen et al. 2021, 2023; Al-Hakeim et al. 2023; Chandrashekhar Joshi and Pozzilli 2022; Nunn et al. 2022). Additionally, vascular injury and coagulopathy associated with severe disease have been implicated in multi-organ system dysfunction, with reports indicating an associated benefit for those receiving anticoagulation therapy during inpatient admission (Levi et al. 2020; Farkouh et al. 2022; Rentsch et al. 2021). As such, we hypothesized that the functional consequences of ECS modulation associated with chronic or repeated cannabis use may dampen inflammatory responses associated with Covid-19 and result in better outcomes generally among Covid-19 hospitalizations, and among those receiving antiviral therapy when controlling for long-term antiplatelet and anticoagulation therapies during inpatient admission (Food and Drug Administration 2020; Badgujar et al. 2020; Humeniuk et al. 2020; Beigel et al. 2020; de Wit et al. 2020; Fact sheet for healthcare providers: Emergency Use Authorization (EUA) of baricitinib n.d.; CDC 2022). Using the National Inpatient Sample (NIS) database (HCUP National Inpatient Sample (NIS) 2020), we performed an epidemiologic, analytic study with a retrospective cohort study design among inpatient, hospitalization encounters during the 2020 Covid-19 Pandemic and analyzed the incidence, predictors, and outcomes of Covid-19 disease among those with vs without history of cannabis use.

## Methods

### Data source and study population

We queried the NIS database from January to December 2020 to identify all patients aged ≥ 18 years presenting with Covid-19. The NIS database is the largest publicly available all-payer inpatient healthcare database designed to produce U.S. (United States) regional and national estimates of inpatient utilization, access, charges, quality, and outcomes. The 2020 NIS dataset represents a stratified systematic random sample of 20% of U.S. community hospital discharges which is constructed of a 20% national patient-level sample, with non-representative sampling across hospitals. The complex sampling design of the NIS is further described in eAppendix 2: Data Source.

### Definitions

Covid-19 disease was identified using International Classification of Diseases, Tenth Revision, Clinical Modification (ICD-10-CM) diagnosis code of U07.1 (Covid-19, virus identified) and B97.29 (Coronavirus infection) for the months January to December in any position on a medical claim, inclusive of inpatient hospital encounters. *Cannabis use (CU)* was defined as *cannabis abuse* or *cannabis dependence*. These diagnoses were identified using ICD-10-CM diagnosis code corresponding to *cannabis abuse* or *cannabis dependence* in any position on a medical claim, inclusive of inpatient hospital encounters and can be found in eAppendix 1. C*annabis abuse* was further stratified into *cannabis abuse not in remission (ACU-Abs)* and *cannabis abuse in remission*; and *cannabis dependence* was stratified into *cannabis dependence not in remission (ACU-Dep)* and *cannabis dependence in remission*. For the purposes of this article, *active cannabis use (ACU)* was defined as *ACU-Abs* or *ACU-Dep* and excluded *cannabis abuse in remission* and *cannabis dependence in remission*. Additional information on the ICD-10-CM codes utilized to define the cannabis use groups and other definitions can be found in the Supplements I and II.

Moreover, the All-Patient Refined Diagnosis Related Groups (DRGs) (APR-DRG) was used to further characterize Covid-19-related encounters. The All Patient Refined DRGs incorporate severity of illness subclasses into the AP-DRGs and consider the severity of illness, risk of mortality, and resource intensity. The APDRG Severity of Illness Subclass (APDRG-S) specifically represents the extent of physiologic decompensation or organ system loss of function, and it has been noted in prior studies to model risk of mortality (HCUP National Inpatient Sample (NIS) 2020; Averill et al. n.d.; Jamal et al. 2020; McCormick et al. 2018).

The ICD-10-CM diagnostic criteria for cannabis dependence have been defined broadly to include difficulty with regulation of use or continued substance use associated with impaired ability to control use with increased priority to use over other activities and persistence of use despite harm and adverse outcomes associated with use; and often associated with tolerance and withdrawal. Empirical studies published recently show that the DSM-5 criteria for alcohol and cannabis use disorders captured different individuals and a higher prevalence compared to ICD 10, and draft ICD 11 diagnoses. Moreover, epidemiological data indicate that the majority of those who use cannabis do not have problems related to their use, but a substantial subset (10–30%) do report experiencing symptoms and consequences consistent with a cannabis use disorder (CUD) (Budney et al. 2019). However, these classification systems and the methodological and diagnostic criteria which contextualize CUD and cannabis dependence have recently come under increased scrutiny, and defining terms for length of use such as occasional and chronic use has not been assessed using the NIS. Additionally, the applicability of these terms among those receiving medically prescribed cannabis remains in question, and we do not believe cannabinoid- derived medical products such as Epidiolex were captured in the ICD-10-CM code for cannabis use. The NIS dataset is not able to differentiate those with medical prescription for cannabis vs others, although it is worth mentioning the significant difference in trends of cannabis dependence discharges between hospital regions and divisions (Supplement Figs. 4–8), noting that the prevalence of ACU-Dep among all *cannabis use*-related hospitalizations was significantly different in NIS divisions with states with no legalization or only medical legalization such as West South Central (Oklahoma, Texas, Arkansas, Louisiana) compared to those with medical and recreational legalization such as the Pacific Region (Alaska, Washington, Oregon, California, Hawaii) (7.26% vs 8.74%, *p* = 0.007) (Supplement Table 16). As such, the differences in trend and characteristics defining *cannabis use* between regions during the Covid-19 Pandemic may be subject to contextual, regional, and patient-related factors and motivations outside of the bounds of this study (Saunders 2017).

### Statistical analysis

Chi-square analysis, ANOVA (Analysis of variance), multivariable regression analysis (MRA), and propensity score matched analysis (PSM) were conducted to analyze significant associations between baseline characteristics, comorbid conditions, population median Elixhauser-index sum score (ELIX), and medication use; and for primary outcome: Covid-19-related inpatient mortality; and secondary outcomes: Covid-19-related hospitalization among all-cause admissions; mechanical ventilation (MV); and acute pulmonary embolism (PE). Part I of the manuscript characterizes Covid-19-related hospitalization and outcomes compared to other hospitalizations within the cohort and compares primary outcome to all-cause- and pneumonia-*related-* inpatient mortality, procedural MV, and PE. Part II of the manuscript focuses on primary outcome: Covid-19-related inpatient mortality; and secondary outcomes: Covid-19-related hospitalization among all-cause admissions; procedural MV; and acute PE for those with history of CU versus those *without history of cannabis use (N-CU)*. The Elixhauser Comorbidity Index is a method of categorizing comorbidities of patients based on the International Classification of Diseases (ICD) diagnosis codes found in administrative data and includes 31 comorbid conditions. It has been shown to effectively model 30-day, in-hospital, and 1-year mortality in older adults in prior studies (Menendez et al. 2014; Potts et al. 2019; Monterde et al. 2021; Mehta et al. 2022; Kim et al. 2018) STATA/MP software was used for all analyses (StataCorp. 2021. Stata Statistical Software: Release 17. College Station, TX: StataCorp LLC). Additional information on statistical analysis and other associated definitions can be found in Supplement II.

### Propensity score matched analysis

To account for confounding factors related to hospital encounter and hospitalization-associated outcomes, PSM on the unweighted sample observations was utilized for primary and secondary outcomes of interest. We used a 0.1 caliper width to account for age, race, sex, admission month, hospital characteristics: hospital region, hospital bed size, location/teaching status of hospital; primary insurance payor: Medicare, Medicaid, Private, Self-Pay, Other, No Charge; median household income for patient’s ZIP code, ELIX, medications, comorbid conditions, *admission month*, and MV status. Double robust results following PSM were achieved by multivariable logistic regression on the matched sample and used the frequency weights derived from the matching procedure. The multivariable logistic regression used the same matching covariates except for secondary outcome: *acute pulmonary embolism* as described in the Supplement. Additional information on the propensity score matched analysis and regression tables can be found in Supplement II.

## Results

### Covid-19 associated with worse outcomes compared to others

Amongst 27,667,386 all-cause admissions meeting inclusion criteria, we identified 1,698,560 (6.14%) Covid-19-related hospitalizations. Among this Covid-19 population, the mean age 63.20 ± 0.09 years; 885,110 (52.11%) were male. There were 25,968,826 (93.86%) patients with no history of Covid-19-related hospitalization (N-COVID) with a mean age 57.63 ± 0.10 years; 11,242,670 (43.29%) were male (Supplement Table 1). The ELIX score of the population meeting inclusion criteria was 3 [IQR:1–5], while the ELIX score of the Covid-19 subpopulation was 4 [IQR:2–5]. Mean Elixhauser sum score for Covid-19 patient encounters was significantly greater than that of others in the General Population (mean ± SD = 3.70 ± 0.01 vs 3.44 ± 0.01, *p* ≤ 0.001, respectively).

The Covid-19 population mean mortality rate (13.44%) was significantly higher than N-COVID (2.53%) and accounted for 25.79% of all hospital deaths during the index period (*p* ≤ 0.001) and 39.15% of all hospital deaths in December 2020 (*p* ≤ 0.001), with a mean age of death 72.75 ± 0.10. There was a significant negative trend in mortality from March 2020 (21.60%) to December 2020 (11.56%) (Supplement Figs. 1 and 2). The mean difference in the incidence of and mortality associated with MV (8.36 pp and 6.67 pp, respectively) or acute PE (Pulmonary Embolism) (1.14 pp and 51.31 pp, respectively) were both significantly higher than that of the N-Covid-19 population (*p* ≤ 0.001 for each). The APR-DRG Severity Score of three or more was associated with 99.8% of those Covid-19 patients receiving MV (*p* ≤ 0.001).

On PSM of 339,719 Covid-19 observations with 1) matched controls among all-cause admissions 2) matched controls within the pneumonia subpopulation, Covid-19 was associated with significantly higher mortality (aOR, [CI (Confidence Interval) Lower Bound, Upper Bound], *p*-value: aOR: 4.66, [4.56, 4.76], *p* ≤ 0.001; aOR: 4.78 [4.61, 4.96], *p* ≤ 0.001); acute PE (aOR:1.92, [1.84, 2.00], *p* ≤ 0.001; aOR: 3.57, [3.23, 3.95], *p* ≤ 0.001); MV (aOR: 2.52, [2.48, 2.57], *p* ≤ 0.001; aOR: 2.20, [2.11, 2.29], *p* ≤ 0.001); compared to all-cause admissions and non-Covid-19 pneumonia, respectively (Supplement Table 3).

### Cannabis use among Covid-19 patients associated with better outcomes compared to others

Among the 27,667,386 all-cause hospitalizations, 1.56% of those with history of cannabis use and 6.29% of those without cannabis use were hospitalized with Covid-19 (*p* ≤ 0.001). Specifically, among the 1,698,560 Covid-19-related hospitalizations, there were 13,750 (0.81%) CU patients with a mean age of 41.46 ± 0.33 years and 9,060 (65.89%) were male; and 1,684,810 (99.19%) N-CU patients with a mean age of 63.02 ± 0.09 years and 876,050 (52.00%) were male (Tables 1 and 2). Among Covid-19 hospitalizations, inpatient mortality for CU (2.87%) was significantly lower than N-CU (13.52%; *p* ≤ 0.001); and lower among MV CU (*N* = 920) compared to MV N-CU (*N* = 203,640) (33.15% vs 58.67%, *p* ≤ 0.001). The mortality rate for those in *AGE Group 4*:65–79 was significantly lower for CU (*N* = 1,135) compared to N-CU (*N* = 540,185) (9.69% vs 17.17%, *p* = 0.003); for *AGE Group 3*:55–64 for CU (*N* = 2,105) compared to N-CU (324,735) (5.46% vs 10.48%, *p* ≤ 0.001); and for *AGE Group 2*:30–54 for CU (*N* = 5,960) compared to N-CU (404,740) (1.59% vs 4.85%, *p* ≤ 0.001). Moreover, CU mean length of stay (LOS) and total charge (TOTCHG) (7.17 ± 0.30 days; $73,171.75 ± 3,484.18) were significantly lower than that of N-CU patients (7.98 ± 0.03 days; $91,168.11 ± 1,212.67) (*p* ≤ 0.001 for both) (See Supplement II for additional demographic information).

**Table 1:** Baseline demographics of Covid-19 hospitalizations

	**CU**	**No CU**
**Frequency (N)**	**13,750**	**1,684,810**
	**Mean (Std err.)**	**Mean (Std err.)**
**Age in years at admission**	41.46 (0.32)**	63.37 (0.09)**
**Length of Stay**	7.17 (0.30)**	7.98 (0.03)**
**Total Charges**	$73,171.75 (3415.38)**	$91,168.11 (1188.73)**
**Age Group**	**Mean Percent (Standard Error)**	**Mean Percent (Standard Error)**
**AGE Group 1: 18–29**	30.65 (0.92)**	4.73 (0.07)**
**AGE Group 2; 30–54**	44.29 (0.95)**	24.03 (0.16)**
**AGE Group 3: 55–64**	15.71 (0.69)**	19.28 (0.08)**
**AGE Group 4: 65–79**	8.61 (0.51)**	32.08 (0.13)**
**AGE Group 5: 80–85**	0.58 (0.16)**	8.52 (0.07)**
**AGE Group 6: 86–120**	0.15 (0.07)**	11.36 (0.10)**
**Racial or Ethnic Group**		
**White**	38.95% (1.10)**	50.85% (0.56)**
**Black**	35.96% (1.10)**	19.11% (0.40)**
**Hispanic**	16.95% (0.90)**	21.42% (0.50)**
**Asian/Pacific Islander**	1.42% (4.52)**	3.30% (0.12)**
**Native American**	2.76% (0.41)**	1.00% (0.09)**
**Other Racial or Ethnic Group**	3.96 (0.42)	4.33 (0.19)
**Sex**		
**Male**	65.91%**	52.00%**
**Female**	34.09% (0.90)**	48.00% (0.12)**
**Primary Expected Payer**		
**Medicare**	19.38% (0.75)**	50.84% (0.27)**
**Medicaid**	43.14% (1.09)**	14.27% (0.24)**
**Private Insurance**	22.41% (0.86)**	26.52% (0.25)**
**Self-pay**	8.83% (0.59)**	3.74% (0.13)**
**No charge**	0.54% (0.15)**	0.27% (0.05)**
**Other**	5.69% (0.54)**	4.35% (0.12)**
**Length of Stay Group**		
**Less than 7 Days**	68.73 (1.08)**	60.32 (0.17)**
**7–13 Days**	19.71 (0.79)**	25.54 (0.11)**
**14–20 Days**	5.85 (0.49)**	8.02 (0.06)**
**21–29 Days**	3.13 (0.32)	3.99 (0.04)**
**30–45 Days**	1.27 (0.23)**	2.15 (0.03)**
**45–60 Days**	0.62 (0.14)	0.61 (0.02)
**61–780 Days**	0.69 (0.17)**	0.36 (0.01)**
**Total Charge in US Dollars**		
**Less than $50 k**	62.03% (1.12)**	54.85% (0.40)**
**$50,000–99,999**	21.77% (0.87)**	23.52% (0.19)**
**$100 k–249,999**	12.53% (0.64)**	15.19% (0.19)**
**$250 k–499,999**	2.55% (0.31)**	4.67% (0.08)**
**$500 k–1 m**	1.11% (0.21)**	1.77% (0.05)**
**ZIPINC_QRTL—Median Income for Patient Zip Code**		
**ZIPINC_QRTL 1- $1–24,999**	42.73% (1.26)**	33.88% (0.56)**
**ZIPINC_QRTL 2- $25,000–34,999**	25.08% (0.98)**	27.13% (0.38)**
**ZIPINC_QRTL 3- $35,000–44,999**	18.85% (0.87)**	22.22% (0.35)**
**ZIPINC_QRTL 4- $45,000 or more**	13.35% (0.79)**	16.78% (0.47)**
**Hospital Region**		
**HOSP_REGION 1: Northeast**	17.38% (1.14)**	19.02% (0.53)**
**HOSP_REGION 2: Midwest**	24.25% (1.18)**	22.18% (0.46)**
**HOSP_REGION 3: South**	34.51% (1.30)**	40.77% (0.57)**
**HOSP_REGION 4: West**	23.85% (1.35)**	18.04% (0.45)**
**Bed-size of Hospital**		
**HOSP_BEDSIZE 1: Small**	20.95% (1.15)**	24.30% (0.52)**
**HOSP_BEDSIZE 2: Medium**	27.13% (1.19)**	29.03% (0.50)**
**HOSP_BEDSIZE 3: Large**	51.93% (1.42)**	46.67% (0.59)
**Location & Teaching Status of Hospital**		
**HOSP_LOCTEACH 1: Rural**	4.04% (0.42)**	9.71% (0.24)**
**HOSP_LOCTEACH 2: Urban Nonteaching**	14.44% (0.88)**	18.54% (0.40)**
**HOSP_LOCTEACH 3: Urban Teaching**	81.53% (0.96)**	71.76% (0.46)**
**Admission on the Weekend**	26.58% (0.84)	25.89% (0.08)
**Admission Month**		
**January**	0.51% (0.14)**	0.21% (0.01)**
**February**	0.55% (0.14)**	0.16% (0.009)**
**March**	3.01% (0.35)**	4.72% (0.16)**
**April**	7.41% (0.49)**	11.35% (0.26)**
**May**	6.75% (0.53)	6.34% (0.11)
**June**	8.10% (0.55)**	6.03% (0.10)**
**July**	12.72% (0.63)**	11.12% (0.17)**
**August**	9.72% (0.59)**	7.55% (0.09)**
**September**	6.75% (0.46)**	5.52% (0.06)**
**October**	8.62% (0.53)	8.97% (0.10)
**November**	16.65% (0.72)**	18.24% (0.16)**
**December**	19.22% (0.78)	19.79% (0.17)

*ZIPQRTL* Median household income for patient's ZIP Code (based on current year)

^**^P less than or equal to 0.05 on χ2 analysis

**Table 2:** Elixhauser comorbidities and other comorbid conditions and medication use among Covid-19 -related hospitalizations

	**CU** ***N* = 13,750**	**No CU** ***N* = 1,684,810**
	**Mean Percent (Std. err.)**	**Mean Percent (Std. err.)**
**Congestive Heart Failure**	11.02% (0.59)**	17.61% (0.13)**
**Cardiac Arrhythmias**	13.93% (0.70)**	24.69% (0.14)**
**Valvular Disease**	2.25% (0.28)**	3.99% (0.05)**
**Pulmonary Circulation Disorders**	3.78% (0.66)**	5.27% (1.28)**
**Peripheral Vascular Disorders**	2.87% (1.14)**	4.79% (0.48)**
**Paralysis**	1.78% (0.25)**	1.33% (0.02)**
**Other Neurological Disorders**	13.93% (2.96)**	17.15% (4.19)**
**Uncomplicated Hypertension**	37.05% (8.55)**	55.14% (13.65)**
**Complicated Hypertension**	14.69% (0.71)**	27.10% (0.16)**
**Chronic Pulmonary Disease**	22.33% (0.83)	22.11% (0.14)
**Uncomplicated Diabetes**	8.87% (0.55)**	14.80% (0.10)**
**Complicated Diabetes**	15.24% (0.70)**	26.27% (0.14)**
**Hypothyroidism**	4.07% (0.37)**	13.23% (0.10)**
**Renal Failure**	11.09% (0.61)**	21.05% (0.13)**
**Liver Disease**	8.51% (0.53)**	5.42% (0.06)**
**Peptic Ulcer Disease without Bleeding**	0.62% (0.15)**	0.31% (0.01)**
**AIDS/HIV**	1.31% (0.26)**	0.26% (0.01)**
**Lymphoma**	0.55% (0.14)	0.81% (0.02)
**Metastatic Cancer**	0.87% (0.18)	1.12% (0.02)
**Solid Tumor Without Metastasis**	1.75% (0.25)**	2.48% (0.60)**
**Rheumatoid Arthritis/Collagen Vascular disorders**	1.67% (0.23)**	2.94% (0.04)**
**Coagulopathy**	8.62% (0.55)**	12.14% (0.18)**
**Obesity**	20.51% (0.81)**	25.56 (0.22)**
**Severe Obesity**	10.98% (0.63)**	13.53% (0.11)**
**Obesity with Complicated Diabetes**	2.27% (0.05)**	5.61% (0.05)**
**Weight Loss**	5.31% (0.46)**	7.85% (0.13)**
**Fluid & Electrolyte Disorders**	40.11% (9.27)**	50.36% (12.43)**
**Blood Loss Anemia**	0.29% (0.10)**	0.45% (0.01)**
**Deficiency Anemia**	4.04% (0.40)	3.86% (0.05)
**Alcohol Abuse**	18.22% (0.77)**	2.53% (0.04)**
**Drug Abuse**	--	1.79% (0.04)
**Psychoses**	14.65% (0.87)**	2.47% (0.05)**
**Depression**	20.40% (0.82)**	11.62% (0.12)**
**Remdesivir Administration**	9.13% (0.58)**	22.91% (0.30)**
**Baricitinib Administration**	0.001 (8.03e-06)**	0.01 (0.002)**
**Long-term Steroid Use**	1.24% (0.21)**	1.74% (0.04)**
**Long-term Anticoagulation**	4.55% (0.40)**	9.56% (0.10)**
**Long-term (Current) Drug Therapy**	18.07% (0.88)	19.70% (0.46)
**Long-Term NSAID**	1.16% (0.21)	0.94% (0.03)
**Long-Term Aspirin Use**	7.49% (0.51)**	14.45% (0.19)**
**History of Bariatric Surgery**	0.52% (0.14)	0.73% (0.02)
**Septic Shock**	3.05% (0.33)**	7.33% (0.09)**
**Cardiogenic Shock**	0.62% (0.15)	0.63% (0.02)
**Active Cannabis Abuse Disorder**	92.07% (0.38)	--
**Active Cannabis Dependence Disorder**	5.85% (0.37)	--
**Cannabis Abuse Remission**	1.71% (0.26)	--
**Cannabis Dependence Remission**	0.36% (0.12)	--
**APDRG 3&4**	85.42% (0.72)**	94.29% (0.08)**
**Elixhauser Sum ≥ 4**	51.56% (1.05)	50.35% (0.23)
**Covid-19 Primary Diagnosis**	36.64% (1.02)**	60.37% (0.31)**
**Covid-19 Diagnosis after Admission**	63.36% (1.02)**	39.63% (0.31)**

^**^P less than or equal to 0.05 on χ2 analysis

**Fig. 1:**
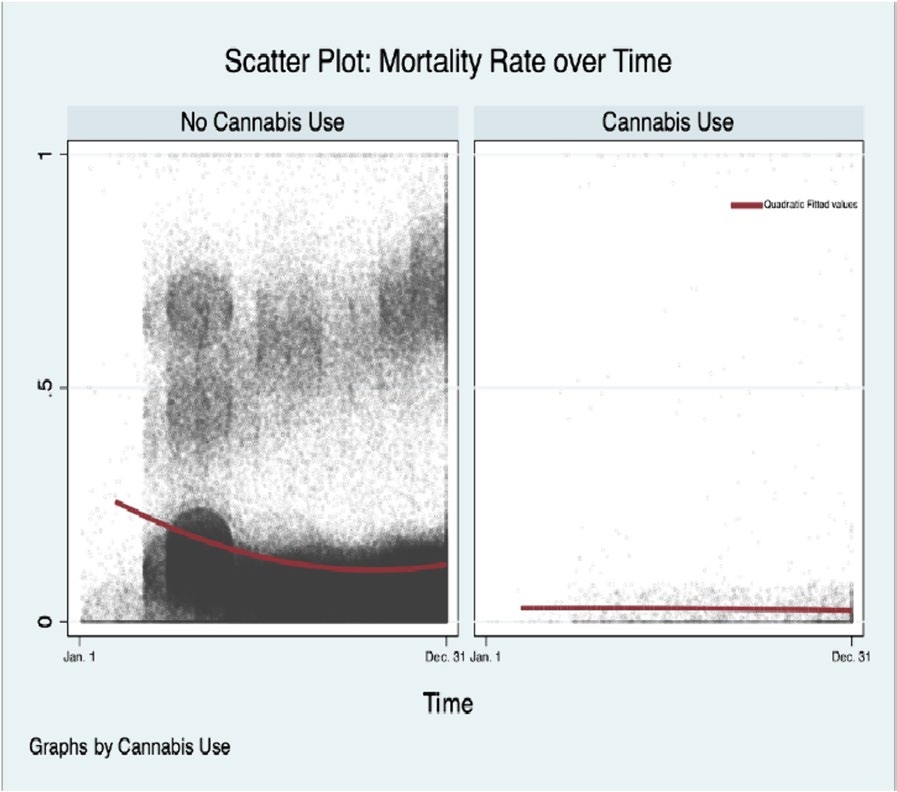
Mortality among Covid-19 patients over time. Legend: The mortality rate over time among all 2020 Covid-19 hospitalizations with and without cannabis use

**Fig. 2:**
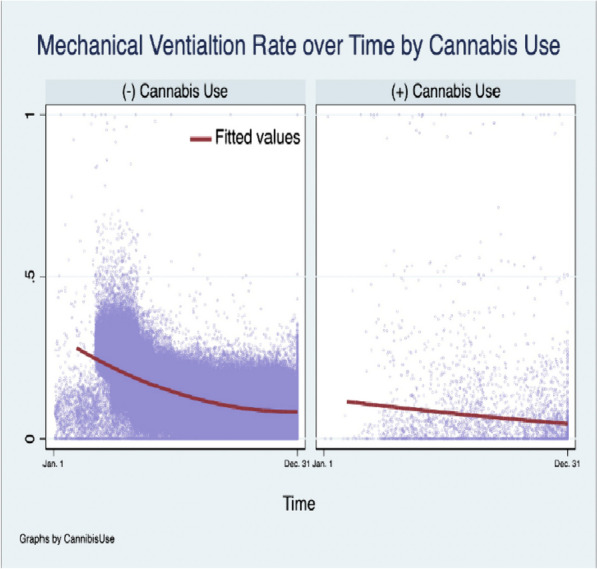
Mechanical ventilation among Covid-19 patients over time. Legend: The incidence of procedural mechanical ventilation over time of among 2020 Covid-19 hospitalizations with and without cannabis use

**Fig. 3:**
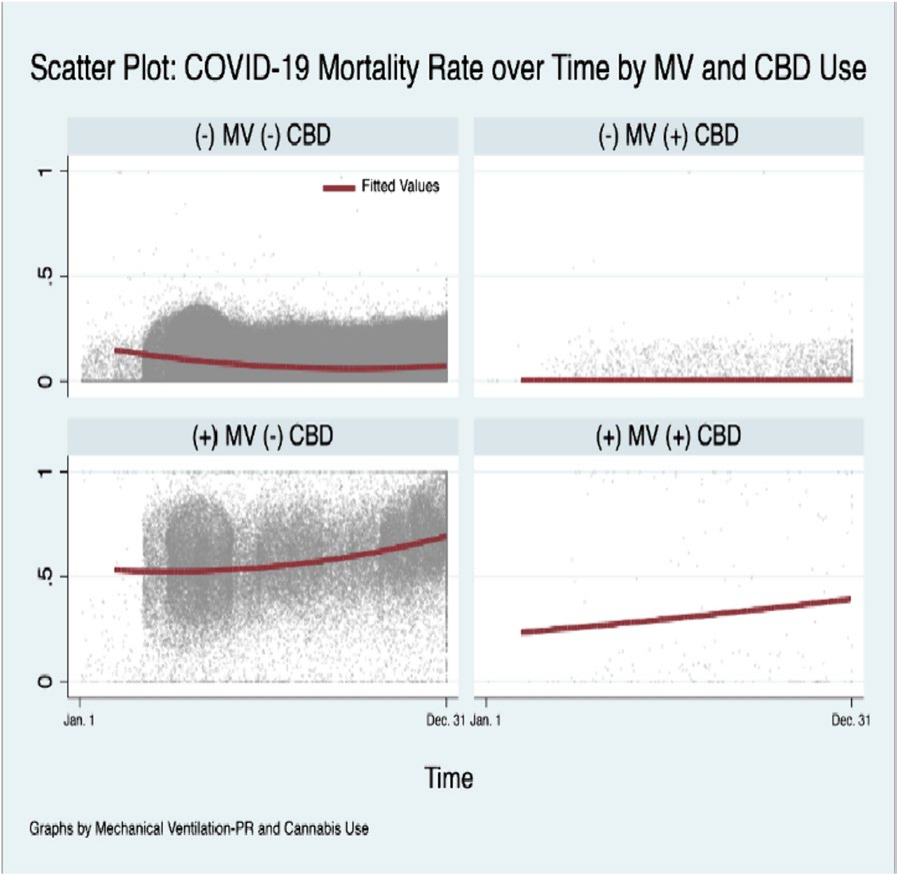
Mortality among Covid-19 patients over time by mechanical ventilation status and history of cannabis use. Legend: The mortality rate over time among all 2020 Covid-19 hospitalizations by mechanical ventilation status; with and without cannabis use. MV: Mechanical Ventilation. *CBD*: cannabis use as per ICD-10 coding

Amongst all-cause hospitalizations, CU was associated with a negative temporal relationship with Covid-19 hospitalization (Beta-Coefficient: -0.003, [-0.004, -0.001], *p* ≤ 0.001) and lower odds of Covid-19 related hospitalization from March to December during the index period controlling for other factors (aOR: 0.39, [0.37, 0.41], *p* ≤ 0.001); but a positive trend in mortality relative to N-CU within the Covid-19 subpopulation (Beta-Coeffient:0.01, [0.005, 0.02], *p* ≤ 0.001) (Supplement II Table 10). However, among Covid-19-related hospitalizations, the mortality rate for CU and N-CU in April and December was 3.47% vs 20.70%; and 2.10% vs 11.63%, respectively (Fig. 1). Moreover, within the Covid-19 subpopulation, there was a significant negative relationship between MV and CU over time (Beta-Coefficient: -0.02, [-0.03, -0.01], *p* ≤ 0.001) (Figs. 2 and 3). Among all-cause hospitalizations within the cohort, ACU-Dep (*N* = 82,685) was associated with significantly lower incidence of Covid-19-related hospitalizations compared to ACU-Abs (*N* = 783,940) during the index period (0.97% vs 1.61%, *p* ≤ 0.001, respectively). However, within the Covid-19 subpopulation, the incidence of MV was not significantly different for ACU-Dep (*N* = 805) compared to ACU-Abs (*N* = 12,660) (3.73% vs 7.03%, *p* = 0.11, respectively). Moreover, among Covid-19-CU patient encounters, the difference in mortality rate between ACU-Dep and ACU-Abs was not significant (1.24%vs 2.96%, *p* = 0.20, respectively).

Among MV-Covid-19 patients (MV-Covid-19) who received Remdesivir, cardiac arrhythmias, liver disease, or renal failure was associated with increased odds of death (aOR: 1.36 [1.22, 1.52], *p* ≤ 0.001; aOR: 1.74 [1.47, 2.06], *p* ≤ 0.001; aOR: 1.30 [1.10, 1.53], *p* ≤ 0.001, respectively). Moreover, coagulopathy was associated with higher odds of death, but long-term aspirin use (LT-ASA) or long-term anticoagulation was associated with lower odds of death compared to others (aOR: 1.34 [1.20, 1.51], *p* ≤ 0.001; aOR: 0.86 [0.74, 0.99], *p* = 0.04; aOR: 0.84 [0.71, 1.00], *p* = 0.05, respectively). Among non-mechanically ventilated Covid-19 patients (N-MV-Covid-19) and MV-Covid-19 patients, Remdesivir administration was associated with higher incidence of atrial fibrillation (AFib) (N-MV-Covid-19: 5.45% vs 4.72%, *p* ≤ 0.001, respectively; MV-Covid-19: 8.76% vs 7.10%, respectively, *p* ≤ 0.001); and within each respective group who received Remdesivir, atrial fibrillation (AFib) was associated with higher mortality compared to others (10.71% vs 5.08%, respectively; *p* ≤ 0.001; 9.54% vs 7.47%, respectively; *p* ≤ 0.001). Furthermore, among those who received Remdesivir, CAD (coronary artery disease) was associated with higher incidence and odds of AFib compared to others (N-MV: 11.61% vs 4.13%, *p* ≤ 0.001 MV:15.22% vs 7.25%, *p* ≤ 0.001; aOR: 1.36, [1.26, 1.47], *p* ≤ 0.001). Moreover, Remdesivir was also associated with significantly higher incidence of AFib among the MV-Covid-19, congestive heart failure (CHF) subpopulation (N-MV: 14.15% vs 12.93%, *p* ≤ 0.001, respectively; MV: 14.83% vs 12.54%, *p* = 0.005, respectively) (Supplement II Table 2).

When controlling for other factors in unmatched MRA among the Covid-19 population, ACU was associated with significantly lower odds of death (aOR: 0.45, [0.33, 0.61], *p* ≤ 0.001); MV (aOR: 0.80, [0.65, 0.98], *p* = 0.04); and acute pulmonary embolism compared to others (aOR: 0.56, [0.40, 0.78], *p* ≤ 0.001). Notably, obesity with complicated diabetes (Ob-DM) was associated with significantly higher odds of death compared to others (aOR: 1.21, [1.13, 1.29], *p* ≤ 0.001); while obesity alone was associated with lower odds of death (aOR: 0.86, [0.82, 0.91], *p* ≤ 0.001); and complicated diabetes alone was associated with nonsignificant lower odds of death (0.99, [0.95, 1.03], *p* = 0.55), controlling for other factors. Of note, the mortality rate among Covid-19- Ob-DM was significantly lower for CU (*N* = 640) compared to N-CU (*N* = 145,390) (6.25% vs 15.99%, *p* ≤ 0.001). Additionally, micronutrient (vitamin B12/folate, magnesium, or vitamin D) deficiency or deficiency anemia was associated with a variable, lower odds of primary and secondary outcomes compared to others (Supplement II Tables 6–10).

Finally, on PSM for *active cannabis use (ACU;* see Methods), active cannabis use (N(unweighted) = 2,382) was associated with 83.97% lower odds of death compared to those in the no active cannabis use group (N-ACU) (N(unweighted) = 282,085) (2.77% vs 3.95%, respectively; aOR:0.16, [0.10, 0.25], *p* ≤ 0.001; Absolute Risk Reduction (ARR): 1.84%) (Supplement II Table 11). Moreover, ACU (N(unweighted) = 2,382) was associated with lower odds of acute PE compared to others (N(unweighted) = 275,225) (Acute PE: 1.43% vs 2.27%, respectively; aOR:0.57, [0.39, 0.84], *p* = 0.004; Absolute Risk Reduction (ARR): 1.11%); and mechanical ventilation compared to others (N(unweighted) = 281,051) on matched analysis (Mechanical Ventilation: 6.93% vs 9.11%, respectively; aOR:0.81, [0.67, 0.99], *p* = 0.04; Absolute Risk Reduction (ARR): 1.13%; Supplement II Tables 12–15).

## Discussion

In our study, we demonstrate a significantly lower odds of mortality, MV, and acute PE among the cannabis use population compared to other Covid-19-related hospitalizations. The immunomodulatory role of cannabinoid receptor activation has been associated with induction of apoptosis, suppression of cell proliferation; a shift from Th1 to Th2 immune response and induction of regulatory T-cells; and induction of anti-inflammatory cytokine production and inhibition of proinflammatory cytokine production (Chayasirisobhon 2020; Laprairie et al. 2015; Pertwee et al. 2002; Pertwee 2008; Cosentino et al. 2022; Rieder et al. 2010; Rossi et al. 2020; Wang et al. 2020a; Howlett et al. 2002). The findings in our study demonstrate that the ECS may be a viable target for modulation of Covid-19 and suggest that cannabis use is protective against moderate to severe Covid-19-disease warranting hospitalization; and worse outcomes among the Covid-19 inpatient population. Notably, while the alpha variant of coronavirus was prevalent during the index study period, Nguyen et al. report that the effects of CBD were not related to inhibition of viral entry, but rather, induction of host innate immune response resulting in decreased induction of viral transcriptomic changes and production of viral protein, the effects of which were consistent across different variants of SARS-CoV-2 (Nguyen et al. 2002).

THC belongs to a family of plant-based phytocannabinoids with functional activity at the cannabinoid receptor as well as modulation of intracellular and extracellular calcium ion channels such as TRPV2, among others (Roth et al. 2015; Brailoiu et al. 2014; Bielawiec et al. 2020). However, other endogenous cannabinoids (arachidonoylethanolamide (anandamide [AEA]) and 2-arachidonoylglycerol (2-AG)), phytocannabinoids, and synthetic cannabinoids (such as JWH-015) also function as bioactive lipid mediators capable of modulating cannabinoid receptor responses within liver, skeletal muscle, heart, gut, bones, and adipose tissue (Bielawiec et al. 2020). Prior studies have demonstrated that cannabinoids exert their immunosuppressive properties through (1) induction of apoptosis, (2) inhibition of cell proliferation, (3) inhibition of cytokine and chemokine production, and (4) induction of regulatory T cells (T regs) (Nguyen et al. 2002; Roth et al. 2015; McKallip et al. 2002; Yuan et al. 2002). In this regard, modulation of the ECS and the immunosuppressive properties of phytocannabinoids within peripheral B-cell and T-cell lymphocytes may explain some of the findings reported in our study.

Notably, the production of endogenous cannabinoids may be dependent upon the availability of diet related precursors (Basavarajappa and Hungund 2005; Rouzer et al. 2023), and their functional modulation of cannabinoid receptors may be influenced by the density and coupling efficiencies of these and other receptors, receptor agonists, and other G-protein effectors (Pertwee 2008; Basavarajappa and Hungund 2005, 1999; Rouzer et al. 2023; Turner et al. 2017; Patel and Hillard 2006; Mińczuk et al. 2022). Additionally, the functional capacity of THC to antagonize full agonists of the cannabinoid receptors may also explain the partial protective effect noted among the cannabis use population and may relate to the partial effects of THC on viral host-transcriptomic modulation (Pertwee 2008; Nguyen et al. 2002; Patel and Hillard 2006; Mińczuk et al. 2022). Importantly, this central functional capacity may also relate to the functional target and mechanistic pathways utilized by SARS-CoV-2 itself (Mińczuk et al. 2022).

Nguyen et al. noted an induction of inositol-requiring enzyme 1α (IRE1α) RNase associated with endoplasmic reticulum (ER) stress response, induction of interferon pathways, as well as downregulation of cytokine and lymphokine production associated with CBD administration (Nguyen et al. 2002). Notably, de la Harpe et al. demonstrated that CBD induced ER stress response and the protective role of CBD in upregulation of unfolded protein response (URP) in breast cancer cell lines may relate to its activation of transient receptor protein vanilloid receptor 1 (TRPV1) channels and an associated increase in reactive oxygen species accumulation within the cell (de la Harpe et al. 2022). While THC itself may not interact with TRPV1 channels, there is evidence that THC and its primary metabolite THC-COOH are agonist of the transient receptor potential cation channel subfamily A member 1 (TRPA1) receptor which has been implicated in the development of fibrosis (Muller et al. 2019; Geiger et al. 2023). Unfortunately, the metabolite was not specifically tested by Nguyen’s team, and as such, its potential role in modulation of effects associated with SARS-CoV-2 infection through alternative pathways cannot be confirmed or excluded. Moreover, arylpyrazoles SR141716A, Gp-1a, and AM251, and the aminoalkyindoles WIN55,212-2 and AM630 were determined to activate and desensitize TRPA1 more potently than AEA (Muller et al. 2019).

Additionally, due to viral host-transcriptomic modification an associated URP response leading to an increase in activation of IRE1α, one of three stress sensing proteins within the ER, is not increased and subsequent cell death does not occur (Nguyen et al. 2002; Gregor and Hotamisligil 2007). However, while IRE1α activity itself is not upregulated by Covid-19, and, as noted by Nguyen and his colleagues, its absence among IRE1α knockout mice does not affect Covid-19 infection (Nguyen et al. 2002), the other two remaining stress-related proteins, PERK and activating transcription factor-6 (ATF-6), are not affected. The constitutional activation of PERK is associated with inhibition of translation of inhibitor of nuclear factor-κB (NFkB) (IkB) protein which in turn leads to NFkB induction of pro-inflammatory targets (Gregor and Hotamisligil 2007). Additionally, the UPR-induced upregulation of chaperone proteins leads to increased reactive oxygen species production and accumulation secondary to Ero1p and Erv2p enzyme-driven oxidation reduction reactions in the formation of disulfide bonds utilizing molecular oxygen as the final electron recipient, and these ROS themselves may either potentiate UPR activation or act as primary inducer of the UPR response. The induction of these inflammatory responses and the importance of inflammation in insulin resistance are known factors associated with comorbid obesity. Moreover, in X-box binding protein 1 (XBP-) (transcription factor involved in IRE1α-related induction of cell death) heterozygous mice–a corollary to pathways noted in Covid-19 disease in so far as XBP-1 is not activated due to inhibition of IRE1α pathways—exposed to high fat diet the result was hyperglycemia, hyperinsulinemia, and impaired glucose and insulin tolerance compared to wild-type mice, and, at the adipocyte itself, increased phosphorylation of PERK and IRE1α and increased c-Jun N-terminal kinase (JNK) activity, coupled with the loss of insulin sensitivity (Gregor and Hotamisligil 2007). Interestingly, free fatty acids (FFAs) themselves can induce JNK activation and subsequent insulin resistance in 3T3-L1 adipocytes (Gregor and Hotamisligil 2007; Nguyen et al. 2005). However, due to the localized, organ-specific, and state-specific prevalence of the CB1 and CB2 receptor and the heterogeneity of downstream signaling and associated effects, a full description of the pathophysiological mechanism of action has not yet been elucidated.

### Endocannabinoids, metabolism, and Covid-19 disease

The mitochondrial CB1 receptor has previously been described as an integral component involved in complex-I-dependent oxygen consumption, the alteration of which by THC reduces the intramitochondrial levels of cAMP and results in decreased protein kinase A (PKA)-dependent complex I phosphorylation and lowered mitochondrial respiration (Pagano Zottola et al. 2022). Moreover, prior research indicates that Covid-19 affects molecular pathways involved in energy metabolism (Nguyen et al. 2002; He et al. 2021; Govender et al. 2021; Chen et al. 2021; Al-Hakeim et al. 2023), and other reports note localization of viral targets within the ER and mitochondria (Nguyen et al. 2002; Nunn et al. 2022; Chen et al. 2023) as well as increased risk for insulin resistance during the acute-phase of infection and long-term following acute Covid-19 disease resolution (He et al. 2021; Govender et al. 2021; Chen et al. 2021; Al-Hakeim et al. 2023; Chandrashekhar Joshi and Pozzilli 2022). Aligned with these findings are reports indicating that the cannabinoid receptor localizes to punctate regions within the mitochondria and endoplasmic reticulum (Pagano Zottola et al. 2022; Castaneda et al. 2017; Hebert-Chatelain et al. 2016), and specifically cannabinoid receptor 1 distribution, prevalence, and function within white adipocytes among patients with obesity in whom there is a more significant baseline ER and mitochondrial dysfunction which is associated with increased activation of inflammatory transcription factors, many of which have been implicated in Covid-19 disease pathology (Nguyen et al. 2002; Gregor and Hotamisligil 2007; Pagano Zottola et al. 2022). Moreover, the significant benefit associated with LT-ASA (and long-term nonsteroidal anti-inflammatory drugs (NSAIDs); Supplement Table 6), as a member of the class of salicylates anti-inflammatory medications, may relate to its inhibition of the dephosphorylation of eukaryotic translational initiation factor 2A (eIF2A), the phosphorylation of which results in an increased alternative translation of ATF-4 inducing the expression of many genes, including those involved in apoptosis via PKR-like eukaryotic initiation factor 2α kinase (PERK), leading to sustained repression of protein synthesis and rescue from ER stress (Gregor and Hotamisligil 2007; Hebert-Chatelain et al. 2016; Silva et al. 2007; Hotamisligil 2010; Ausina et al. 2020; Hong et al. 2019; Wong et al. 2021).

What is more, the reported association between mitochondrial dysfunction in pathologic metabolic disorders such as obesity and diabetes and the pathological importance of the CB1 receptor in disease presentation (Mińczuk et al. 2022; Lee et al. 2019; Muller et al. 2017; D’Eon et al. 2008; Eckardt et al. 2009); the dose-dependent associated effects of THC on mitochondrial oxidative energy production and the modulation of insulin sensitivity or insulin resistance; and the functional modulation of the renin-angiotensin system associated with ECS modulation such that it decreases angiotensin-II expression highlight central mechanisms by which THC or other cannabinoids may interact and modulate viral infection (Mińczuk et al. 2022; Pagano Zottola et al. 2022; Hebert-Chatelain et al. 2016; Eckardt et al. 2009; Sarafian et al. 2003; Penner et al. 2013; Ngueta 2020; Ngueta and Ndjaboue 2020; Gallant et al. 2009; Tiyerili et al. 2010). The importance of mitochondrial CB1 activation and subsequent regulation of the intramitochondrial cAMP-PKA pathway in the functional activity of the SARS-CoV-2 infection may also explain the lower odds of primary and secondary outcomes noted among those predisposed to divalent cation malabsorption, and, more specifically, those with hypomagnesemia which is a critical cofactor for PKA functional activation (Supplement Tables 7–9) (Knape et al. 2015).

Nonetheless, the associated clinical outcomes associated with patients with obesity and complicated diabetes specifically may highlight the pathophysiological role of the CB1 receptor in disease modulation and align with these findings (Supplement Tables 6 and 7). This patient population specifically has been associated with a baseline dysfunctional regulation of URP and associated mitochondrial and ER stress response within adipocytes (Nguyen et al. 2002; Gregor and Hotamisligil 2007). Dysregulation of CB1-related pathways, alongside the introduction of SARS-CoV-2—which itself may be associated with derangements related to inflammatory pathways—may modulate infectivity and/or infection within visceral adipose tissue especially (Basolo et al. 2022; Moser et al. 2023).

Our findings align well with prior investigations analyzing the possible utility of the ECS in modulation of host innate antiviral response in the setting of Covid-19 disease (Nguyen et al. 2002; Mińczuk et al. 2022), and provide new evidence to support contention that the ECS may be effective in modulation of effects and mortality of Covid-19 disease (Shover et al. 2022; Cinar et al. 2022). Additionally, our findings align well with and validate recent previously mentioned findings (Nguyen et al. 2002). Additional research is needed to understand the mechanisms underlying these findings.

### Cannabis use, antiviral therapy, and contextualizing hospitalization

Analysis of factors associated with infection with SARS-CoV2 and associated mortality among hospitalized patients receiving antiviral therapy (Remdesivir) has not yet been fully elucidated. The Food and Drug Administration (FDA) issued an Emergency Use Authorization (EUA) for Baricitinib and Remdesivir in October 2020 to mitigate adverse outcomes among patients hospitalized to the hospital with Covid-19 if these patients had a new requirement for oxygen supplementation to maintain saturation. However, at the time, due to the circumstances around which the medication was authorized, an exhaustive analysis of adverse side effects among the Covid-19 population was not feasible (Food and Drug Administration 2020; Badgujar et al. 2020; Humeniuk et al. 2020; Beigel et al. 2020; de Wit et al. 2020; Fact sheet for healthcare providers: Emergency Use Authorization (EUA) of baricitinib n.d.; CDC 2022). Renal and hepatic impairment were both considerations for modification of dosing regimen; or contraindication to use as in Baricitinib in the setting of acute kidney injury. Among the subpopulation of patients receiving Remdesivir, we noted an increased odds of death associated with concomitant chronic liver and renal disease and indicates that close monitoring for adverse events is warranted among this subpopulation of patients.

Moreover, there was a significantly higher incidence of AFib among the Covid-19-Remdesivir and the CHF-Remdesivir subpopulations, with higher odds of death among the Covid-19-Remdesivir subpopulation with history of structural heart disease (Supplement II Table 6). These findings suggest that Remdesivir administration is associated with increased risk of cardiac arrythmia among susceptible patient populations and aligns with prior published reports of new-onset AFib or other cardiac arrythmia among Remdesivir-Covid-19 patients (Wang et al. 2020b; Gupta et al. 2020; Nabati and Parsaee 2022; Michaud et al. 2021; Rosenblatt et al. 2022; Devgun et al. 2023). Notably, there were lower odds of more severe complications associated with Covid-19 among the cannabis use population and younger patient encounters which may have been associated with the lower odds of Covid-19-related hospitalization or associated complications warranting the use of Remdesivir or Baricitinib. However, while the difference in mean age in years for ACU vs N-ACU in the matched sample (40.73y.o. vs 42.32y.o., *p* ≤ 0.001, respectively) was statistically significant different, this difference was not clinically significant, and mean *Age Group* was not significantly different between ACU vs N-ACU (2.00 vs 2.00, *p* = 0.78, respectively) (Table 3). As such, even accounting for age, obesity, diabetes, odds of cannabis use, and other factors in PSM, CU was associated with lower mortality compared to N-CU.

**Table 3:** Baseline characteristics and all-cause mortality after propensity score matching among Covid-19 hospitalizations with and without active cannabis use

**Variable:**	**Mean (Std. Err.)**		**t-test**	
	**ACU (*N* = 2,382)**	**No ACU (*N* = 282,085)**	***t***	***p* >| t |**
**Outcomes:**				
**Mortality**	0.02771	0.03946	-2.25	0.024
**Mechanical Ventilation (MV)**	0.06927	0.06087	1.17	0.24
**Acute Pulmonary Embolism (PE)**	0.01427	0.01595	-0.47	0.635
**Age in years at admission, y**^**a**^	40.733(0.33)	42.315(0.32)	-3.44	0.001
**Age group (Continuous variable 1–6)**	2.0063(0.02)	1.9987(0.02)	0.28	0.778
**Length of Stay in Days**	6.9353(0.26)	6.9374 (0.25)	-0.01	0.995
**Total Charges in US Dollars, $**	$71,652(2,937.96)	$72,346(3,253.98)	-0.16	0.874
**AGE Group 1**	0.32032(0.01)	0.31906(0.01)	0.09	0.926
**AGE Group 2**	0.44878(0.01)	0.45508(0.01)	-0.44	0.662
**AGE Group 3**	0.14442(0.01)	0.14442(0.01)	0	1
**AGE Group 4**	0.07893(0.01)	0.07389(0.01)	0.65	0.513
**AGE Group 5**	0.00588(0.002)	0.00462(0.01)	0.6	0.548
**AGE Group 6**	0.00168(0.001)	0.00294(0.01)	-0.91	0.365
**Racial or Ethnic Group: White**	0.39379(0.01)	0.41352(0.01)	-1.39	0.165
**Racial or Ethnic Group: Black/African American**	0.36314(0.01)	0.35516(0.01)	0.57	0.566
**Racial or Ethnic Group: Hispanic**	0.16541(0.01)	0.15617(0.01)	0.87	0.386
**Racial or Ethnic Group: Pacific Islander**	0.01427(0.002)	0.01092(0.003)	1.04	0.299
**Racial or Ethnic Group: Native American**	0.02729(0.003)	0.03065(0.004)	-0.69	0.49
**Racial or Ethnic Group: Other**	0.0361(0.004)	0.03359(0.004)	0.47	0.636
**Female**	0.34677(0.01)	0.35978(0.01)	-0.94	0.348
**Primary Payor: Medicare**	0.18556(0.01)	0.19479(0.01)	-0.81	0.417
**Primary Payor: Medicaid**	0.42485(0.01)	0.42779(0.01)	-0.21	0.838
**Primary Payor: Private Insurance**	0.233(0.01)	0.22376(0.01)	0.76	0.448
**Primary Payor: Self**	0.09278(0.01)	0.08816(0.01)	0.56	0.579
**Primary Payor: No Charge**	0.00588(0.002)	0.00546(0.01)	0.19	0.847
**Primary Payor: Other**	0.05793(0.01)	0.06003(0.01)	-0.31	0.759
**Hospital Bedsize 1: Small**	0.21914(0.01)	0.21075(0.01)	0.71	0.481
**Hospital Bedsize 2: Medium**	0.27288(0.01)	0.27204(0.01)	0.07	0.948
**Hospital Bedsize 3: Large**	0.50798(0.01)	0.51721(0.01)	-0.64	0.524
**Hospital Location/Teaching Status 1: Rural**	0.04114(0.004)	0.04156(0.004)	-0.07	0.942
**Hospital Location/Teaching Status 2: Urban Nonteaching**	0.144(0.01)	0.15155(0.01)	-0.73	0.463
**Hospital Location/Teaching Status 3: Urban Teaching**	0.81486(0.01)	0.80688(0.01)	0.7	0.482
**Hospital Region 1: Northeast**	0.17632(0.01)	0.18178(0.01)	-0.49	0.623
**Hospital Region 2: Midwest**	0.24433(0.01)	0.23971(0.01)	0.37	0.71
**Hospital Region 3: South**	0.35978(0.01)	0.35726(0.01)	0.18	0.856
**Hospital Region 4: West**	0.21956(0.01)	0.22124(0.01)	-0.14	0.889
**ZIPINC_QRTL1**	0.42569(0.01)	0.411(0.01)	1.03	0.304
**ZIPINC_QRTL2**	0.25105(0.01)	0.27624(0.01)	-1.97	0.049
**ZIPINC_QRTL3**	0.19186(0.01)	0.178(0.01)	1.23	0.218
**ZIPINC_QRTL4**	0.1314(0.01)	0.13476(0.01)	-0.34	0.733
**Admission on the Weekend**	0.26994(0.01)	0.28086(0.01)	-0.84	0.399
**Admission Month 1: January**	0.0042(0.001)	0.0021(0.001)	1.29	0.196
**Admission Month 2: February**	0.00462(0.001)	0.00672(0.002)	-0.96	0.335
**Admission Month 3: March**	0.02939(0.003)	0.02897(0.003)	0.09	0.931
**Admission Month 4: April**	0.07473(0.01)	0.07641(0.01)	-0.22	0.826
**Admission Month 5: May**	0.06591(0.01)	0.06171(0.01)	0.59	0.553
**Admission Month 6: June**	0.07557(0.01)	0.07431(0.01)	0.17	0.869
**Admission Month 7: July**	0.13056(0.01)	0.13728(0.01)	-0.68	0.496
**Admission Month 8: August**	0.0974(0.01)	0.09782(0.01)	-0.05	0.961
**Admission Month 9: September**	0.06801(0.01)	0.07137(0.01)	-0.46	0.649
**Admission Month 10: October**	0.08564(0.01)	0.08522(0.01)	0.05	0.959
**Admission Month 11: November**	0.17045(0.01)	0.17212(0.01)	-0.15	0.878
**Admission Month 12: December**	0.19353(0.01)	0.18598(0.01)	0.66	0.506
**Elixsum Median ≥ 4**	0.49748(0.01)	0.53065(0.01)	-2.29	0.022
**Congestive Heart Failure**	0.10663(0.01)	0.11041(0.01)	-0.42	0.675
**Cardiac Arrhythmias**	0.13644(0.01)	0.14777(0.01)	-1.12	0.263
**Valvular Disease**	0.02183(0.003)	0.02393(0.003)	-0.48	0.628
**Pulmonary Circulation Disorders**	0.03442(0.004)	0.03359(0.004)	0.16	0.873
**Peripheral Vascular Disorders**	0.02687(0.003)	0.02561(0.003)	0.27	0.786
**Hypertension Uncomplicated**	0.36566(0.01)	0.37406(0.01)	-0.6	0.548
**Paralysis**	0.01805(0.003)	0.02183(0.003)	-0.93	0.351
**Other Neurological Disorders**	0.13476(0.01)	0.13854(0.01)	-0.38	0.704
**Chronic Pulmonary Disease**	0.2204(0.01)	0.22544(0.01)	-0.42	0.676
**Diabetes Uncomplicated**	0.08732(0.01)	0.09362(0.01)	-0.76	0.449
**Diabetes Complicated**	0.15071(0.01)	0.14861(0.01)	0.2	0.839
**Hypothyroidism**	0.03904(0.004)	0.04828(0.004)	-1.56	0.119
**Renal Failure**	0.10705(0.01)	0.10621(0.01)	0.09	0.925
**Liver Disease**	0.08144(0.01)	0.09992(0.01)	-2.22	0.026
**Peptic Ulcer Disease without Bleeding**	0.0063(0.002)	0.00588(0.002)	0.19	0.852
**Rheumatoid Arthritis Collagen Vascular Disorder**	0.01763(0.003)	0.01763(0.003)	0	1
**Coagulopathy**	0.08354(0.01)	0.09866(0.01)	-1.81	0.07
**Obesity**	0.20865(0.01)	0.22124(0.01)	-1.06	0.29
**Weight Loss**	0.04534(0.004)	0.0445(0.004)	0.14	0.889
**Fluid and Electrolyte Disorders**	0.39337(0.01)	0.40218(0.01)	-0.62	0.534
**Blood Loss Anemia**	0.00252(0.001)	0.0042(0.001)	-1	0.317
**Deficiency Anemia**	0.03988(0.004)	0.03988(0.004)	0	1
**Alcohol Abuse**	0.17506(0.01)	0.17128(0.01)	0.34	0.73
**Psychoses**	0.14316(0.01)	0.13476(0.01)	0.84	0.402
**Depression**	0.20571(0.01)	0.20907(0.01)	-0.29	0.775
**Hypertension Complicated**	0.144(0.01)	0.1419(0.01)	0.21	0.836
**Aspirin**	0.07137(0.01)	0.06927(0.01)	0.28	0.777
**Long Term Anticoagulation**	0.0424(0.004)	0.0487(0.004)	-1.04	0.297
**Long Term NSAID**	0.01259(0.002)	0.00756(0.002)	1.74	0.082
**Long Term Steroid**	0.01175(0.002)	0.0105(0.002)	0.41	0.679
**Tobacco Use Disorder**	0.3283(0.01)	0.32242(0.01)	0.43	0.665
**Coronary Artery Disease**	0.07431(0.01)	0.06339(0.01)	1.49	0.137
**Acute Kidney Injury**	0.20949(0.01)	0.21201(0.01)	-0.21	0.831
**Remdesivir Administration**	0.09068(0.01)	0.09152(0.01)	-0.1	0.92
**Vasopressor Use**	0.01301(0.002)	0.01301(0.002)	0	1
**Hypomagnesemia**	0.04744(0.004)	0.04954(0.004)	-0.34	0.736
**Vitamin D Deficiency**	0.02225(0.003)	0.02855(0.003)	-1.38	0.167
**History of Bariatric Surgery**	0.00546(0.002)	0.00672(0.002)	-0.56	0.576
**Elixhauser Mean**^**b**^	3.8925(0.04)	3.118 (0.004)	12.7	≤ 0.001

*P* >|t|= 0: *p* ≤ 0.001

^a^Sample was matched for utilizing Age Group not Age

^b^Sample was matched for utilizing Population Median Elixhauser Score not Elixhauser Mean

The state-dependent functional activity of the ECS modulates downstream signaling pathways within the cell such that its effects and the plasticity of its responses within different organ systems represents an interplay between internal and external stimuli, rapidly altering receptor availability and expression patterns in response to physiological inflammatory perturbations (Roth et al. 2015; Castaneda et al. 2017; Capozzi et al. 2018). As such, the differences in Covid-19 related outcomes among the cannabis use population may be associated with the underlying functional activity of cannabis, its constituent cannabinoids, and its associated receptor interactions but cannot be determined with our dataset (Supplement II).

## Limitations

Our study outcome measures are applicable only to the inpatient setting, with associated outcomes applicable to inpatient estimates only. Additionally, the NIS does not identify individual patients, but patient-level encounters. As such, recurrent hospitalizations may appear as distinct observations (HCUP National Inpatient Sample (NIS) 2020). Variation in reporting of cannabis use over time associated with patient-related reporting or hospital-related coding practices; regional differences in the legalization of marijuana may have affected the incidence of cannabis use during the study period; and administrative coding inaccuracies and/or classification errors may have affected our analyses. As such, cannabis use may still be underreported among the inpatient population. In addition, the study identifies *cannabis use* based upon historical use/administration using ICD-10-CM codes, and cannabis was not administered directly during the hospitalization encounter. Due to limitations associated with the NIS dataset, we were not able to assess more granular patient-level related data during hospitalization encounters or to confirm cannabis use with a positive UDS; nor qualify the indication—medical vs nonmedical use, timing, dose, route—inhalation vs ingestion vs absorption (i.e., lotion, oils, bath salts)—or duration of cannabis use relative to SARS-CoV-2 infection prior to hospitalization. However, the significant difference in Covid-19-related hospitalizations between ACU-Dep and ACU-Abs suggests that the modulating effects of cannabis and its constituent cannabinoids regarding Covid-19 may be dose-related. Additionally, while there was significant variability in mortality rate across hospital divisions for N-ACU, there was no significant variability in mortality among Covid-19 encounters across divisions for ACU, which supports a functional, modulatory effect associated with cannabis use (results not shown). Moreover, the large sample size of associated patient encounters within the NIS is a primary strength of the analysis and increases external validity of the associated outcomes analyses. Additional prospective clinical studies are needed to validate these findings. Please see Supplement for additional information.

Notably, it has been reported that the concentrations of the constituent cannabinoids within commercially available products can vary widely by distributor and geographic location (Rouzer et al. 2023). Nguyen et al. report that 1500 mg daily of Epidiolex demonstrated a maximal concentration at 7 days for CBD and 7-OH-CBD of 1.7 and 0.56 μM, respectively, with further increases in maximal concentration associated with coadministration with high-fat meals (Nguyen et al. 2002). Prior reports also indicate that following acute use, THC is rapidly distributed to highly perfused organ systems such as the lungs, heart, brain, and liver, with subsequent concentration of Δ9-THC into fat with continued exposure to marijuana (Agurell et al. 1984). However, the method of administration does not significantly affect metabolism and excretion following acute use and subsequent tissue redistribution. Although differences in plasma half-life after acute use may vary depending upon the chronicity of use, the slow release of Δ9-THC and other cannabinoids from tissues and subsequent metabolism results in a long elimination half-life (Agurell et al. 1986; Huestis 2007; Basis for the Recommendation to Reschedule Marijuana into Schedule III of the Controlled Substances Act. U.S. Department of Health and Human Services 2023).

Moreover, in a recent report from the U.S. Department of Health and Human Services citing data from the Behavioral Risk Factor Surveillance System (BRFSS), among people with past-month marijuana use, mean frequency of use was 17 days/month, with half of respondents reporting that they used marijuana 20–30 days/month. Additionally, citing data from the National Survey on Drug Use and Health (NSDUH), the report noted that among people with past-year marijuana nonmedical use, approximately half of individuals reported nonmedical marijuana use an average < 5 days/month while another 30% reported nonmedical marijuana use for an average > 20 days/month, with past-year use of marijuana predictive of past-month use for 60–80% of respondents. In the BRFSS population with past-30-day marijuana use, near-daily use was more likely if the individual used marijuana for medical reasons; but medical-only use was less common (25% for medical-only use; 39% for medical and nonmedical use; and 36% for nonmedical use only) (Basis for the Recommendation to Reschedule Marijuana into Schedule III of the Controlled Substances Act. U.S. Department of Health and Human Services 2023). However, the utilization-adjusted rate of adverse outcomes involving use of nonmedical marijuana or comparator drugs has been noted to be consistently lower than the respective utilization-adjusted rates of adverse outcomes involving heroin, cocaine, and, for certain outcomes, other comparators (Basis for the Recommendation to Reschedule Marijuana into Schedule III of the Controlled Substances Act. U.S. Department of Health and Human Services 2023).

## Conclusion

This study marks the first attempt to examine active cannabis use and Covid-19 outcomes among people who use cannabis using a national inpatient sample database. The results reveal significantly lower odds of Covid-19-related hospitalization, MV, PE, and mortality among individuals with a history of cannabis use compared to those without such history. These findings underscore the potential of the ECS as a viable target for modulating moderate and severe Covid-19 cases. Additional prospective studies are needed to further explore and corroborate these findings. As medical and recreational uses of cannabis continue to gain acceptance across different patient populations over time, and as regulatory frameworks for cannabis-related research and access continue to evolve at both the State and federal levels, the findings of this study call for further investigation, particularly with regards to implications for patient outcomes (State Medical Marijuana Laws (National Conference of State Legislatures website) 2023; Centers for Disease Control and Prevention 2021; DeFilippis et al. 2020; Budney et al. 2019; Saunders 2017; Basis for the Recommendation to Reschedule Marijuana into Schedule III of the Controlled Substances Act. U.S. Department of Health and Human Services 2023; Sacco et al. 2022; Sacco 2022; The White House 2022).

### Supplementary Information

**Supplementary Material 1.**

## Data Availability

The dataset analyzed during the current study are available in the Healthcare Cost and Utilization Project (HCUP) repository, https://hcup-us.ahrq.gov/nisoverview.jsp (HCUP National Inpatient Sample (NIS) 2020).

## Abbreviations

2-AG: 2-Arachidonoylglycerol
ACTH: Adrenocorticotropic hormone
ACU: Active cannabis use
ACU-Abs: Active cannabis abuse
ACU-Dep: Active cannabis dependence
AEA: Arachidonoylethanolamide (anandamide)
AFib: Atrial fibrillation
ANOVA: Analysis of variance
APR-DRG: All-Patient Refined Diagnosis Related Groups
ARR: Absolute Risk Reduction
ATF-6: Activating transcription factor-6
CAD: Coronary artery disease
CB1: Cannabinoid receptor 1
CB2: Cannabinoid receptors 2
CBC: Cannabichromene
CBD: Cannabidiol
CBDA: Cannabidiolic acid
CBDV: Cannabidivarin or
CBG: Cannabigerol
CDC: Center for Disease Control
CHF: Congestive heart failure
Covid-19: Covid-19 disease
CU: Cannabis use
CU-Rem: Cannabis abuse in remission; cannabis dependence in remission
CUD: Cannabis use disorder
DRGs: Diagnosis Related Groups
eIF2a: Eukaryotic translational initiation factor 2a
ELIX: Population median Elixhauser-index sum score
ECS: Endocannabinoid system
ER: Endoplasmic reticulum
EUA: Emergency Use Authorization
FFAs: Fatty acids
FDA: Food and Drug Administration
HCUP: Healthcare Cost and Utilization Project
ICD-10 CM: International Classification of Diseases, Tenth Revision, Clinical Modification
ICD: International Classification of Diseases
IL: Interleukin
IkB: Inhibitor of nuclear factor-κB
IRE1α: Inositol-requiring enzyme 1α
JNK: C-Jun N-terminal kinase
LOS: Length of stay
LT-ASA: Long-term aspirin
MRA: Multivariable regression analysis
MV: Mechanical ventilation
NFkB: Nuclear factor-κB
N-ACU: No active cannabis use
N-CU: No cannabis use
NIS: National Inpatient Sample
NSAIDs: Nonsteroidal anti-inflammatory drugs
Ob-DM: Obesity with complicated diabetes
PBMC: Peripheral Blood Mononuclear Cells
PE: Pulmonary embolism
PERK: PKR-like eukaryotic initiation factor 2a kinase
PKA: Protein kinase A
PSM: Propensity score matched analysis
SARS-CoV-2: Severe acute respiratory syndrome coronavirus 2
THC: Δ9-Tetrahydrocannabinol/dronabinol
TOTCHG: Total charge
Tregs: Regulatory T cells
TRPA1: Transient receptor potential cation channel subfamily A member 1
TRPV1: Transient receptor protein vanilloid receptor 1
URP: Unfolded protein response
XBP: X-box binding protein
