# Supplementary material for: Cannabis use associated with lower mortality among hospitalized Covid-19 patients using the national inpatient sample: an epidemiological study
Source: J Cannabis Res. 2024 Apr 6;6:18. doi: 10.1186/s42238-024-00228-w (PMC10998318; doi:10.1186/s42238-024-00228-w)
Supplement: Supplementary file 1 — Supplementary Material 1. [file 42238_2024_228_MOESM1_ESM.docx]

**Online Supplement**

**eAppendix 1. ICD-10 Coding**

**eAppendix 2. Data Source**

**eAppendix 3. Definitions and Study Population**

**eAppendix 4. Statistical Analysis, Tables, and Figures**

**eAppendix 1.**

***ICD-10 CM (Clinical Modification) Diagnosis Codes:***

SARS-CoV-2 (***Covid-19):***

U.071

B97.29-- Coronavirus (infection) as cause of disease classified elsewhere

***Acute Pulmonary Embolism:*** I2609, I2602, I2692, I2699, O8822, O8823, O8882, O8883, O88811, O88812, O88813, O88819 ***Long-Term (current) Aspirin Use:*** Z7982

***Long-Term use Antiplatelet/Antithrombotic:*** Z79.02

***Long-term NSAID Use:*** Z79.1

***Long-Term (current) Steroid Use:*** Z79.52

***Long-Term (current) Anticoagulation:*** Z79.01

***Schizophrenia:*** F200, F201, F202, F203, F205, F2081, F2089, F209

***Morbid Obesity:*** E6601, Z6841, Z6842, Z6843, Z6844, Z6845

***Coronary Artery Disease/ Chronic ischemic heart disease:*** I25.10. I25.110, I25.111, I25.112, I25.118, I25.119

***Tobacco Use Disorder:*** F17203, F17208, F17209, F17210, F17211, F17213, F17218, F17219, F17220, F17221, F17223, F17228, F17290, F17291, F17293, F17298, F17299, F17.2, F17.203, F17.208, F17.209, F17.21, F17.210, F17.211, F17.213, F17.218, F17.219, F17.22, F17.220, F17.221, F17.223, F17.228, F17.29, F17.290, F17.291, F17.293, F17.298, F17.299

***Aflutter:*** I484, I483, I4892

***Atrial Fibrillation:*** I480, I4811, I4819, I4820, I4821, I4891

***New-Onset (Paroxysmal) Atrial Fibrillation:*** I480

***Cardiac Arrest/Ventricular Fibrillation/Flutter:*** I462, I468, I469

***Chronic Systolic Heart Failure:*** I502, I50814, I5082, I5084, I5084

***Acute on Chronic Systolic Heart Failure:*** I5021, I5023

***Acute on Chronic Diastolic Heart Failure:*** I5031, I5033

***Chronic Diastolic Heart Failure:*** I5032

***History of Bariatric Surgery:*** Z9884

***Vitamin D Deficiency:*** E559

***Vitamin B12 Deficiency:*** D510, D511, D512, D513, D518, D520, D521, D528, D529, D531, D519

***Homocysteinemia:*** E7211

***All-Use Cannabis Use (Use):***

F1210, F1211, F12120, F12121, F12122, F12129, F1213, F12150, F12151, F12159, F12180, F12188, F1219, F1220, F1221, F12220, F12221, F12222, F12229, F1223, F12250, F12251, F12259, F1228, F12280, F12288, F1290, F1291, F12920, F12921, F12922, F12929, F1293, F12950, F12951, F12959, F12980, F12988, F1299

***Cannabis Abuse:***

F1210, F1211, F12120, F12121, F12122, F12129, F1213, F12150, F12151, F12159, F12180, F12188, F1219, F1290, F1291, F12920, F12921, F12922, F12929, F1293, F12950, F12951, F12959, F12980, F12988, F1299

***Cannabis Dependence:***

F1220, F1221, F12220, F12221, F12222, F12229, F1223, F12250, F12251, F12259, F1228, F12280, F12288

***Active Cannabis Use (ACU):***

F1210, F12120, F12121, F12122, F12129, F1213, F12150, F12151, F12159, F12180, F12188, F1219, F1290, F12920, F12921, F12922, F12929, F1293, F12950, F12951, F12959, F12980, F12988, F1299, F1220, F12220, F12221, F12222, F12229, F1223, F12250, F12251, F12259, F1228, F12280, F12288

***Active Cannabis Abuse (ACU-Abs):***

F1210, F12120, F12121, F12122, F12129, F1213, F12150, F12151, F12159, F12180, F12188, F1219, F1290, F12920, F12921, F12922, F12929, F1293, F12950, F12951, F12959, F12980, F12988, F1299

***Active Cannabis Dependence (ACU-Dep):***

F1220, F12220, F12221, F12222, F12229, F1223, F12250, F12251, F12259, F1228, F12280, F12288

***Cannabis Abuse in Remission:*** F1211, F1291

***Cannabis Dependence in Remission:*** F1221

***ICD-10- PCS (Procedure Coding System) Procedure Codes:***

***Mechanical Ventilation:*** 5A1935Z, 5A1945Z, 5A1955Z

***Vasopressor Use:*** 3E030XZ, 3E060XZ, 3E063XZ, 3E053XZ, 3E050XZ, 3E043XZ, 3E040XZ, 3E033XZ

***Central Line Insertion:*** 05HM03Z, 05HM0DZ, 05HM33Z, 05HM3DZ, 05HM43Z, 05HM4DZ, 05HN03Z, 05HN0DZ, 05HN33Z, 05HN3DZ, 05HN43Z, 05HN4DZ, 05HP03Z, 05HP0DZ, 05HP33Z, 05HP3DZ, 05HP43Z, 05HP4DZ, 05HQ03Z, 05HQ0DZ, 05HQ33Z, 05HQ3DZ, 05HQ43Z, 05HQ4DZ, 05H503Z, 05H50DZ, 05H533Z, 05H53DZ, 05H543Z, 05H54DZ, 05H603Z, 05H60DZ, 05H633Z, 05H63DZ, 05H643Z, 05H64DZ

***Antiviral Medication Therapy:***

**Remdesivir Medication Therapy-Peripheral Vein Administration:** XW033E5

**Remdesivir Medication Therapy-Central Vein Administration:** XW043E5

**Baricitinib Medication Therapy:** XW0DXF5, 3E0G7GC

**eAppendix 2. Data Source**

***Data Source:***

***Complex Sampling Design of the National Inpatient Sample (NIS) Database:***

The study cohort was sampled from the National Inpatient Sample (NIS) 2020 database. The NIS is the largest publicly available all-payer, inpatient healthcare database designed to produce United States (U.S.) regional and national estimates of inpatient utilization, access, charges, quality, and outcomes. The NIS is sponsored by the Agency of Healthcare Research and Quality (AHRQ) and developed by the Healthcare Cost and Utilizations Project (HCUP). The HCUP is a family of databases, software tools, and related research products that enable research on a variety of healthcare topics.^48^

The NIS sample unit is a systematic random sample of discharges stratified by hospital characteristics drawn from all HCUP-participating hospitals. This sample includes approximately 20% of discharges from US community hospitals. Prior to 2012, the NIS was constructed annually by incorporating 100% of hospital discharges from 20% of U.S. hospitals. In 2012, the sampling design was reconstructed as a 20% national patient-level sample, with non-representative sampling across hospitals. This type of sampling design, referred to as a stratified systematic random sample, is systematically drawn from a list of discharges sorted on discharge characteristics such as DRG and admission month to ensure a more representative sample of discharges than a simple random sample would yield. These changes were associated with corresponding changes to sampling weights for patient encounters to estimate nationally representative outcome analysis. To account for the complex sampling design of the NIS, analysis utilized the variables DISCWT and HOSP_NIS for discharges in 2020.^48^

The *NIS Target Universe* for the NIS, is all US community hospital discharges and is defined from the American Hospital Association (AHA) Annual Survey of Hospitals. The *NIS Sample Frame* is the State Inpatient Databases which includes over 95% of the target universe. The *NIS Sample Strata* which were used in creating the NIS are U.S. census division, urban or rural location, teaching status, ownership, and bed size. Notably, state-level analyses cannot be conducted because the strata were not designed with “state” as a stratification variable. State level analyses can be performed using the State-specific SID. Additional information can be found on the official website of the Healthcare Cost and Utilization Project.^48^

The Diagnosis Related Groups (DRGs) are a patient classification scheme and provides a means of relating the type of patients a hospital treats (i.e., its case mix) to the costs incurred by the hospital. Three major versions of DRG are currently in use: basic DRGs, All Patient DRGs, and All Patient Refined DRGs. The Centers for Medicare and Medicaid Services (CMS) uses the basic DRGs for hospital payment for Medicare beneficiaries. To provide more representative data of non-Medicare populations, the All Patient DRGs (AP-DRGs) are an expansion of the basic DRGs. The All Patient Refined DRGs (APR-DRG) incorporate severity of illness subclasses into the AP-DRGs. Moreover, the term case-mix complexity has been used to refer to an interrelated but distinct set of patient attributes which include severity of illness, risk of dying, prognosis, treatment difficulty, need for intervention, and resource intensity. The APDRG Severity of Illness Subclass (APDRG-S) refers to the extent of physiologic decompensation or organ system loss of function, and it has been noted in prior studies to model risk of mortality.^49-51^

**eAppendix 3. Definitions and Study Population**

***Utilization of Covid-19 (*U.071 *and* B97.29) *Disease Coding over Time:***

The CDC (Centers for Disease Control) officially activated ICD10-CM U.071 in April 2020. However, the World Health Organization (WHO) released information concerning the creation of International Classification of Disease version 10 (ICD-10) coding for Covid-19 disease in March 2020. We chose to utilize January instead of April in defining the Covid-19 characterization because:

1) The CDC reports that the first laboratory confirmed case of the 2019 Novel Coronavirus (Covid-19) in the U.S. was on January 18 in Washington state; ^5^ and within the NIS dataset 42,315 hospitalizations nationally, before April 2020 were associated with B97.29 (January: 3,495 hospitalizations; February: 2,575 hospitalizations; March: 32,150 hospitalizations)^47^

2) The WHO (World Health Organization) Emergency Activation of ICD-10 U07.1 occurred in March 2020. There was a significant difference in utilization of U.071 for inpatient hospitalizations in the month of March compared to January or February (p≤0.001; p≤0.001); and 59.71% (47,760/79,795) of the Covid-19 inpatient population in March 2020 contained ICD-10-CM coding indicating U07.1. The mortality associated with this patient population was 24.88% compared to that of all-cause hospitalizations 2.86%, (p≤0.001); and compared to that of the mortality associated with Covid-19 vs all-cause hospitalizations in April 2020, 20.54% vs 2.48% (p≤0.001). The mortality associated with Covid-19 U.071 in January of 2020 was 18.75% vs 2.51%, and 21.95% vs 2.44% in February. Mortality rate within the Covid-19 population was not significantly different between January and February; but for each month thereafter, the mortality rate associated with Covid-19 was significantly greater than either January or February (p≤0.05). However, these hospitalizations represent a negligible 0.017% of the total Covid-19 related hospitalizations reported within the dataset (290/1,698,560). It is unclear if these patients represent Covid-19 positive patients as the means of testing for the SARS-CoV2 infection were not well developed at that time. Nor is it clear if ICD coding records may be backlogged using updated coding information guidance from the CDC/CMS for those hospitals sampled within the NIS.

3) Guidance from the CDC recommended utilization of Covid-19 infections to B97.29 and following activation of the ICD-10-CM U07.1, to indicate associated complications for patients for whom Covid-19 pneumonia was not the primary indication for admission, but ancillary to primary inpatient admission indication.

Covid-19 disease U07.1 as primary diagnosis on a medical claim during hospitalization was utilized in differentiating primary admitting diagnosis of Covid-19 compared to patients diagnosed with Covid-19 following admission, corresponding to Covid-19 diagnosis in secondary or other positions besides 1 on a medical claim.

The associated outcomes related to COVID-19 vs non-COVID-19 hospitalization can be found in the supplementary figures and tables below (Supplementary Table 1-3; Supplementary Figure 1).

***Cannabis Use:***

*Cannabis use (CU)* was defined as *cannabis abuse* or *cannabis dependence*. *Cannabis use, unspecified* was included in the group *cannabis abuse.* *Cannabis abuse* and *cannabis dependence* diagnoses were identified using ICD-10-CM diagnosis code corresponding to *cannabis abuse* or *cannabis dependence* in any position on a medical claim, inclusive of inpatient hospital encounters and can be found in eAppendix 1. C*annabis abuse* was further stratified into *cannabis abuse not in remission (ACU-Abs)* and *cannabis abuse in remission*; and *cannabis dependence* was stratified into *cannabis dependence not in remission (ACU-Dep)* and *cannabis dependence in remission*. For the purposes of this article, *active cannabis use (ACU)* was defined as *ACU-Abs* or *ACU-Dep* and excluded *cannabis abuse in remission* and *cannabis dependence in remission.* The term active was used specifically to identify patients who were not in remission to increase the specificity of the terms used and improve validity of the associated outcome analyses. The corresponding ICD-10-CM diagnoses codes can be found in eAppendix 1.

**Selection of Variables:**
Variables were selected based upon stratification from Elixhauser classifications, and/or known clinical or confounding factors associated with conditions of interest. The variables selected controlled for hospital-level characteristics (hospital region, hospital location and teaching status, hospital bed size), admission-level differences such as admission on the weekend; patient level characteristics such as income, age, insurance payor, race, gender, and sex; and comorbid conditions which may each represent potential confounders of the described analysis. Please note Abe et al. and references.^23,1-22,24-100^

**Primary vs Secondary Covid-19 Diagnosis: Cannabis Use (CU) vs Non-Cannabis Use (N-CU):**

Of note, approximately 39.78% (675,700) of patients diagnosed with Covid-19 contracted SARS-CoV2 infection following admission, with an associated higher mortality rate among said population (17.26%) compared to those admitted with a primary diagnosis of Covid-19 (10.91%) during the index period (p≤0.001). Moreover, among all-cause hospitalizations without primary diagnosis of Covid-19, CU was associated with significantly lower incidence of Covid-19 following admission (non-primary diagnosis) compared to others (0.97% vs 2.41%, p≤0.001). What is more, within the Covid-19 subpopulation, cannabis use (CU) was associated with significantly higher incidence of non-primary Covid-19 diagnosis compared to those without cannabis use (N-CU) (63.20% vs 39.59%, p≤0.001), and the associated mortality for Covid-19 *primary* or *non-primary* diagnosis was significantly lower for CU compared to N-CU (2.99% vs 10.96%, p≤0.001; 2.82% vs 17.45%, p≤0.001). Covid-19-related mean length of stay (LOS) and total charge (TOTCHG) (7.97±0.03 days; $91,023.02±1212.76) was significantly higher than that of N-COVID patients (4.89 ± 0.02 days; $65,007.06) (p≤0.001 for both) (Supplement Table 2). The mean length of stay (LOS) and mean total charge (TOTCHG) for those admitted with primary diagnosis of Covid-19 for the ACU vs N-ACU was 6.18 +/-0.27 days vs 7.49+/-0.3 days, p≤0.001; $67,003.5 +/- 4485.64 vs $78,641.52 +/- 11,24.974, p=0.015, respectively. The mean LOS and TOTCHG for those admitted with secondary diagnosis of Covid-19 for the ACU vs N-ACU was 7.70 +/- 0.44 days vs 8.77+/- 0.5 days, p≤0.001; $77,267.13 +/- 4,299.48, p≤0.001 vs $111,690.90 +/- 1,709.078, respectively.

Flow Diagram of the patient population can be found in *Supplement Figure 3* below.

**Remdesivir and Baricitinib Administration:**
Antiviral medication therapy (Remdesivir) utilization was identified during the inpatient hospital encounter with either an ICD-10 procedure code corresponding to Remdesivir administration in the peripheral (*P-Remdesivir*) or central (*C-Remdesivir*) vein (XW033E5 or XW043E5, respectively). Baricitinib medication therapy was identified during the inpatient hospital encounter with either an ICD-10 procedure code corresponding XW0DXF5 or 3E0G7GC (Supplemental Figure 2).

**New-onset Atrial Fibrillation:**

New-onset atrial fibrillation was identified using ICD-10-CM diagnosis code I480 (*paroxysmal atrial fibrillation)* and excluded other types of dysrhythmias.

**Endocannabinoid System (ECS) Additional Information:**

The differential localization of endocannabinoid receptors has been reported to shift during maturation and/or activation of immune cells.^62^ Castaneda et al. note a downregulation of external CB2 receptors for activated cells of B cell lineage and describe how tissue samples collected from different immune sites display differences in CB2 staining dependent upon naivety of the cell, with more mature cells displaying a marked decreases in extracellular CB2 receptors compared to intracellular CB2 receptors and similar changes in CB2 expression patterns among B-cell lymphoma malignancies.^63^ Additionally, Castaneda and Roth et al. also note that T cells, monocytes, and dendritic cells exhibited only intracellular CB2 and a capacity for increasing CB2 receptor availability among activated T lymphocytes which was associated with a decreased propensity for proliferation, decreased IL-2 and TNF-alpha production, and increased IL-5 and IL-10 production and Th2 phenotype.^63-64^

**Regional Cannabis Use among 2020 Covid-19 Hospitalizations:**

There were significant differences in the incidence of active cannabis use (ACU) disorder between regions and hospital divisions. Hospital region 3(South) associated with negative trend in the incidence of ACU over time compared to hospital region 1(Northeast) when controlling for other factors (Beta-coefficient: -0.0016, [-0.0027, -0.0005], p=0.005); and hospital region 4(West) associated with positive trend in the incidence of ACU over time compared to hospital region 1(Northeast) when controlling for other factors (Beta-coefficient: 0.0016, [0.0002, 0.0030], p=0.026). Only hospital division 6 (East South Central) was associated with negative trend in the incidence of ACU over time compared to hospital division 1(New England) when controlling for other factors (Beta-coefficient: -0.0030, [-0.0056, -0.0005], p=0.019).

When stratified and controlling for other factors, hospital region 3(South) was associated with a negative trend in the incidence of active cannabis abuse(ACU-Abs) over time compared to hospital region 1(Northeast) (Beta-coefficient: -0.0015**, [**-0.0026, -0.0004], p=0.007); and hospital region 4(west) was associated with significantly higher incidence of ACU-Abs over time compared to hospital region 1 (Beta-coefficient: 0.0016, [0.0002, 0.0029], p=0.020). Additionally, across divisions, only hospital division 6 (East South Central) was there a significantly negative trend in the incidence of ACU-Abs compared to hospital division 1(New England) over time (Beta-Coefficient: -0.0030, [-0.0055, -0.0006], p=0.016). There were no significant differences in the incidence of active cannabis dependence (ACU-Dep) across regions over time. However, across divisions, only for hospital division 6 (West North Central) was there a positive trend in the incidence of ACU-Dep compared to hospital division 1(New England) over time (Beta-Coefficient: 0.0008, [0.0001, 0.0014], p=0.020). There were no significant differences in the incidence of cannabis use in remission (CU-Rem), defined as cannabis abuse in remission or cannabis dependence in remission, across regions or divisions over time. However, within regions and divisions, there appear to be differences in ACU-Abs and ACU-Dep hospital discharges over time during the 2020 Covid-19 Pandemic (Supplemental Figures 4-6).

**Additional Demographic Characteristics and Outcomes: Cannabis Use (CU), Active Cannabis Use (ACU), Cannabis Use in Remission (CU-Rem) and No Cannabis Use (N-CU)**

The percent proportion of the population within APDRG-S 3&4 compared to APDRG-S 1&2 was 8.87 percentage points(pp) lower for CU (85.42%) compared to N-CU (94.29%) (p≤0.001) (Table 2). However, the mortality rate among patients within the APDRG-S 3&4 subclass was 10.84 pp lower for CU (3.36%) compared to N-CU (14.21%) (p≤0.001). Within White, Black, Hispanic, Native American, and Other race groups, mean mortality was significantly lower for those who used cannabis compared to non-users (10.53pp, 9.60pp, 10.72pp, 15.25pp, 11.21pp; p≤0.001); and 11.41pp lower among Asian/Pacific Islanders (p=0.058). Additionally, while the mean percentage of uncomplicated or complicated diabetes, morbid obesity, and coronary artery disease were significantly lower for CU compared to N-CU (p≤0.001 for all), the mean Elixhauser index sum was 0.30 units greater for CU compared to N-CU (p≤0.001); and the mortality rate within each aforementioned comorbidity subgroup was 9.02pp, 11.82pp, 6.78pp, and 12.49pp lower for CU compared to N-CU, respectively (p≤0.001 for all). Moreover, active cannabis use (ACU) itself was associated with lower incidence of septic shock compared to other Covid-19 patient encounters (2.97% vs 7.33%, p≤0.001).

Moreover, on PSM analysis matched for ACU vs long-term NSAID (Nonsteroidal Anti-inflammatory Drug) use (excluding patients receiving aspirin); vs long-term steroid use odds of mortality for CBD were significantly lower than matched controls (aOR: 0.14, [0.05-0.35], p≤0.001; aOR: 0.18, [0.09-0.33], p≤0.001, respectively); and not significantly different from long-term anticoagulation (aOR: 0.69, [0.44-1.09], p=0.12).

Notably, among the Covid-19 population, the incidence of mechanical ventilation (MV) and acute pulmonary embolism for those with cannabis use in remission (CU-Rem; N=285), defined as either cannabis abuse in remission or cannabis dependence in remission, was not significantly different from the general population, excluding the active cannabis use (ACU) population (5.26% vs 12.09%, p=0.11; 3.51% vs 2.37%, p=0.57, respectively); nor the odds of acute PE or MV (aOR: 0.67, [0.089, 5.01], p=0.69; aOR: 0.73, [0.21, 2.59], p=0.63, respectively). However, mortality was significantly lower for those with CU-Rem compared to the general population, excluding the ACU population (3.51% vs 13.52%, p=0.03, respectively; OR: 0.23, [0.06, 0.95], p=0.04); but significance attenuated in the complete, unmatched multivariable logistic regression (aOR: 0.94, [0.22, 3.94], p=0.93).

**Covid-19 CU vs N-CU: Structural Heart Disease:**

Active cannabis use was associated with significantly higher odds of acute decompensated systolic heart failure compared to others within the Covid-19 subpopulation (Supplement Table 5). However, excluding those who received Remdesivir, there was a significantly lower mortality among ACU (N=260) compared to other Covid-19 encounters (N=22,090) within the ADCHF-Syst subpopulation (7.69% vs 27.21%, p=0.002, respectively); or within the Covid-19 congestive heart failure subpopulation generally for ACU (N=1,295) compared to Remdesivir (N= 65,160) (6.18% vs 22.59%, p≤0.001). In contrast, excluding CU, Remdesivir (N=5,775) was not associated with significantly different mortality compared to other Covid-19 encounters (N=22,090) within the ADCHF-Syst subpopulation (27.62% vs 27.21%, p=0.78, respectively). What is more, among MV-Covid-19, there were 920 patient encounters associated with ACU, 190 of whom also received Remdesivir; and 47,830 patients received Remdesivir without reported use of CU. Among MV-Covid-19 and excluding those who received Remdesivir and reported active cannabis use, ACU (N=730) was associated with a 28.77% mortality rate compared to 62.57% among those who received Remdesivir (N=47,830) (p≤0.001). In contrast, among MV-Covid-19 who received Remdesivir, ACU (N=190) was associated with a nonsignificant lower mortality compared to others (N= 47,830) (50.00% vs 62.57%, p=0.11, respectively). There was limited use of Baricitinib among the cannabis use population and similar analysis was not possible among this population of patients.

**eAppendix 4. *Statistical Analysis:***

**Propensity Score Matching (PSM):**

Matching design used was 1:1 nearest neighbor with replacement using a 0.1 caliper width with common support and odds of receiving treatment (odds match on the logarithm of the odds ratio of the propensity score). Patient encounters were matched on *Age Group*, *length of stay group*, race, sex, admission month, hospital characteristics: hospital region, hospital bed size, location/teaching status of hospital; primary insurance payor: Medicare, Medicaid, Self-Pay, Other, No Charge; Median household income for patient's ZIP Code (based on current year), population median-Elixhauser-index sum score 4 or more, 25 Elixhauser comorbid conditions (patients with lymphoma, AIDS/HIV, metastatic cancer, and solid tumor without metastasis were excluded from the PSM analysis); coronary artery disease; tobacco use disorder; acute kidney injury; diagnosis of hypomagnesemia; diagnosis of vitamin D deficiency; history of bariatric surgery; and long-term medications (aspirin, steroids, anticoagulation, NSAIDs), vasopressor use, mechanical ventilation, acute kidney injury (AKI), Remdesivir use, and others as noted in the study (Table 3). Because patients were matched on pulmonary circulation disorders and mechanical ventilation status within the matched sample for outcome death, two separate PSM were utilized for the secondary outcome: acute pulmonary embolism (PE) and mechanical ventilation (MV). Matching covariates utilized were the same for both secondary outcomes except for *Pulmonary Circulation Disorders* which was not included in the match for acute PE only (Supplementary Table 12-15). Variables were not matched on total charge (TOTCHG), however, matched sample TOTCHG is reported in Table 3 and Supplementary Table 12 and 14. The propensity score matching utilized the STATA code *psmatch2* which performs full Mahalanobis and propensity score matching, common support graphing, and covariate imbalance testing, and the *average treatment effect* (ATE) reported utilized the code *teffects psmatch*. Double robust results following PSM were achieved by multivariate logistic regression on the *psmatch2* matched sample using the same matching covariates; and used the frequency weights derived from the matching procedure. STATA/MP software (StataCorp. 2021. Stata Statistical Software: Release 17. College Station, TX: StataCorp LLC) was used for all analyses. Demographic breakdown of the primary outcome PSM population can be found in Table 3 of the main manuscript; and Supplementary Table 12 and Supplementary Table 14 below.

Shover et. al. previously demonstrated lower severity among cannabis users with Covid-19 disease among a small cohort of patients admitted to two medical centers in Southern California.^26^ The present study utilizes an alternate database with greater control for patient co-morbid conditions, and it diverges from Shover in primary outcome results. The sample size of this previous study was significantly smaller than our current study. Moreover, the demographic population utilized in Shover et. al. was sampled from locations which significantly confound outcomes analysis. In our study, we found that those within National Inpatient Sample (NIS) Hospital Division: Division 9 (Pacific) or Hospital Region: West was associated with significantly lower odds of Covid-19 related hospitalization and other outcomes compared to other divisions or regions, respectively (Hospital Division results not shown; Hospital Regions results shown in Supplementary Table 10 below). As such, these outcomes reported by Shover et. al. have unidentified and unaccounted for confounders related to hospital region for which our analysis controls.

Multivariable logistic regression was performed following propensity score matching on the matched sample utilized the variables above for primary and secondary outcomes (Supplementary Table 11, 13, and 15 below).  In the matched sample for secondary outcome: acute pulmonary embolism (PE), *uncomplicated hypertension, depression, history of bariatric surgery, ELIXSUM MEDIAN, long-term steroid, long-term NSAID,* and *long-term anticoagulation* were significantly associated with outcome of interest, and they were not included in the subsequent logistic regression. Schizophrenia was used as a replacement for psychosis. Result of chi-square analysis between acute pulmonary embolism and these significantly associated comorbid conditions following PSM are listed below:

**Acute PE:**
Psychoses: 0.23% vs 1.32%, p≤0.001

Schizophrenia: 0.49% vs 1.30%, p=0.004

History of Bariatric Surgery: 0.00% vs 1.31%, p≤0.001
Uncomplicated Hypertension: 1.80% vs 0.59%, ≤0.001

Depression: 0.14% vs 1.52%, p≤0.001

Long-term Anticoagulation: 2.72% vs 1.06%, p≤0.001

Long-term Steroid: 0.00% vs 1.33%, p≤0.001

Long-term NSAID: 0.00% vs 1.34%, p≤0.001

Elixhauser Sum ≥4: 1.90% vs 0.37%, p≤0.001

| **Supplementary Table 1: Covid-19 Demographics Table** | | | | | |  |  |
| --- | --- | --- | --- | --- | --- | --- | --- |
|  | **Covid-19 Disease** | **No Covid-19 Disease** |  | **Covid-19 Disease** | **No Covid-19 Disease** |  |  |
| **Frequency** | **1,698,559** | **25,968,827** | **ELIXHAUSER COMORBIDITIES** | **Mean Percent (Standard Error)** | **Mean Percent (Standard Error)** |  |  |
|  | | | **Uncomplicated Hypertension** | **54.99% (0.17) **** | **49.94% (0.18) **** |  |  |
|  |  |  | **Complicated Hypertension** | **27.00% (0.16) **** | **25.67% (0.14) **** |  |  |
|  |  |  | **Rheumatoid Arthritis/Collagen Vascular disorders** | **2.93% (0.04) **** | **3.12% (0.02) **** |  |  |
|  |  |  | **PUD (Peptic Ulcer Disease) w/o Bleeding** | **0.32% (0.01) **** | **0.70% (0.007) **** |  |  |
|  |  |  | **Paralysis** | **1.33% (0.02) **** | **2.42% (0.02) **** |  |  |
|  |  |  | **Alcohol Abuse** | **2.66% (0.04) **** | **7.34% (0.07) **** |  |  |
|  |  |  | **PUD w/o Bleeding** | **0.32% (0.01) **** | **0.70% (0.007) **** |  |  |
|  |  |  | **Renal Failure** | **20.97% (0.13) **** | **18.42% (0.10) **** |  |  |
|  |  |  | **Congestive Heart Failure** | **17.56% (0.13) **** | **19.97% (0.11) **** |  |  |
| **Demographics** | **Mean Percent (Standard Error)** | **Mean Percent (Standard Error)** | **Cardiac Arrhythmias** | **24.60% (0.14) **** | **23.05% (0.14) **** |  |  |
|  |  |  | **Valvular Disease** | **3.97% (0.05) **** | **7.05% (0.07) **** |  |  |
| **AGE Group** |  |  | **Metastatic Cancer** | **1.12% (0.02) **** | **3.63% (0.07) **** |  |  |
| **AGE 18-29** | **4.94 (0.07) **** | **12.24 (0.11) **** | **Pulmonary Circulation Disorders** | **5.26% (1.27) **** | **5.28% (1.29) **** |  |  |
| **AGE 30-54** | **24.19 (0.16) **** | **27.73 (0.13) **** | **Chronic Pulmonary Disease** | **22.11% (0.14)** | **22.12% (0.11)** |  |  |
| **AGE 55-64** | **19.26 (0.08) **** | **16.97 (0.07) **** | **Obesity** | **25.52 (0.23) **** | **18.10 (0.14) **** |  |  |
| **AGE 65-79** | **31.89 (0.07) **** | **27.45 (0.11) **** | **Severe Obesity** | **13.51% (0.11) **** | **9.23% (0.06) **** |  |  |
| **AGE 80-85** | **8.45 (0.06) **** | **6.78 (0.04) **** | **Cannabis Use** | **0 .81% (0.02) **** | **3.33% (0.05) **** |  |  |
| **Age 86-120** | **11.26 (0.10) **** | **8.82 (0.07) **** |  |  |  |  |  |
| **Race/Ethnicity** | | | **Peripheral Vascular Disorders** | **4.77% (1.14) **** | **7.24% (1.75) **** |  |  |
| **White** | **50.75% (0.56) **** | **66.63% (0.45) **** | **Other Neurological Disorders** | **17.12% (0.13) **** | **12.93% (0.07) **** |  |  |
| **Black** | **19.24% (0.41) **** | **15.62% (0.31) **** | **Uncomplicated Diabetes** | **14.75% (0.10) **** | **10.33% (0.05) **** |  |  |
|  |  |  | **Complicated Diabetes** | **26.18% (0.14) **** | **18.22% (0.10) **** |  |  |
| **Hispanic** | **21.38% (0.50) **** | **11.27% (0.29) **** | **Liver Disease** | **5.45% (0.06) **** | **6.86% (0.05) **** |  |  |
| **Asian/Pacific Islander** | **3.28% (0.12) **** | **2.80% (0.10) **** | **Coagulopathy** | **12.11% (0.08**** | **7.24% (0.18) **) **** |  |  |
| **Native American** | **1.02% (0.09) **** | **0.71% (0.05) **** | **Solid Tumor Without Metastasis** | **2.41% (0.40) **** | **2.48% (0.59) **** |  |  |
| **Other Racial or Ethnic Group** | **4.33% (0.19) **** | **2.98% (0.12) **** | **Coronary Artery Disease** | **16.86% (0.13) **** | **19.02% (0.13) **** |  |  |
| **SEX** | | | **Weight Loss** | **7.83% (0.13) **** | **7.30% (0.09) **** |  |  |
| **MALE** | **52.11% (0.02) **** | **43.30% (0.14) **** |  |  |  |  |  |
| **FEMALE** | **47.89% (0.12) **** | **56.70% (0.14) **** | **Drug Abuse** | **2.58% (0.05) **** | **8.04% (0.10) **** |  |  |
| **PRIMARY EXPECTED PAYER** | | | **Depression** | **11.69% (0.12) **** | **15.38 (0.11) **** |  |  |
| **Medicare** | **50.59% (0.27) **** | **46.19% (0.02) **** | **Hypothyroidism** | **13.15% (0.10) **** | **12.85% (0.07) **** |  |  |
| **Medicaid** | **14.50% (0.24) **** | **19.29% (0.02)** ****** | **Psychoses** | **2.57% (0.05) **** | **3.52% (0.08) **** |  |  |
| **Private Insurance** | **26.49% (0.25) **** | **26.77% (0.02)** ****** | **Blood Loss Anemia** | **0.45% (0.01) **** | **0.84% (0.009) **** |  |  |
| **Self-pay** | **3.79% (0.13)** ****** | **4.29% (0.01)** ****** | **Deficiency Anemia** | **3.87% (0.05) **** | **4.87% (0.04) **** |  |  |
| **No charge** | **0.27% (0.05) **** | **0.36% (0.003)** ****** | **AIDS/HIV** | **0.27% (0.01) **** | **0.34% (0.01) **** |  |  |
| **Other** | **4.36% (0.12) **** | **3.10% (0.01) **** | **Lymphoma** | **0.81% (0.02) **** | **1.18% (0.03) **** |  |  |
| **LENGTH OF STAY** | | | **Long-term Steroid Use** | **1.73% (0.04) **** | **1.51% (0.03) **** |  |  |
| **Less than 7 Days** | **60.39 (0.17) **** | **79.84 (0.12) **** | **Long-term Anticoagulation** | **9.52% (0.10) **** | **10.11% (0.07) **** |  |  |
| **7-13 Days** | **24.50 (0.11) **** | **14.36 (0.07) **** | **Long-term ASA** | **14.39% (0.19)** | **14.46% (0.16)** |  |  |
| **14-20 Days** | **8.01 (0.06) **** | **3.46 (0.03) **** |  |  |  |  |  |
| **21-29 Days** | **3.99 (0.04) **** | **1.37 (0.02) **** |  |  |  |  |  |
| **30-45 Days** | **2.14 (0.03) **** | **0.62 (0.10) **** |  |  |  |  |  |
| **45-60 Days** | **0.62 (0.004) **** | **0.19 (0.004) **** | **Baricitinib Administration** | **0.17% (0.03) **** | **.002% (3.05e-06) **** |  |  |
| **61-780 Days** | **0.37 (0.01) **** | **0.16 (0.004) **** | **Remdesivir Administration** | **22.80% (0.30) **** | **0.01% (8.51e-06) **** |  |  |
| **TOTAL CHARGE in US Dollars** | | | **Elixhauser Sum≥ 4** | **50.36 (0.22) **** | **46.30 (0.20) **** |  |  |
|  |  |  | **Admission on the Weekend** | **21.20 (0.05) **** | **25.90 % (0.08) **** |  |  |
|  |  |  | **ADMISSION MONTH** | **Covid-19 Disease** | **No Covid-19 Disease** |  |  |
| **Less than $50k** | **54.90 (0.40) **** | **62.64% (0.37) **** | **January** | **0.21% (0.01) **** | **10.10% (0.02) **** |  |  |
| **$50000-99,999** | **23.50% (0.19) **** | **21.42% (0.15) **** | **February** | **0.16% (0.009) **** | **9.28% (0.02) **** |  |  |
|  |  |  | **March** | **4.70% (0.16) **** | **8.23% (0.01) **** |  |  |
| **$100k-249,999** | **15.17% (0.19) **** | **12.66% (0.17) **** | **April** | **11.31% (0.26) **** | **6.17% (0.02) **** |  |  |
|  |  |  | **May** | **6.35% (0.11) **** | **7.66% (0.01) **** |  |  |
| **$250k-499,999** | **4.66% (0.08) **** | **2.62% (0.07) **** | **June** | **6.05% (0.10) **** | **8.45% (0.01) **** |  |  |
|  |  |  | **July** | **11.14% (0.17) **** | **8.49% (0.02) **** |  |  |
|  |  |  | **August** | **7.56% (0.09) **** | **8.58% (0.01) **** |  |  |
| **$500k-1m** | **1.77% (.05) **** | **0.66% (0.03) **** |  |  |  |  |  |
|  |  |  |  |  |  |  |  |
| **ZIPINC_QRTL—MEDIAN INCOME FOR PATIENT ZIP CODE** | | | **November** | **18.23% (0.16) **** | **7.78% (0.01) **** |  |  |
| **1 - $1-24,999** | **33.95% (0.56) **** | **30.43% (0.46) **** | **December** | **19.79% (0.17) **** | **7.94% (0.02) **** |  |  |
| **2- $25,000-34,999** | **27.11% (0.38) **** | **27.27% (0.33) **** |  |  |  |  |  |
| **3- $35,000-44,999** | **22.19% (0.35) **** | **22.84% (0.30) **** |  |  |  |  |  |
| **4- $45,000 or more** | **16.75% (0.47) **** | **19.46% (0.47) **** |  | | |  |  |
| **HOSPITAL REGION** | | |  |  |  |  |  |
| **Northeast** | **19.00% (0.53) **** | **18.15% (0.36) **** |  |  |  |  |  |
| **Midwest** | **22.19% (0.46) **** | **21.89% (0.40) **** |  |  |  |  |  |
| **South** | **40.72% (0.57) **** | **40.34% (0.44) **** |  |  |  |  |  |
| **West** | **18.09% (0.45) **** | **19.63% (0.35) **** |  |  |  |  |  |
| **BED-SIZE OF HOSPITAL** | | |  |  |  |  |  |
| **Small** | **24.27% (0.51) **** | **22.55% (0.37) **** |  |  |  |  |  |
| **Medium** | **29.02% (0.50) **** | **28.06% (0.35) **** |  |  |  |  |  |
|  |  |  |  |  |  |  |  |
| **Large** | **46.71% (0.59) **** | **49.38% (0.46) **** |  |  |  |  |  |
| **LOCATION & TEACHING STATUS OF HOSPITAL** | | | **TABLE LEGEND:** | | |  |  |
| **Rural** | **9.66% (0.24) **** | **8.64% (0.22) **** |  |  |  |  |  |
| **Urban Nonteaching** | **18.50% (0.40) **** | **17.87% (0.30) **** | ****: P less than or equal to 0.05 on χ2 analysis** | | |  |  |
| **Urban Teaching** | **71.84% (0.46) **** | **73.49% (0.36) **** |  |  |  |  |  |

**Supplementary TABLE 2**

|  | **No Covid-19 Disease**  **(N= 25,968,827**) | | | | | |
| --- | --- | --- | --- | --- | --- | --- |
| **Mean Length of Stay in Days (SD)** | **4.89 (0.02) **** | | | | | |
| **Mean Total Charge in US Dollars (SD)** | **$65,019.66 (834.69) **** | | | | | |
| **Mean Percent Mechanical Ventilation (SD)** | **3.69% (0.04) **** | | | | | |
| **Mean Percent AKI (SD)** | **24.18% (0.12) **** | | | | | |
| **Mean Percent Acute Pulmonary (SD)** | **1.22% (0.03) **** | | | | | |
| **Mean Percent Cardiogenic Shock (SD)** | **0.70% (0.01) **** | | | | | |
| **Mean Percent Septic Shock (SD)** | **2.25% (0.02) **** | | | | | |
| **Mean Percent: Mortality (SD)** | **2.53% (0.02) **** | | | | | |
| **Covid-19 Disease Subpopulation** | | | | | | |
|  | **Mean Percent (Standard Error)** | **Mean Percent (Standard Error)** | **Mean Percent (Standard Error)** | **Mean Percent (Standard Error)** | **Mean Percent (Standard Error)** | **Mean Percent (Standard Error)** |
|  | **N-MV P-Remdesivir** | **MV P-Remdesivir**  **(N= 43,290)** | **MV C-Remdesivir**  **(N= 4,305)** | **MV Both C- and P- Remdesivir**  **(N= 445)** | **N-MV No Remdesivir**  **(N= 1,154,635)** | **MV No Remdesivir**  **(N= 156,610)** |
| **Mean Length of Stay in Days (SD)** | **7.36 (0.04) **** | **18.94 (0.19) **** | **20.29 (0.61) **** | **18.88 (1.21) **** | **6.34 (0.3) **** | **17.84 (0.15) **** |
| **Mean Total Charge in US Dollars (SD)** | **$72,499.64 (984.03) **** | **$324,540.3 (7,178.06) **** | **$402,550.1 (23,543) **** | **$365,125.2 (38,114.10) **** | **$58,069.56 (746.71) **** | **$299,712.3 (5690.37) **** |
| **Mean Percent New-onset Atrial Fibrillation** | **5.43% (0.10) **** | **8.86% (0.30) **** | **7.67(0.85) **** | **10.11% (3.2) **** | **4.72% (0.07) **** | **7.10% (0.17) **** |
| **Mean Percent Atrial Flutter** | **1.27% (0.04)** | **3.76 % (0.21)** | **2.90% (0.6)** | **2.25% (1.6)** | **1.23% (0.03)** | **3.58% (.01)** |
| **Mean Percent Cardiac Arrest/Ventricular Fibrillation/Tachycardia** | **0.67% (0.03) **** | **15.11% (0.43) **** | **13.36% (0.01) **** | **14.61% (3.89) **** | **0.88 % (0.03)** | **16.23% (0.31) **** |
| **Mean Percent Septic Shock** | **1.26% (0.05) **** | **41.52% (0.68) **** | **47.74 (1.90) **** | **50.56 % (5.01) **** | **2.16% (0.04) **** | **47.22% (0.47) **** |
| **Mean Percent Cardiogenic Shock** | **0.11 % (0.01) **** | **2.92 % (0.19) **** | **4.07% (0.68) **** | **---** | **0.20 % (0.01) **** | **4.13% (0.14) **** |
| **Mean Percent AKI** | **24.20 % (0.23) **** | **64.86 % (0.60) **** | **68.64% (1.66) **** | **67.42% (5.01) **** | **30.53% (0.19) **** | **70.13 % (0.35) **** |
| **Mean Percent**: Mortality | **6.40% (0.13) **** | **63.33 % (0.65) **** | **55.05% (1.88) **** | **55.06% (5.42) **** | **7.47% (0.1 ** 0)** | **57.35 ** % (0.47)** |
| ****: P less than or equal to .05 on χ2 analysis** | | | | | | |

**Supplementary TABLE 3**

| **Covid-19 vs All-Cause Hospitalizations: Matched Analysis** | | | | |
| --- | --- | --- | --- | --- |
|  | **Population Size (Mean)** | | **Population Size (Mean)** | |
| **Outcome**: | Covid-19 | | **Control** | |
|  | **Unmatched** | **Matched** | **Unmatched** | **Matched** |
| **Death** | **320,041 (0.13253) **** | **320,041 (0.13253) **** | **4,919,500 (0.0247) **** | **4,919,500 (0.0342) **** |
| **Acute Pulmonary Embolism** | **320,242 (0.02324) **** | **320,242 (0.02324) **** | **4,920,809(0.01222) **** | **4,920,809(0.01495) **** |
| **Mechanical Ventilation** | **320,242 (0.11488) **** | **320,242 (0.11488) **** | **4,920,809 (.03543) **** | **4,920,809 (0.05707) **** |
|  | | | | |
| **No Mechanical Ventilation** | **320,242 (0.90285) **** | **320,242 (0.90285) **** | **4,920,809 (0.97641) **** | **4,920,809 (0.95795) **** |
| **Mechanical Ventilation <24 hours** | **320,242 (0.03046) **** | **320,242 (0.03046) **** | **4,920,809 (0.01464) **** | **4,920,809 (0.02393) **** |
| **Mechanical Ventilation within 24-48 hours** | **320,242 (0.01181) **** | **320,242 (0.01181) **** | **4,920,809 (0.00282) **** | **4,920,809 (0.00494) **** |
| **Mechanical Ventilation within 2-5 days** | **320,242 (0.02814) **** | **320,242 (0.02814) **** | **4,920,809 (0.00363) **** | **4,920,809 (0.0063) **** |
| **Mechanical Ventilation within 6-13 days** | **320,242 (0.01963) **** | **320,242 (0.01963) **** | **4,920,809 (0.00185) **** | **4,920,809 (0.00434) **** |
| **Mechanical Ventilation within 14-730 days** | **320,242 (0.0071) **** | **320,242 (0.0071) **** | **4,920,809 (0.00065) **** | **4,920,809 (0.00255) **** |
| **Covid-19 vs Other Pneumonia Hospitalizations: Matched Analysis** | | | | |
|  | Covid-19 | | Pneumonia Subpopulation Control | |
|  | Unmatched | Matched | Unmatched | Matched |
| **Death** | **320,041 (0.13253)   **** | **320,041(0.13253) **** | **99,625(0.02926) **** | **99,625(0.04001) **** |
| **Acute Pulmonary Embolism** | **320,041(0.02324) **** | **320,041(0.02324) **** | **99,625 (0.00884) **** | **99,625(0.01008) **** |
| **Mechanical Ventilation** | **320,041(.11) **** | **320,041(0.11488) **** | **99,625 (0.03454) **** | **.07082 (0.07082) **** |
|  | | | | |
| **No Mechanical Ventilation** | **320,041(0.90285) **** | **320,041(0.90) **** | **99,625 (.97347) **** | **99,625 (0.9393) **** |
| **Mechanical Ventilation <24 hours** | **320,041 (0.03046) **** | **320,041 (0.03046) **** | **99,625 (0.01267) **** | **99,625 (0. 0.02572) **** |
| **Mechanical Ventilation within 24-48 hours** | **320,041(0.01181) **** | **320,041(0.01181) **** | **. 99,625(0.00369) **** | **99,625(0.006) **** |
| **Mechanical Ventilation within 2-5 days** | **320,041 (0.02814) **** | **320,041 (0.02814) **** | **99,625 (0.0066) **** | **99,625 (0. .01584) **** |
| **Mechanical Ventilation within 6-13 days** | **320,041 (0.01963) **** | **320,041 (0.01963) **** | **99,625 (0.00279) **** | **99,625 (0.00971) **** |
| **Mechanical Ventilation within 14-730 days** | **320,041 (0.0071) **** | **320,041 (0.0071) **** | **99,625 (0.00077) **** | **99,625 (0.00342) **** |
| ****: P less than or equal to .05 on χ2 analysis** | | | | |

**Supplement Figure 1: *All-cause 2020 Hospital Mortality Rate over Time***


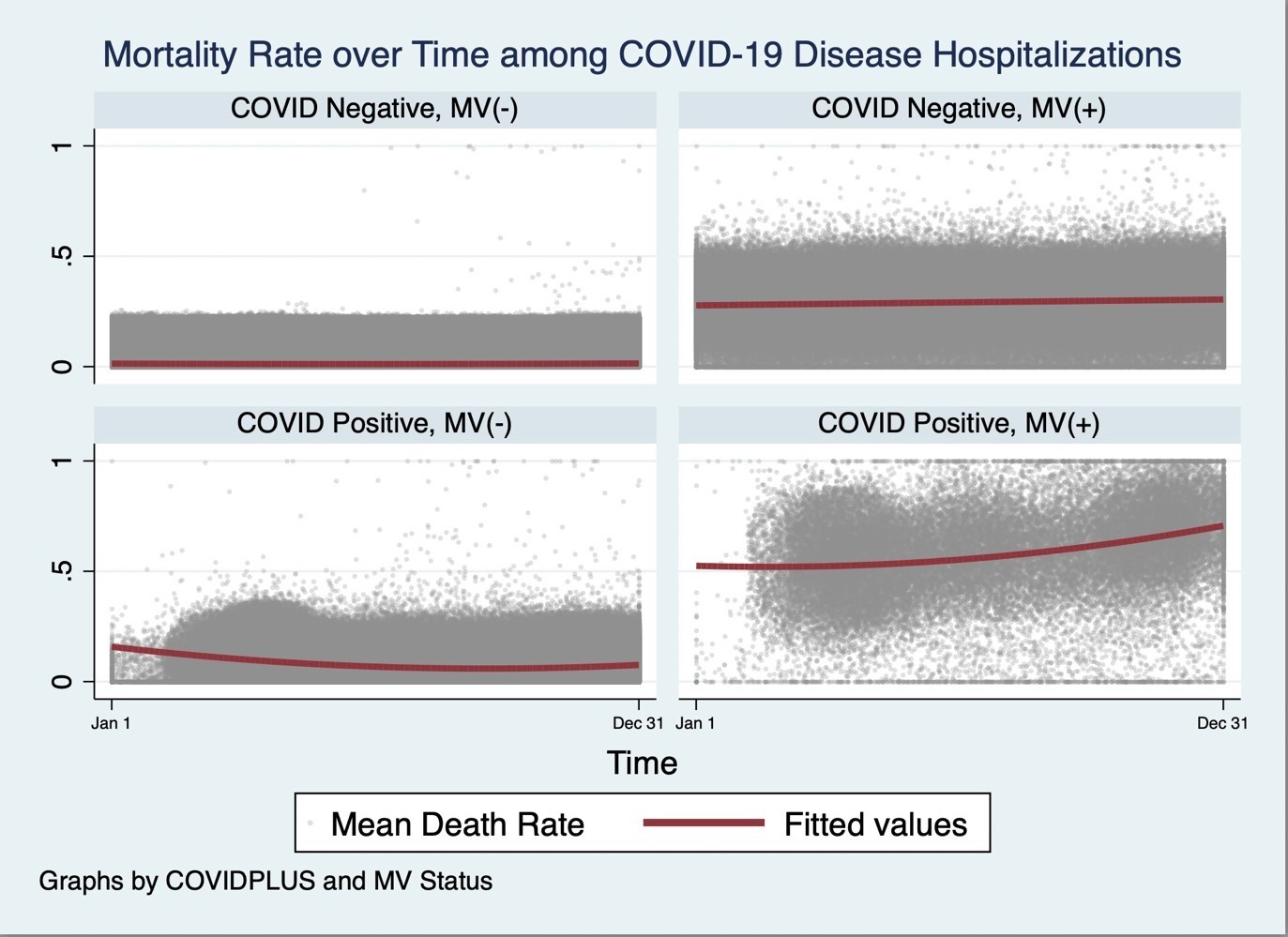


**Legend:**

**A:** The mortality rate over time of among all 2020 hospitalizations with and without Covid-19 by mechanical ventilation status.

**Supplement Figure 2: *Mortality Rate over Time among Covid-19***


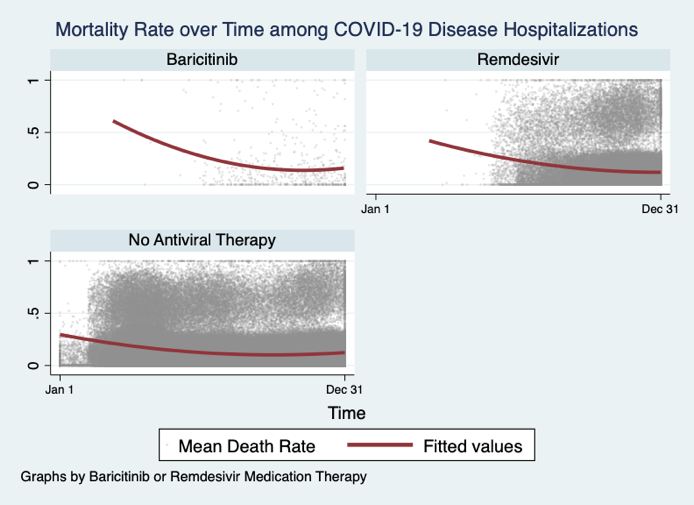


A.


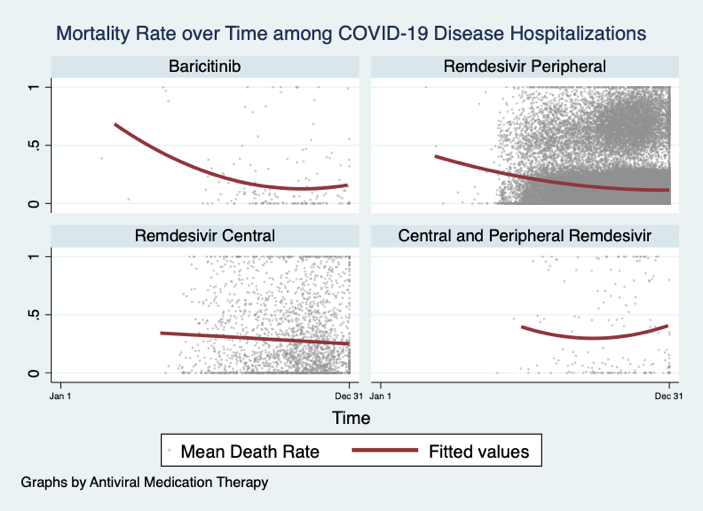


B.


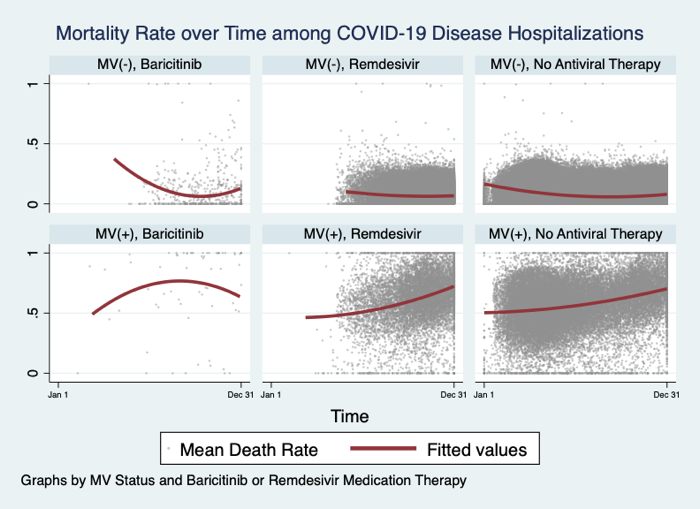
C.


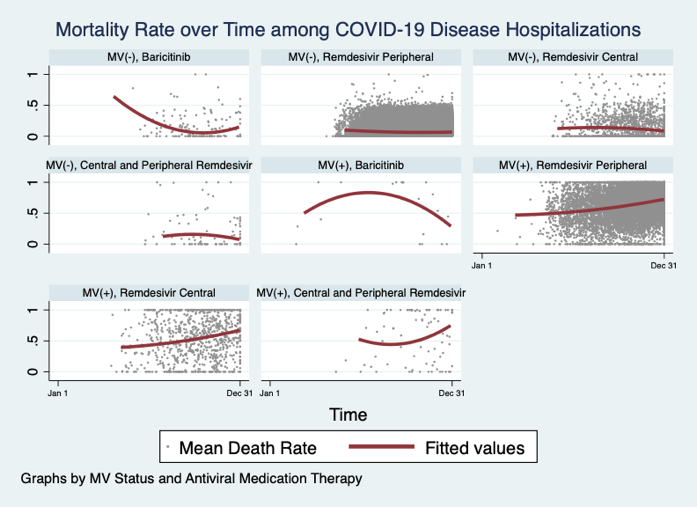
D.

**Legend:**

[Read top to bottom, left to right A-D]

**2A-D (Read Top to Bottom):** Mortality rate over time among Covid-19 hospitalizations/encounters by mechanical ventilation status and antiviral medication therapy received.

***Supplement Figure 3: Flow Diagram of Included Patient Population:***


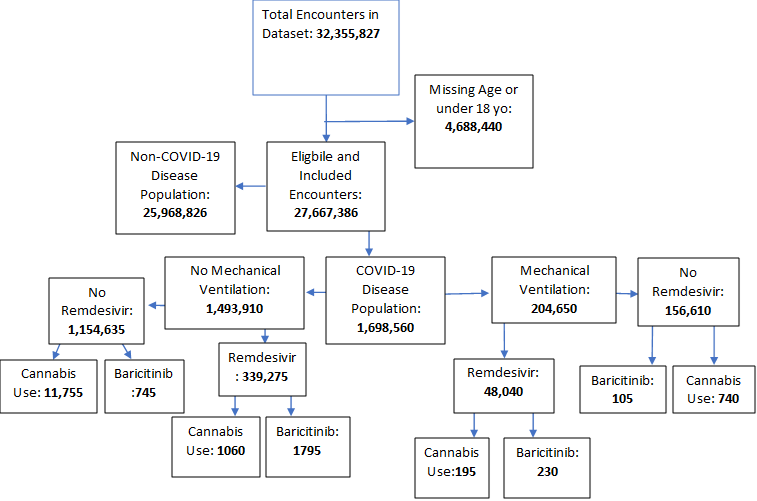


**Legend:** Flow diagram of included patient population **Supplementary TABLE 4**: **Logistic Regression for CBD among Covid-19 Hospitalizations**

| **Dependent Variable:**  **Active Cannabis Use** | Odds ratio | Linearized Std. Err. | t | P>\|t\| | [95% conf. interval] | |
| --- | --- | --- | --- | --- | --- | --- |
| Independent Variable | - | - | - | - | Lower bound | Upper Bound |
| Obesity | 0.5048877 | 0.0339736 | -10.16 | 0 | 0.4424886 | 0.5760863 |
| Diabetes Complicated | 0.5883996 | 0.0479143 | -6.51 | 0 | 0.501578 | 0.6902497 |
| \| |  |  |  |  |  |  |
| Obesity # Diabetes Complicated | 1.013405 | 0.1336734 | 0.1 | 0.92 | 0.7824812 | 1.312478 |
| \| |  |  |  |  |  |  |
| AGE GROUP (COMPARISON GROUP: 18-29 YEARS OLD) |  |  |  |  |  |  |
| AGE 30-54 | 0.2700122 | 0.0164992 | -21.43 | 0 | 0.2395279 | 0.3043762 |
| AGE 55-64 | 0.1126749 | 0.0089963 | -27.34 | 0 | 0.0963486 | 0.1317676 |
| AGE 65-79 | 0.0435544 | 0.0046839 | -29.14 | 0 | 0.035275 | 0.0537771 |
| AGE 80-85 | 0.0147814 | 0.0043149 | -14.44 | 0 | 0.0083401 | 0.0261977 |
| AGE 85-120 | 0.0029718 | 0.0015045 | -11.49 | 0 | 0.0011015 | 0.0080182 |
| \| |  |  |  |  |  |  |
| Racial or Ethnic Group (COMPARISON GROUP: White race) |  |  |  |  |  |  |
| Black | 1.720156 | 0.0958962 | 9.73 | 0 | 1.542061 | 1.91882 |
| Hispanic | 0.5401004 | 0.0393693 | -8.45 | 0 | 0.468178 | 0.6230717 |
| Asian/Pacific Islander | 0.4246469 | 0.079184 | -4.59 | 0 | 0.2946187 | 0.6120622 |
| Native American | 1.283615 | 0.2110853 | 1.52 | 0.129 | 0.9298638 | 1.771945 |
| Other Racial or Ethnic Group | 0.72181 | 0.09183 | -2.56 | 0.01 | 0.5624728 | 0.9262842 |
| \| |  |  |  |  |  |  |
| FEMALE (COMPARISON GROUP: MALE) | 0.5184218 | 0.0255709 | -13.32 | 0 | 0.4706376 | 0.5710576 |
| \| |  |  |  |  |  |  |
| PRIMARY PAYOR (COMPARISON GROUP: MEDICARE) |  |  |  |  |  |  |
| Medicaid | 1.299582 | 0.1024826 | 3.32 | 0.001 | 1.113425 | 1.516863 |
| Private Insurance | 0.7817054 | 0.0633855 | -3.04 | 0.002 | 0.6668118 | 0.9163954 |
| Self-pay | 1.394856 | 0.1318474 | 3.52 | 0 | 1.158906 | 1.678845 |
| No charge | 1.640787 | 0.4491978 | 1.81 | 0.071 | 0.9593001 | 2.806402 |
| Other | 1.131578 | 0.1269482 | 1.1 | 0.271 | 0.9081642 | 1.409953 |
| \| |  |  |  |  |  |  |
| HOSP-BEDSIZE (COMPARISON GROUP: SMALL) |  |  |  |  |  |  |
| Medium | 1.006917 | 0.0749291 | 0.09 | 0.926 | 0.8702309 | 1.165072 |
| Large | 0.9696423 | 0.0663469 | -0.45 | 0.652 | 0.8479158 | 1.108844 |
| \| |  |  |  |  |  |  |
| HOSP-LOCTEACH (COMPARISON GROUP: RURAL) |  |  |  |  |  |  |
| Urban Nonteaching | 1.637873 | 0.2061941 | 3.92 | 0 | 1.279651 | 2.096374 |
| Urban Teaching | 1.797384 | 0.2021849 | 5.21 | 0 | 1.441664 | 2.240875 |
| \| |  |  |  |  |  |  |
| HOSP-REGION (COMPARISON GROUP: WEST) |  |  |  |  |  |  |
| Northeast | 0.7248673 | 0.0655299 | -3.56 | 0 | 0.6071364 | 0.8654275 |
| Midwest | 0.7984816 | 0.0673156 | -2.67 | 0.008 | 0.6768382 | 0.9419871 |
| South | 0.6164475 | 0.0494932 | -6.03 | 0 | 0.5266671 | 0.7215328 |
| \| |  |  |  |  |  |  |
| ZIPINC-QRTL (COMPARISON GROUP: ZIPINC-QRTL 1) |  |  |  |  |  |  |
| 2 | 0.8894969 | 0.0527849 | -1.97 | 0.049 | 0.7918048 | 0.9992422 |
| 3 | 0.8254946 | 0.0552786 | -2.86 | 0.004 | 0.7239332 | 0.9413043 |
| 4 | 0.8344972 | 0.0650471 | -2.32 | 0.02 | 0.716238 | 0.9722824 |
| \| |  |  |  |  |  |  |
| ADMISSION ON THE WEEKEND | 1.041468 | 0.0507308 | 0.83 | 0.404 | 0.9466111 | 1.14583 |
| \| |  |  |  |  |  |  |
| LOS (COMPARISON GROUP: 0-6 DAYS) |  |  |  |  |  |  |
| 7-13 days | 0.873838 | 0.0623611 | -1.89 | 0.059 | 0.7597461 | 1.005063 |
| 14-20 days | 0.8737326 | 0.1072206 | -1.1 | 0.271 | 0.6869006 | 1.111381 |
| 21-29 days | 0.9620341 | 0.1441645 | -0.26 | 0.796 | 0.7171324 | 1.29057 |
| 30-45 days | 0.711795 | 0.1773529 | -1.36 | 0.173 | 0.4367254 | 1.160116 |
| 45-60 days | 1.451823 | 0.5028013 | 1.08 | 0.282 | 0.7362759 | 2.862773 |
| 61-780 days | 1.405812 | 0.5308728 | 0.9 | 0.367 | 0.670504 | 2.947495 |
| \| |  |  |  |  |  |  |
| TOTCHG (COMPARISON GROUP: $0-$50,000) |  |  |  |  |  |  |
| 50000-99,999 | 1.074568 | 0.0693961 | 1.11 | 0.265 | 0.9467772 | 1.219608 |
| 100k-249,999 | 1.158775 | 0.1051534 | 1.62 | 0.104 | 0.9699181 | 1.384404 |
| 250k-499,999 | 0.8136774 | 0.1403168 | -1.2 | 0.232 | 0.5802617 | 1.140987 |
| 500k-1m | 0.6752697 | 0.1776791 | -1.49 | 0.136 | 0.4031293 | 1.131124 |
| \| |  |  |  |  |  |  |
| ADMISSION MONTH (COMPARISON GROUP: JANUARY) |  |  |  |  |  |  |
| 2 | 1.795846 | 0.861688 | 1.22 | 0.222 | 0.7010194 | 4.600533 |
| 3 | 0.5620921 | 0.2107141 | -1.54 | 0.124 | 0.2695413 | 1.172168 |
| 4 | 0.5966325 | 0.2180159 | -1.41 | 0.158 | 0.2914625 | 1.221325 |
| 5 | 0.6993827 | 0.2604084 | -0.96 | 0.337 | 0.3370471 | 1.45124 |
| 6 | 0.8186596 | 0.3034094 | -0.54 | 0.589 | 0.3958641 | 1.693014 |
| 7 | 0.8800361 | 0.3212634 | -0.35 | 0.726 | 0.4302069 | 1.800212 |
| 8 | 0.9500115 | 0.3490818 | -0.14 | 0.889 | 0.4622407 | 1.952493 |
| 9 | 0.9402623 | 0.3457026 | -0.17 | 0.867 | 0.4573034 | 1.933275 |
| 10 | 0.8654899 | 0.3172825 | -0.39 | 0.694 | 0.4218239 | 1.775795 |
| 11 | 0.8631395 | 0.3129204 | -0.41 | 0.685 | 0.4240364 | 1.756948 |
| 12 | 0.84729 | 0.3066048 | -0.46 | 0.647 | 0.4167989 | 1.722414 |
| \| |  |  |  |  |  |  |
| ELIX SUM MEDIAN ≥4 | 6.006146 | 0.5383806 | 20 | 0 | 5.03819 | 7.16007 |
| Congestive Heart Failure | 1.202496 | 0.1201894 | 1.84 | 0.065 | 0.9885134 | 1.462799 |
| Cardiac Arrhythmias | 0.7165189 | 0.0512388 | -4.66 | 0 | 0.6227887 | 0.8243555 |
| Valvular Disease | 0.9131742 | 0.1417014 | -0.59 | 0.558 | 0.6736469 | 1.23787 |
| Pulmonary Circulation Disorders | 0.6945327 | 0.0865797 | -2.92 | 0.003 | 0.543943 | 0.8868127 |
| Peripheral Vascular Disorders | 0.8088754 | 0.1123937 | -1.53 | 0.127 | 0.6159904 | 1.062158 |
| Hypertension Uncomplicated | 0.6579386 | 0.0412086 | -6.68 | 0 | 0.581912 | 0.7438979 |
| Paralysis | 0.8047968 | 0.1348575 | -1.3 | 0.195 | 0.5794465 | 1.117787 |
| Other Neurological Disorders | 0.7504688 | 0.0587162 | -3.67 | 0 | 0.6437493 | 0.8748801 |
| Chronic Pulmonary Disease | 0.8327461 | 0.0490642 | -3.11 | 0.002 | 0.741903 | 0.9347126 |
| Diabetes Uncomplicated | 0.5020311 | 0.0406359 | -8.51 | 0 | 0.4283639 | 0.5883672 |
| Hypothyroidism | 0.4589419 | 0.0511408 | -6.99 | 0 | 0.368876 | 0.5709985 |
| Renal Failure | 0.7038732 | 0.0742875 | -3.33 | 0.001 | 0.5723121 | 0.8656772 |
| Liver Disease | 0.6831906 | 0.0637395 | -4.08 | 0 | 0.5689911 | 0.8203105 |
| Peptic Ulcer Disease without Bleeding | 1.553175 | 0.4532269 | 1.51 | 0.131 | 0.8765273 | 2.752172 |
| AIDS/HIV | 0.7253241 | 0.2178823 | -1.07 | 0.285 | 0.4025005 | 1.307067 |
| Metastatic Cancer | 0.8463839 | 0.2748608 | -0.51 | 0.608 | 0.4477809 | 1.599813 |
| Solid Tumor Without Metastasis | 0.8109324 | 0.1910165 | -0.89 | 0.374 | 0.5110074 | 1.286892 |
| Lymphoma | 0.713983 | 0.2279517 | -1.06 | 0.291 | 0.3818137 | 1.335132 |
| Rheumatoid Arthritis/Collagen Vascular | 0.7673977 | 0.1223699 | -1.66 | 0.097 | 0.5613705 | 1.049038 |
| Coagulopathy | 0.5587324 | 0.047733 | -6.81 | 0 | 0.4725685 | 0.6606066 |
| Weight Loss | 0.725979 | 0.0775979 | -3 | 0.003 | 0.5887302 | 0.8952241 |
| Fluid and Electrolyte Disorders | 0.6951124 | 0.0358902 | -7.04 | 0 | 0.6281935 | 0.76916 |
| Blood Loss Anemia | 0.4120275 | 0.171363 | -2.13 | 0.033 | 0.1823105 | 0.9311949 |
| Deficiency Anemia | 0.7264064 | 0.0853083 | -2.72 | 0.007 | 0.5770161 | 0.914474 |
| Alcohol Abuse | 1.779508 | 0.1437785 | 7.13 | 0 | 1.518821 | 2.084939 |
| Psychoses | 2.341964 | 0.1924853 | 10.35 | 0 | 1.99343 | 2.751437 |
| Depression | 1.319621 | 0.084469 | 4.33 | 0 | 1.163989 | 1.496063 |
| Hypertension Complicated | 0.6567651 | 0.0749472 | -3.68 | 0 | 0.5251069 | 0.8214335 |
| Aspirin | 0.8702898 | 0.0737239 | -1.64 | 0.101 | 0.737118 | 1.027521 |
| Long Term Anti Coagulation | 0.854699 | 0.0907948 | -1.48 | 0.139 | 0.6940086 | 1.052596 |
| Long Term NSAID | 1.675963 | 0.3212931 | 2.69 | 0.007 | 1.150902 | 2.440565 |
| Long Term Steroid | 0.8490077 | 0.1614752 | -0.86 | 0.389 | 0.5847556 | 1.232676 |
| Tobacco Use Disorder | 4.189618 | 0.2327576 | 25.79 | 0 | 3.757268 | 4.671719 |
| Coronary Artery Disease | 1.129459 | 0.1032527 | 1.33 | 0.183 | 0.9441343 | 1.35116 |
| Hypomagnesemia | 1.169152 | 0.1316209 | 1.39 | 0.165 | 0.9376005 | 1.457888 |
| Hyperhomocysteinemia | 8.612445 | 7.113998 | 2.61 | 0.009 | 1.705406 | 43.49359 |
| Vitamin D Deficiency | 1.08816 | 0.1602616 | 0.57 | 0.566 | 0.8152583 | 1.452414 |
| History of Bariatric Surgery | 0.8959904 | 0.2551996 | -0.39 | 0.7 | 0.5126177 | 1.566077 |
| _cons | 0.0668545 | 0.0266951 | -6.77 | 0 | 0.0305598 | 0.1462549 |
| Note: P>\|t\|=0: p≤0.001  #: Interaction Term | | | | | | |

**Supplementary TABLE 5: Logistic Regression for Acute on Chronic Decompensated Systolic Heart Failure among Covid-19 Hospitalizations**

|  | Odds ratio | Linearized Std. Err. | t | P>\|t\| | [95% conf. interval] | |
| --- | --- | --- | --- | --- | --- | --- |
| **Dependent Variable: Acute Decompensated Systolic Heart Failure** |  |  |  |  |  | |
| Independent Variable | - | - | - | - | Lower bound | Upper Bound |
| ACU | 1.53272 | 0.2508849 | 2.61 | 0.009 | 1.111973 | 2.112668 |
| Obesity | 0.7457592 | 0.0377436 | -5.8 | 0 | 0.6753153 | 0.8235514 |
| Diabetes Complicated | 0.8579358 | 0.0325751 | -4.04 | 0 | 0.7963911 | 0.9242366 |
| \| |  |  |  |  |  |  |
| Obesity # Diabetes Complicated | 0.9720869 | 0.0678515 | -0.41 | 0.685 | 0.8477639 | 1.114642 |
| \| |  |  |  |  |  |  |
| AGE GROUP (COMPARISON GROUP: 18-29 YEARS OLD) |  |  |  |  |  |  |
| AGE 30-54 | 0.454637 | 0.0725504 | -4.94 | 0 | 0.3325013 | 0.6216359 |
| AGE 55-64 | 0.3413252 | 0.0543628 | -6.75 | 0 | 0.2497814 | 0.4664194 |
| AGE 65-79 | 0.3195971 | 0.0505404 | -7.21 | 0 | 0.2344005 | 0.4357598 |
| AGE 80-85 | 0.2759759 | 0.0455132 | -7.81 | 0 | 0.1997352 | 0.3813184 |
| AGE 85-120 | 0.2660805 | 0.0439499 | -8.02 | 0 | 0.1924761 | 0.3678319 |
| \| |  |  |  |  |  |  |
| Racial or Ethnic Group (COMPARISON GROUP: White race) |  |  |  |  |  |  |
| Black | 1.030822 | 0.0441941 | 0.71 | 0.479 | 0.9477209 | 1.12121 |
| Hispanic | 0.9513598 | 0.0484791 | -0.98 | 0.328 | 0.8609095 | 1.051313 |
| Asian/Pacific Islander | 0.7575626 | 0.0801072 | -2.63 | 0.009 | 0.6157221 | 0.9320781 |
| Native American | 0.8290263 | 0.1719953 | -0.9 | 0.366 | 0.5519802 | 1.245125 |
| Other Racial or Ethnic Group | 1.158298 | 0.0968459 | 1.76 | 0.079 | 0.9831758 | 1.364613 |
| \| |  |  |  |  |  |  |
| FEMALE (COMPARISON GROUP: MALE) | 0.6775976 | 0.0232388 | -11.35 | 0 | 0.6335357 | 0.7247241 |
| \| |  |  |  |  |  |  |
| PRIMARY PAYOR (COMPARISON GROUP: MEDICARE) |  |  |  |  |  |  |
| Medicaid | 1.36138 | 0.0772773 | 5.43 | 0 | 1.218004 | 1.521635 |
| Private Insurance | 1.074395 | 0.0531428 | 1.45 | 0.147 | 0.9751001 | 1.1838 |
| Self-pay | 1.70785 | 0.1689205 | 5.41 | 0 | 1.40681 | 2.073308 |
| No charge | 2.09043 | 0.6050532 | 2.55 | 0.011 | 1.185212 | 3.687018 |
| Other | 1.149647 | 0.1021631 | 1.57 | 0.117 | 0.9658319 | 1.368444 |
| \| |  |  |  |  |  |  |
| HOSP-BEDSIZE (COMPARISON GROUP: SMALL) |  |  |  |  |  |  |
| Medium | 1.012788 | 0.0501041 | 0.26 | 0.797 | 0.919172 | 1.115939 |
| Large | 1.075055 | 0.0497619 | 1.56 | 0.118 | 0.9817917 | 1.177177 |
| \| |  |  |  |  |  |  |
| HOSP-LOCTEACH (COMPARISON GROUP: RURAL) |  |  |  |  |  |  |
| Urban Nonteaching | 1.069305 | 0.0789854 | 0.91 | 0.364 | 0.9251438 | 1.23593 |
| Urban Teaching | 1.149525 | 0.0748564 | 2.14 | 0.032 | 1.01175 | 1.306061 |
| \| |  |  |  |  |  |  |
| HOSP-REGION (COMPARISON GROUP: NORTHEAST) |  |  |  |  |  |  |
| Midwest | 0.9283129 | 0.0557962 | -1.24 | 0.216 | 0.8251233 | 1.044407 |
| South | 1.07589 | 0.0609329 | 1.29 | 0.197 | 0.9628241 | 1.202234 |
| West | 1.259636 | 0.0799097 | 3.64 | 0 | 1.112323 | 1.426458 |
| \| |  |  |  |  |  |  |
| ZIPINC-QRTL (COMPARISON GROUP: ZIPINC-QRTL 1) |  |  |  |  |  |  |
| 2 | 0.8864929 | 0.0356437 | -3 | 0.003 | 0.8192964 | 0.9592006 |
| 3 | 0.840981 | 0.0373917 | -3.9 | 0 | 0.7707784 | 0.9175776 |
| 4 | 0.883107 | 0.0457869 | -2.4 | 0.017 | 0.797753 | 0.9775934 |
| \| |  |  |  |  |  |  |
| ADMISSION ON THE WEEKEND | 1.000062 | 0.0346934 | 0 | 0.999 | 0.9343065 | 1.070445 |
| \| |  |  |  |  |  |  |
| LOS (COMPARISON GROUP: 0-6 DAYS) |  |  |  |  |  |  |
| 7-13 days | 1.099882 | 0.0484987 | 2.16 | 0.031 | 1.008793 | 1.199194 |
| 14-20 days | 1.156122 | 0.0724454 | 2.32 | 0.021 | 1.02247 | 1.307244 |
| 21-29 days | 1.066473 | 0.0860861 | 0.8 | 0.425 | 0.9103778 | 1.249333 |
| 30-45 days | 0.9785878 | 0.1055158 | -0.2 | 0.841 | 0.7921253 | 1.208943 |
| 45-60 days | 1.057651 | 0.2153741 | 0.28 | 0.783 | 0.7095135 | 1.57661 |
| 61-780 days | 0.9005888 | 0.2784093 | -0.34 | 0.735 | 0.4912607 | 1.650977 |
| \| |  |  |  |  |  |  |
| TOTCHG (COMPARISON GROUP: $0-$50,000) |  |  |  |  |  |  |
| 50000-99,999 | 1.295156 | 0.0567256 | 5.91 | 0 | 1.188586 | 1.411281 |
| 100k-249,999 | 1.530354 | 0.0855386 | 7.61 | 0 | 1.371517 | 1.707587 |
| 250k-499,999 | 1.714623 | 0.153482 | 6.02 | 0 | 1.438645 | 2.043543 |
| 500k-1m | 2.155044 | 0.2697633 | 6.13 | 0 | 1.686069 | 2.754464 |
| \| |  |  |  |  |  |  |
| ADMISSION MONTH (COMPARISON GROUP: JANUARY) |  |  |  |  |  |  |
| 2 | 0.7691577 | 0.2687825 | -0.75 | 0.453 | 0.3876861 | 1.525986 |
| 3 | 0.4365573 | 0.1004323 | -3.6 | 0 | 0.2780761 | 0.6853603 |
| 4 | 0.4360806 | 0.0957242 | -3.78 | 0 | 0.2835747 | 0.6706038 |
| 5 | 0.5370542 | 0.1188833 | -2.81 | 0.005 | 0.3479706 | 0.828884 |
| 6 | 0.6260751 | 0.1418806 | -2.07 | 0.039 | 0.4014898 | 0.9762891 |
| 7 | 0.5432724 | 0.1205771 | -2.75 | 0.006 | 0.3515966 | 0.8394419 |
| 8 | 0.6050173 | 0.134814 | -2.26 | 0.024 | 0.3908813 | 0.9364632 |
| 9 | 0.634084 | 0.1409281 | -2.05 | 0.04 | 0.41012 | 0.9803535 |
| 10 | 0.5967996 | 0.1328792 | -2.32 | 0.02 | 0.3857034 | 0.923429 |
| 11 | 0.6424226 | 0.1407919 | -2.02 | 0.044 | 0.4180437 | 0.9872335 |
| 12 | 0.5977507 | 0.1306 | -2.36 | 0.019 | 0.3894872 | 0.9173752 |
| \| |  |  |  |  |  |  |
| ELIX SUM MEDIAN ≥4 | 1.613104 | 0.1136115 | 6.79 | 0 | 1.405061 | 1.85195 |
| Cardiac Arrhythmias | 1.591454 | 0.0575211 | 12.86 | 0 | 1.482586 | 1.708316 |
| Valvular Disease | 1.525316 | 0.067991 | 9.47 | 0 | 1.397678 | 1.664611 |
| Pulmonary Circulation Disorders | 1.274849 | 0.0589341 | 5.25 | 0 | 1.16439 | 1.395788 |
| Peripheral Vascular Disorders | 1.545491 | 0.0683372 | 9.85 | 0 | 1.417158 | 1.685445 |
| Hypertension Uncomplicated | 0.7784419 | 0.0969673 | -2.01 | 0.044 | 0.6097702 | 0.9937706 |
| Paralysis | 0.8320855 | 0.0332013 | -4.61 | 0 | 0.769475 | 0.8997905 |
| Other Neurological Disorders | 0.811349 | 0.0279537 | -6.07 | 0 | 0.7583554 | 0.8680457 |
| Chronic Pulmonary Disease | 0.8570608 | 0.0426836 | -3.1 | 0.002 | 0.7773349 | 0.9449637 |
| Diabetes Uncomplicated | 0.9274209 | 0.0404099 | -1.73 | 0.084 | 0.8514865 | 1.010127 |
| Hypothyroidism | 0.941181 | 0.0389729 | -1.46 | 0.143 | 0.8677935 | 1.020775 |
| Renal Failure | 1.244604 | 0.0720812 | 3.78 | 0 | 1.111016 | 1.394255 |
| Liver Disease | 0.8859577 | 0.2284222 | -0.47 | 0.639 | 0.5344303 | 1.468706 |
| Peptic Ulcer Disease without Bleeding | 1.103206 | 0.3071288 | 0.35 | 0.724 | 0.6391744 | 1.904118 |
| AIDSHIV | 0.9808145 | 0.1518954 | -0.13 | 0.9 | 0.7239819 | 1.328758 |
| Metastatic Cancer | 0.9768295 | 0.0946159 | -0.24 | 0.809 | 0.8078828 | 1.181107 |
| Solid Tumor Without Metastasis | 0.9414595 | 0.147887 | -0.38 | 0.701 | 0.6919195 | 1.280996 |
| Lymphoma | 0.9204296 | 0.0831416 | -0.92 | 0.359 | 0.7710472 | 1.098753 |
| Rheumatoid Arthritis/Collagen Vascular | 0.9458127 | 0.0393472 | -1.34 | 0.181 | 0.8717343 | 1.026186 |
| Coagulopathy | 0.9557901 | 0.0498148 | -0.87 | 0.386 | 0.8629518 | 1.058616 |
| Weight Loss | 0.9031829 | 0.0304456 | -3.02 | 0.003 | 0.8454237 | 0.9648881 |
| Fluid and Electrolyte Disorders | 0.578742 | 0.1349638 | -2.35 | 0.019 | 0.3663769 | 0.9142013 |
| Blood Loss Anemia | 1.069489 | 0.070067 | 1.03 | 0.305 | 0.9405782 | 1.216068 |
| Deficiency Anemia | 0.9387617 | 0.0852174 | -0.7 | 0.486 | 0.7857148 | 1.12162 |
| Alcohol Abuse | 0.776167 | 0.0855486 | -2.3 | 0.022 | 0.6253316 | 0.9633853 |
| Psychoses | 0.716091 | 0.0354568 | -6.74 | 0 | 0.6498451 | 0.78909 |
| Depression | 10.28633 | 0.6106255 | 39.26 | 0 | 9.156227 | 11.55591 |
| Hypertension Complicated | 1.033969 | 0.0390504 | 0.88 | 0.376 | 0.9601762 | 1.113433 |
| Aspirin | 0.9365444 | 0.0379948 | -1.62 | 0.106 | 0.8649406 | 1.014076 |
| Long Term Anti Coagulation | 0.7395739 | 0.1576995 | -1.41 | 0.157 | 0.4868884 | 1.123398 |
| Long Term NSAID | 0.6940539 | 0.0919988 | -2.76 | 0.006 | 0.5352206 | 0.900023 |
| Long Term Steroid | 1.325591 | 0.0883256 | 4.23 | 0 | 1.163262 | 1.510573 |
| Tobacco Use Disorder | 1.429888 | 0.0505734 | 10.11 | 0 | 1.334098 | 1.532556 |
| Coronary Artery Disease | 1.215048 | 0.0823001 | 2.88 | 0.004 | 1.063952 | 1.387601 |
| Hypomagnesemia | 2.838208 | 2.785784 | 1.06 | 0.288 | 0.4143181 | 19.44261 |
| Hyperhomocysteinemia | 0.3961625 | 0.2458847 | -1.49 | 0.136 | 0.1173316 | 1.337616 |
| Vitamin D Deficiency | 0.8655547 | 0.1016163 | -1.23 | 0.219 | 0.6875997 | 1.089566 |
| History of Bariatric Surgery | 0.8005325 | 0.1869147 | -0.95 | 0.341 | 0.506499 | 1.265259 |
| AKI | 1.148702 | 0.0458594 | 3.47 | 0.001 | 1.062223 | 1.242222 |
| Remdesivir | 0.8410342 | 0.0348685 | -4.18 | 0 | 0.7753788 | 0.9122491 |
| Baricitinib | 0.4946438 | 0.2149722 | -1.62 | 0.105 | 0.2109881 | 1.15965 |
| \| |  |  |  |  |  |  |
| Mechanical Ventilation Day (Comparison group: ≤24 hours status post admission) |  |  |  |  |  |  |
| 24-48hr | 0.7763068 | 0.0883782 | -2.22 | 0.026 | 0.6210149 | 0.9704311 |
| 2-5 days | 0.6856646 | 0.0632057 | -4.09 | 0 | 0.5723015 | 0.821483 |
| 6-13 days | 0.4736359 | 0.0544007 | -6.51 | 0 | 0.3781382 | 0.5932511 |
| 14-730 days | 0.6649687 | 0.0941118 | -2.88 | 0.004 | 0.5038473 | 0.8776139 |
| No Mechanical Ventilation | 0.6292716 | 0.0458556 | -6.36 | 0 | 0.545498 | 0.7259106 |
| \| |  |  |  |  |  |  |
| Vasopressor Use | 1.16691 | 0.098944 | 1.82 | 0.069 | 0.9881953 | 1.377945 |
| Septic Shock | 1.204324 | 0.0731747 | 3.06 | 0.002 | 1.06908 | 1.356678 |
| _cons | 0.0299074 | 0.0087569 | -11.99 | 0 | 0.0168452 | 0.0530983 |
| Note: P>\|t\|=0: p≤0.001  #: Interaction Term | | | | | | |

**Supplementary TABLE 6: Logistic Regression for Outcome Death among Covid-19 Hospitalizations**

| Dependent Variable: Death | Odds ratio | Linearized Std. Err. | t | P>\|t\| | [95% conf. interval] | |
| --- | --- | --- | --- | --- | --- | --- |
| Independent Variable | - | - | - | - | Lower bound | Upper Bound |
| ACU | 0.4693525 | 0.0649689 | -5.46 | 0 | 0.3578007 | 0.6156829 |
| Obesity | 0.867904 | 0.0205908 | -5.97 | 0 | 0.8284601 | 0.909226 |
| Diabetes Complicated | 0.9908986 | 0.0185803 | -0.49 | 0.626 | 0.9551332 | 1.028003 |
| \| | - | - | - | - | - | - |
| Obesity # Diabetes Complicated | 1.182083 | 0.0405135 | 4.88 | 0 | 1.105266 | 1.264239 |
| \| | - | - | - | - | - | - |
| AGE GROUP (COMPARISON GROUP: 18-29 YEARS OLD) | - | - | - | - | - | - |
| AGE 30-54 | 2.053394 | 0.1433786 | 10.3 | 0 | 1.79069 | 2.354637 |
| AGE 55-64 | 4.184006 | 0.2962949 | 20.21 | 0 | 3.641638 | 4.80715 |
| AGE 65-79 | 9.555421 | 0.7051386 | 30.59 | 0 | 8.26834 | 11.04285 |
| AGE 80-85 | 18.57502 | 1.420751 | 38.2 | 0 | 15.98841 | 21.5801 |
| AGE 85-120 | 29.98449 | 2.297841 | 44.38 | 0 | 25.80165 | 34.84544 |
| \| | - | - | - | - | - | - |
| Racial or Ethnic Group (COMPARISON GROUP: White race) | - | - | - | - | - | - |
| Black | 0.7854504 | 0.0197069 | -9.63 | 0 | 0.7477496 | 0.8250519 |
| Hispanic | 1.024796 | 0.0300218 | 0.84 | 0.403 | 0.9675963 | 1.085377 |
| Asian/Pacific Islander | 0.8812911 | 0.0405396 | -2.75 | 0.006 | 0.8052915 | 0.9644632 |
| Native American | 1.397136 | 0.1220634 | 3.83 | 0 | 1.177203 | 1.658159 |
| Other Racial or Ethnic Group | 0.9759694 | 0.0449668 | -0.53 | 0.598 | 0.891676 | 1.068231 |
| \| | - | - | - | - | - | - |
| FEMALE (COMPARISON GROUP: MALE) | 0.9057605 | 0.0131475 | -6.82 | 0 | 0.8803481 | 0.9319065 |
| \| | - | - | - | - | - | - |
| PRIMARY PAYOR (COMPARISON GROUP: MEDICARE) | - | - | - | - | - | - |
| Medicaid | 1.152776 | 0.0377975 | 4.34 | 0 | 1.081006 | 1.229312 |
| Private Insurance | 1.077401 | 0.0359144 | 2.24 | 0.025 | 1.009242 | 1.150163 |
| Self-pay | 1.474 | 0.0976619 | 5.86 | 0 | 1.294447 | 1.678459 |
| No charge | 1.066277 | 0.1732657 | 0.39 | 0.693 | 0.7753805 | 1.466308 |
| Other | 2.160306 | 0.1293583 | 12.86 | 0 | 1.921018 | 2.429399 |
| \| | - | - | - | - | - | - |
| HOSP-BEDSIZE (COMPARISON GROUP: SMALL) | - | - | - | - | - | - |
| Medium | 1.105282 | 0.0379955 | 2.91 | 0.004 | 1.033246 | 1.18234 |
| Large | 1.035723 | 0.0350314 | 1.04 | 0.299 | 0.9692712 | 1.10673 |
| \| | - | - | - | - | - | - |
| HOSP-LOCTEACH (COMPARISON GROUP: RURAL) | - | - | - | - | - | - |
| Urban Nonteaching | 1.03723 | 0.0453428 | 0.84 | 0.403 | 0.9520383 | 1.130046 |
| Urban Teaching | 1.050146 | 0.0417798 | 1.23 | 0.219 | 0.9713498 | 1.135335 |
| \| | - | - | - | - | - | - |
| HOSP-REGION (COMPARISON GROUP: NORTHEAST) | - | - | - | - | - | - |
| Midwest | 0.7583341 | 0.0335559 | -6.25 | 0 | 0.6953204 | 0.8270585 |
| South | 0.8752914 | 0.0355681 | -3.28 | 0.001 | 0.8082651 | 0.9478758 |
| West | 0.875054 | 0.0397642 | -2.94 | 0.003 | 0.8004677 | 0.9565901 |
| \| | - | - | - | - | - | - |
| ZIPINC-QRTL (COMPARISON GROUP: ZIPINC-QRTL 1) | - | - | - | - | - | - |
| 2 | 0.8933689 | 0.0207448 | -4.86 | 0 | 0.8536105 | 0.9349791 |
| 3 | 0.8310277 | 0.0212611 | -7.23 | 0 | 0.7903732 | 0.8737733 |
| 4 | 0.780732 | 0.0247165 | -7.82 | 0 | 0.7337482 | 0.8307243 |
| \| | - | - | - | - | - | - |
| ADMISSION ON THE WEEKEND | 1.005674 | 0.0158028 | 0.36 | 0.719 | 0.9751644 | 1.037137 |
| \| | - | - | - | - | - | - |
| LOS (COMPARISON GROUP: 0-6 DAYS) | - | - | - | - | - | - |
| 7-13 days | 0.7930348 | 0.0182295 | -10.09 | 0 | 0.758089 | 0.8295915 |
| 14-20 days | 0.5872914 | 0.0218129 | -14.33 | 0 | 0.5460469 | 0.6316513 |
| 21-29 days | 0.2977283 | 0.0156078 | -23.11 | 0 | 0.268649 | 0.3299552 |
| 30-45 days | 0.1118278 | 0.0081482 | -30.07 | 0 | 0.0969417 | 0.1289997 |
| 45-60 days | 0.0529835 | 0.0067791 | -22.96 | 0 | 0.0412289 | 0.0680895 |
| 61-780 days | 0.0319702 | 0.0070735 | -15.56 | 0 | 0.0207188 | 0.0493319 |
| \| | - | - | - | - | - | - |
| TOTCHG (COMPARISON GROUP: $0-$50,000) | - | - | - | - | - | - |
| 50000-99,999 | 1.111934 | 0.0271222 | 4.35 | 0 | 1.060012 | 1.166399 |
| 100k-249,999 | 1.708242 | 0.0670213 | 13.65 | 0 | 1.581772 | 1.844823 |
| 250k-499,999 | 2.447179 | 0.1593996 | 13.74 | 0 | 2.153806 | 2.780514 |
| 500k-1m | 3.355266 | 0.3109139 | 13.06 | 0 | 2.79788 | 4.023694 |
| \| | - | - | - | - | - | - |
| ADMISSION MONTH (COMPARISON GROUP: JANUARY) | - | - | - | - | - | - |
| 2 | 1.540593 | 0.5129629 | 1.3 | 0.194 | 0.8020279 | 2.959282 |
| 3 | 6.188205 | 1.534748 | 7.35 | 0 | 3.805381 | 10.06309 |
| 4 | 7.132837 | 1.75417 | 7.99 | 0 | 4.404224 | 11.55195 |
| 5 | 4.587864 | 1.127771 | 6.2 | 0 | 2.833437 | 7.428609 |
| 6 | 4.238658 | 1.040575 | 5.88 | 0 | 2.619411 | 6.858879 |
| 7 | 4.725544 | 1.158886 | 6.33 | 0 | 2.921773 | 7.642881 |
| 8 | 4.14045 | 1.01525 | 5.79 | 0 | 2.560194 | 6.696105 |
| 9 | 4.190959 | 1.033847 | 5.81 | 0 | 2.583905 | 6.797518 |
| 10 | 4.386523 | 1.080464 | 6 | 0 | 2.706445 | 7.109542 |
| 11 | 4.576401 | 1.118749 | 6.22 | 0 | 2.833879 | 7.39038 |
| 12 | 4.779948 | 1.167724 | 6.4 | 0 | 2.960876 | 7.716605 |
| \| | - | - | - | - | - | - |
| ELIX SUM MEDIAN ≥4 | 1.170898 | 0.0269299 | 6.86 | 0 | 1.119274 | 1.224903 |
| Congestive Heart Failure | 1.43313 | 0.0390797 | 13.2 | 0 | 1.358526 | 1.511831 |
| Cardiac Arrhythmias | 1.333052 | 0.0217357 | 17.63 | 0 | 1.291113 | 1.376353 |
| Valvular Disease | 0.9057439 | 0.0269924 | -3.32 | 0.001 | 0.8543414 | 0.9602392 |
| Pulmonary Circulation Disorders | 1.177222 | 0.0317379 | 6.05 | 0 | 1.116616 | 1.241118 |
| Peripheral Vascular Disorders | 1.053818 | 0.0298017 | 1.85 | 0.064 | 0.9969812 | 1.113894 |
| Hypertension Uncomplicated | 0.8128591 | 0.017355 | -9.7 | 0 | 0.7795367 | 0.8476059 |
| Paralysis | 1.131308 | 0.0643264 | 2.17 | 0.03 | 1.011971 | 1.264718 |
| Other Neurological Disorders | 1.405489 | 0.02631 | 18.18 | 0 | 1.354843 | 1.458029 |
| Chronic Pulmonary Disease | 1.054019 | 0.0177087 | 3.13 | 0.002 | 1.019867 | 1.089315 |
| Diabetes Uncomplicated | 1.064692 | 0.0236646 | 2.82 | 0.005 | 1.019294 | 1.112112 |
| Hypothyroidism | 0.9717421 | 0.0196026 | -1.42 | 0.155 | 0.9340611 | 1.010943 |
| Renal Failure | 0.9349649 | 0.023783 | -2.64 | 0.008 | 0.8894817 | 0.982774 |
| Liver Disease | 1.416837 | 0.0412594 | 11.96 | 0 | 1.338213 | 1.50008 |
| Peptic Ulcer Disease without Bleeding | 0.7309482 | 0.091475 | -2.5 | 0.012 | 0.5719169 | 0.934201 |
| AIDS HIV | 1.246323 | 0.1906347 | 1.44 | 0.15 | 0.9234144 | 1.682149 |
| Metastatic Cancer | 1.765763 | 0.1120151 | 8.96 | 0 | 1.559263 | 1.999609 |
| Solid Tumor Without Metastasis | 1.289886 | 0.0542814 | 6.05 | 0 | 1.187738 | 1.400818 |
| Lymphoma | 1.41385 | 0.0878369 | 5.57 | 0 | 1.251719 | 1.596981 |
| Rheumatoid Arthritis/Collagen Vascular | 1.130581 | 0.0422089 | 3.29 | 0.001 | 1.050786 | 1.216435 |
| Coagulopathy | 1.249504 | 0.0267682 | 10.4 | 0 | 1.198112 | 1.303101 |
| Weight Loss | 1.106436 | 0.0292073 | 3.83 | 0 | 1.050631 | 1.165205 |
| Fluid and Electrolyte Disorders | 1.189595 | 0.0222186 | 9.3 | 0 | 1.146823 | 1.233962 |
| Blood Loss Anemia | 0.9577071 | 0.091909 | -0.45 | 0.653 | 0.7934548 | 1.155961 |
| Deficiency Anemia | 0.8367193 | 0.0299168 | -4.99 | 0 | 0.7800757 | 0.897476 |
| Alcohol Abuse | 0.8901022 | 0.0437354 | -2.37 | 0.018 | 0.808359 | 0.9801115 |
| Psychoses | 1.104912 | 0.0546031 | 2.02 | 0.044 | 1.002885 | 1.217319 |
| Depression | 0.9642006 | 0.0219042 | -1.6 | 0.109 | 0.9221995 | 1.008115 |
| Hypertension Complicated | 0.8499129 | 0.0239613 | -5.77 | 0 | 0.8042113 | 0.8982117 |
| Aspirin | 0.7599527 | 0.0166681 | -12.52 | 0 | 0.7279675 | 0.7933433 |
| Long Term Anti Coagulation | 0.7168123 | 0.0166441 | -14.34 | 0 | 0.6849129 | 0.7501972 |
| Long Term NSAID | 0.6425317 | 0.0577789 | -4.92 | 0 | 0.5386791 | 0.7664062 |
| Long Term Steroid | 1.135654 | 0.0617392 | 2.34 | 0.019 | 1.020841 | 1.26338 |
| Tobacco Use Disorder | 0.7765513 | 0.0304296 | -6.45 | 0 | 0.7191279 | 0.8385601 |
| Coronary Artery Disease | 1.069631 | 0.0190291 | 3.78 | 0 | 1.032967 | 1.107596 |
| Hypomagnesemia | 0.6011206 | 0.024492 | -12.49 | 0 | 0.5549716 | 0.6511073 |
| Hyperhomocysteinemia | 0.5347996 | 0.4300463 | -0.78 | 0.436 | 0.1105423 | 2.587342 |
| Vitamin B12 Deficiency | 1.100214 | 0.2126422 | 0.49 | 0.621 | 0.7532115 | 1.60708 |
| Vitamin D Deficiency | 0.8765781 | 0.0428418 | -2.7 | 0.007 | 0.7964849 | 0.9647254 |
| History of Bariatric Surgery | 0.8526933 | 0.0914479 | -1.49 | 0.137 | 0.6910026 | 1.052219 |
| AKI | 2.088161 | 0.0387128 | 39.72 | 0 | 2.013627 | 2.165453 |
| Remdesivir | 1.12131 | 0.0258213 | 4.97 | 0 | 1.071813 | 1.173093 |
| Baricitinib | 1.42249 | 0.2821125 | 1.78 | 0.076 | 0.9642499 | 2.098499 |
| Vasopressor Use | 1.432724 | 0.0723123 | 7.12 | 0 | 1.297744 | 1.581744 |
| Cardiogenic Shock | 1.662754 | 0.1379121 | 6.13 | 0 | 1.413215 | 1.956356 |
| Septic Shock | 2.651828 | 0.0776722 | 33.3 | 0 | 2.503841 | 2.808562 |
| Mechanical Ventilation | 12.86445 | 0.4112712 | 79.9 | 0 | 12.0829 | 13.69655 |
| _cons | 0.0011921 | 0.0003078 | -26.08 | 0 | 0.0007187 | 0.0019776 |
| Note: P>\|t\|=0: p≤0.001  #: Interaction Term | | | | | | |

**Supplementary TABLE 7: Logistic Regression for Outcome Death with Mechanical Ventilation** **Stratified among Covid-19 Hospitalizations**

| Dependent Variable: Death | Odds ratio | Linearized Std. Err. | t | P>\|t\| | [95% conf. interval] | |
| --- | --- | --- | --- | --- | --- | --- |
| Independent Variable | - | - | - | - | Lower bound | Upper Bound |
| ACU | 0.4512439 | 0.0700975 | -5.12 | 0 | 0.332772 | 0.6118937 |
| Obesity | 0.8641877 | 0.0211162 | -5.97 | 0 | 0.8237651 | 0.9065937 |
| Diabetes Complicated | 0.988383 | 0.0192731 | -0.6 | 0.549 | 0.9513112 | 1.0269 |
| \| | - | - | - | - | - | - |
| Obesity # Diabetes Complicated | 1.207136 | 0.0426732 | 5.33 | 0 | 1.126308 | 1.293764 |
| \| | - | - | - | - | - | - |
| AGE GROUP (COMPARISON GROUP: 18-29 YEARS OLD) | - | - | - | - | - | - |
| AGE 30-54 | 2.13641 | 0.1681939 | 9.64 | 0 | 1.830852 | 2.492964 |
| AGE 55-64 | 4.378335 | 0.3473452 | 18.61 | 0 | 3.747677 | 5.115119 |
| AGE 65-79 | 10.3307 | 0.8530167 | 28.28 | 0 | 8.786701 | 12.14602 |
| AGE 80-85 | 20.27961 | 1.727018 | 35.34 | 0 | 17.16133 | 23.9645 |
| AGE 85-120 | 32.88706 | 2.817607 | 40.77 | 0 | 27.80212 | 38.90201 |
| \| | - | - | - | - | - | - |
| Racial or Ethnic Group (COMPARISON GROUP: White race) | - | - | - | - | - | - |
| Black | 0.7536371 | 0.0195377 | -10.91 | 0 | 0.7162904 | 0.7929309 |
| Hispanic | 0.9891697 | 0.0297222 | -0.36 | 0.717 | 0.9325822 | 1.049191 |
| Asian/Pacific Islander | 0.8802732 | 0.0409969 | -2.74 | 0.006 | 0.8034588 | 0.9644316 |
| Native American | 1.494239 | 0.1341827 | 4.47 | 0 | 1.253028 | 1.781882 |
| Other Racial or Ethnic Group | 0.9591747 | 0.0450547 | -0.89 | 0.375 | 0.8747897 | 1.0517 |
| \| | - | - | - | - | - | - |
| FEMALE (COMPARISON GROUP: MALE) | 0.8975892 | 0.0133782 | -7.25 | 0 | 0.8717407 | 0.9242041 |
| \| | - | - | - | - | - | - |
| PRIMARY PAYOR (COMPARISON GROUP: MEDICARE) | - | - | - | - | - | - |
| Medicaid | 1.203271 | 0.0404377 | 5.51 | 0 | 1.126548 | 1.28522 |
| Private Insurance | 1.094237 | 0.0380967 | 2.59 | 0.01 | 1.022041 | 1.171534 |
| Self-pay | 1.516528 | 0.1063277 | 5.94 | 0 | 1.321764 | 1.73999 |
| No charge | 1.146383 | 0.1892326 | 0.83 | 0.408 | 0.8294374 | 1.58444 |
| Other | 2.286866 | 0.1395549 | 13.55 | 0 | 2.029 | 2.577503 |
| \| | - | - | - | - | - | - |
| HOSP-BEDSIZE (COMPARISON GROUP: SMALL) | - | - | - | - | - | - |
| Medium | 1.106949 | 0.0382422 | 2.94 | 0.003 | 1.034457 | 1.18452 |
| Large | 1.03303 | 0.0349884 | 0.96 | 0.337 | 0.9666629 | 1.103954 |
| \| | - | - | - | - | - | - |
| HOSP-LOCTEACH (COMPARISON GROUP: RURAL) | - | - | - | - | - | - |
| Urban Nonteaching | 0.9633499 | 0.0423944 | -0.85 | 0.396 | 0.8837198 | 1.050155 |
| Urban Teaching | 0.9776862 | 0.0391664 | -0.56 | 0.573 | 0.9038381 | 1.057568 |
| \| | - | - | - | - | - | - |
| HOSP-REGION (COMPARISON GROUP: NORTHEAST) | - | - | - | - | - | - |
| Midwest | 0.7841514 | 0.0349663 | -5.45 | 0 | 0.7185108 | 0.8557887 |
| South | 0.8887154 | 0.0365095 | -2.87 | 0.004 | 0.8199449 | 0.9632538 |
| West | 0.8720868 | 0.0401828 | -2.97 | 0.003 | 0.7967616 | 0.9545332 |
| \| | - | - | - | - | - | - |
| ZIPINC-QRTL (COMPARISON GROUP: ZIPINC-QRTL 1) | - | - | - | - | - | - |
| 2 | 0.9061102 | 0.0214113 | -4.17 | 0 | 0.8650908 | 0.9490747 |
| 3 | 0.8308799 | 0.0219304 | -7.02 | 0 | 0.7889786 | 0.8750064 |
| 4 | 0.7842525 | 0.0253552 | -7.52 | 0 | 0.7360861 | 0.8355707 |
| \| | - | - | - | - | - | - |
| ADMISSION ON THE WEEKEND | 1.010579 | 0.0163763 | 0.65 | 0.516 | 0.9789778 | 1.0432 |
| \| | - | - | - | - | - | - |
| LOS (COMPARISON GROUP: 0-6 DAYS) | - | - | - | - | - | - |
| 7-13 days | 0.7940376 | 0.0186288 | -9.83 | 0 | 0.7583428 | 0.8314125 |
| 14-20 days | 0.5345982 | 0.0208002 | -16.1 | 0 | 0.4953357 | 0.5769728 |
| 21-29 days | 0.2470186 | 0.0137169 | -25.18 | 0 | 0.2215386 | 0.2754291 |
| 30-45 days | 0.0781147 | 0.0062459 | -31.89 | 0 | 0.066781 | 0.0913718 |
| 45-60 days | 0.0304998 | 0.0043199 | -24.64 | 0 | 0.0231047 | 0.0402618 |
| 61-780 days | 0.0191482 | 0.0044362 | -17.07 | 0 | 0.0121582 | 0.0301568 |
| \| | - | - | - | - | - | - |
| TOTCHG (COMPARISON GROUP: $0-$50,000) | - | - | - | - | - | - |
| 50000-99,999 | 1.164654 | 0.0294296 | 6.03 | 0 | 1.108363 | 1.223804 |
| 100k-249,999 | 1.885373 | 0.0764948 | 15.63 | 0 | 1.741214 | 2.041467 |
| 250k-499,999 | 2.848933 | 0.1933839 | 15.42 | 0 | 2.493947 | 3.254448 |
| 500k-1m | 4.118028 | 0.403006 | 14.46 | 0 | 3.399104 | 4.989008 |
| \| | - | - | - | - | - | - |
| ADMISSION MONTH (COMPARISON GROUP: JANUARY) | - | - | - | - | - | - |
| 2 | 1.319632 | 0.4513245 | 0.81 | 0.417 | 0.6749235 | 2.580186 |
| 3 | 5.59567 | 1.320677 | 7.3 | 0 | 3.522881 | 8.888046 |
| 4 | 6.102319 | 1.430042 | 7.72 | 0 | 3.854478 | 9.661046 |
| 5 | 3.806104 | 0.8924387 | 5.7 | 0 | 2.403474 | 6.027288 |
| 6 | 3.42609 | 0.8028454 | 5.26 | 0 | 2.164109 | 5.423984 |
| 7 | 3.731704 | 0.8721758 | 5.63 | 0 | 2.359983 | 5.900727 |
| 8 | 3.255568 | 0.7612788 | 5.05 | 0 | 2.05839 | 5.149037 |
| 9 | 3.311096 | 0.7785081 | 5.09 | 0 | 2.088243 | 5.250039 |
| 10 | 3.479929 | 0.81773 | 5.31 | 0 | 2.195309 | 5.516264 |
| 11 | 3.588189 | 0.836282 | 5.48 | 0 | 2.272139 | 5.66651 |
| 12 | 3.767874 | 0.8777121 | 5.69 | 0 | 2.386478 | 5.948884 |
| \| | - | - | - | - | - | - |
| ELIX SUM MEDIAN ≥4 | 1.190051 | 0.0282949 | 7.32 | 0 | 1.135851 | 1.246836 |
| Congestive Heart Failure | 1.479557 | 0.0416066 | 13.93 | 0 | 1.400195 | 1.563418 |
| Cardiac Arrhythmias | 1.316793 | 0.0220513 | 16.43 | 0 | 1.274263 | 1.360742 |
| Valvular Disease | 0.90104 | 0.0275903 | -3.4 | 0.001 | 0.8485406 | 0.9567876 |
| Pulmonary Circulation Disorders | 1.15067 | 0.0327762 | 4.93 | 0 | 1.088173 | 1.216756 |
| Peripheral Vascular Disorders | 1.046931 | 0.0305131 | 1.57 | 0.116 | 0.9887866 | 1.108494 |
| Hypertension Uncomplicated | 0.8003542 | 0.0176286 | -10.11 | 0 | 0.7665288 | 0.8356723 |
| Paralysis | 1.211333 | 0.0712889 | 3.26 | 0.001 | 1.079332 | 1.359478 |
| Other Neurological Disorders | 1.459521 | 0.0280173 | 19.7 | 0 | 1.405613 | 1.515495 |
| Chronic Pulmonary Disease | 1.057881 | 0.0183619 | 3.24 | 0.001 | 1.022488 | 1.094499 |
| Diabetes Uncomplicated | 1.061022 | 0.0238194 | 2.64 | 0.008 | 1.015337 | 1.108763 |
| Hypothyroidism | 0.9664575 | 0.0200134 | -1.65 | 0.1 | 0.9280069 | 1.006501 |
| Renal Failure | 0.9200894 | 0.0238139 | -3.22 | 0.001 | 0.8745668 | 0.9679815 |
| Liver Disease | 1.395932 | 0.0422695 | 11.02 | 0 | 1.315474 | 1.48131 |
| Peptic Ulcer Disease without Bleeding | 0.7259573 | 0.0920961 | -2.52 | 0.012 | 0.5661042 | 0.9309487 |
| AIDS HIV | 1.207533 | 0.1919757 | 1.19 | 0.236 | 0.8841708 | 1.649157 |
| Metastatic Cancer | 1.764043 | 0.1153016 | 8.68 | 0 | 1.551877 | 2.005215 |
| Solid Tumor Without Metastasis | 1.265897 | 0.0552594 | 5.4 | 0 | 1.162067 | 1.379004 |
| Lymphoma | 1.348223 | 0.0883874 | 4.56 | 0 | 1.185613 | 1.533136 |
| Rheumatoid Arthritis/Collagen Vascular | 1.102859 | 0.0430628 | 2.51 | 0.012 | 1.021585 | 1.190599 |
| Coagulopathy | 1.24121 | 0.0272827 | 9.83 | 0 | 1.188858 | 1.295867 |
| Weight Loss | 1.095816 | 0.029691 | 3.38 | 0.001 | 1.039126 | 1.155599 |
| Fluid and Electrolyte Disorders | 1.191783 | 0.0226743 | 9.22 | 0 | 1.148149 | 1.237076 |
| Blood Loss Anemia | 1.000782 | 0.0957943 | 0.01 | 0.993 | 0.8295454 | 1.207365 |
| Deficiency Anemia | 0.8390039 | 0.0307797 | -4.78 | 0 | 0.7807791 | 0.9015707 |
| Alcohol Abuse | 0.9154661 | 0.0472102 | -1.71 | 0.087 | 0.8274353 | 1.012863 |
| Psychoses | 1.125784 | 0.056919 | 2.34 | 0.019 | 1.019546 | 1.243092 |
| Depression | 0.9620494 | 0.0223987 | -1.66 | 0.097 | 0.9191237 | 1.00698 |
| Hypertension Complicated | 0.8320509 | 0.0239877 | -6.38 | 0 | 0.7863272 | 0.8804333 |
| Aspirin | 0.7569072 | 0.0168202 | -12.53 | 0 | 0.7246391 | 0.7906123 |
| Long Term Anti Coagulation | 0.7023704 | 0.016967 | -14.63 | 0 | 0.6698818 | 0.7364347 |
| Long Term NSAID | 0.6168679 | 0.0575391 | -5.18 | 0 | 0.5137754 | 0.7406465 |
| Long Term Steroid | 1.140115 | 0.0634673 | 2.36 | 0.019 | 1.022236 | 1.271587 |
| Tobacco Use Disorder | 0.7817862 | 0.0320763 | -6 | 0 | 0.7213632 | 0.8472703 |
| Coronary Artery Disease | 1.055407 | 0.0195085 | 2.92 | 0.004 | 1.017845 | 1.094354 |
| Hypomagnesemia | 0.5953615 | 0.0249998 | -12.35 | 0 | 0.5483124 | 0.6464477 |
| Hyperhomocysteinemia | 0.6577217 | 0.4661116 | -0.59 | 0.554 | 0.1639264 | 2.638975 |
| Vitamin B12 Deficiency | 1.134415 | 0.2237278 | 0.64 | 0.523 | 0.770642 | 1.669903 |
| Vitamin D Deficiency | 0.8662674 | 0.0437338 | -2.84 | 0.004 | 0.7846335 | 0.9563946 |
| History of Bariatric Surgery | 0.8389605 | 0.0964752 | -1.53 | 0.127 | 0.6696248 | 1.051118 |
| AKI | 2.118973 | 0.040251 | 39.53 | 0 | 2.041511 | 2.199373 |
| Remdesivir | 1.090444 | 0.0259032 | 3.64 | 0 | 1.040825 | 1.142429 |
| Baricitinib | 1.453611 | 0.2909528 | 1.87 | 0.062 | 0.9818061 | 2.152141 |
| Vasopressor Use | 1.435633 | 0.0745998 | 6.96 | 0 | 1.296582 | 1.589595 |
| Cardiogenic Shock | 1.704276 | 0.1549252 | 5.86 | 0 | 1.426071 | 2.036756 |
| Septic Shock | 2.91289 | 0.087877 | 35.44 | 0 | 2.745602 | 3.09037 |
| \| | - | - | - | - | - | - |
| Mechanical Ventilation  (Comparison group: ≤24 hours status post admission) | - | - | - | - | - | - |
| 24-48hr | 1.149194 | 0.0596324 | 2.68 | 0.007 | 1.038034 | 1.272257 |
| 2-5 days | 1.75001 | 0.0713288 | 13.73 | 0 | 1.615611 | 1.895589 |
| 6-13 days | 3.713921 | 0.1822394 | 26.74 | 0 | 3.373287 | 4.088952 |
| 14-730 days | 5.983134 | 0.4359708 | 24.55 | 0 | 5.186655 | 6.901922 |
| No Mechanical Ventilation Procedure | 0.1564818 | 0.0063384 | -45.79 | 0 | 0.1445358 | 0.1694151 |
| \| | - | - | - | - | - | - |
| _cons | 0.009085 | 0.0022916 | -18.64 | 0 | 0.0055407 | 0.0148967 |
| Note: P>\|t\|=0: p≤0.001  #: Interaction Term | | | | | | |

**Supplementary TABLE 8: Logistic Regression for Outcome Mechanical Ventilation** **among Covid-19 Hospitalizations**

| Dependent Variable: Mechanical Ventilation | Odds ratio | Linearized Std. Err. | t | P>\|t\| | [95% conf. interval] | |
| --- | --- | --- | --- | --- | --- | --- |
| Independent Variable | - | - | - | - | Lower bound | Upper Bound |
| ACU | 0.7997291 | 0.0847411 | -2.11 | 0.035 | 0.6497147 | 0.9843808 |
| Obesity | 1.232107 | 0.0289474 | 8.88 | 0 | 1.176642 | 1.290186 |
| Diabetes Complicated | 1.021005 | 0.0224642 | 0.94 | 0.345 | 0.9778997 | 1.066009 |
| \| | - | - | - | - | - | - |
| Obesity # Diabetes Complicated | 0.9934859 | 0.0326068 | -0.2 | 0.842 | 0.9315732 | 1.059513 |
| \| | - | - | - | - | - | - |
| AGE GROUP (COMPARISON GROUP: 18-29 YEARS OLD) | - | - | - | - | - | - |
| AGE 30-54 | 1.013364 | 0.0480928 | 0.28 | 0.78 | 0.9233311 | 1.112176 |
| AGE 55-64 | 1.262993 | 0.0610906 | 4.83 | 0 | 1.148728 | 1.388624 |
| AGE 65-79 | 1.289893 | 0.0646886 | 5.08 | 0 | 1.169106 | 1.423159 |
| AGE 80-85 | 0.9432259 | 0.0529484 | -1.04 | 0.298 | 0.8449284 | 1.052959 |
| AGE 85-120 | 0.5294286 | 0.031983 | -10.53 | 0 | 0.4702965 | 0.5959957 |
| \| | - | - | - | - | - | - |
| Racial or Ethnic Group (COMPARISON GROUP: White race) | - | - | - | - | - | - |
| Black | 0.8660069 | 0.0280909 | -4.44 | 0 | 0.812649 | 0.9228681 |
| Hispanic | 0.9482057 | 0.0377601 | -1.34 | 0.182 | 0.8769929 | 1.025201 |
| Asian/Pacific Islander | 1.043115 | 0.0500542 | 0.88 | 0.379 | 0.9494581 | 1.146011 |
| Native American | 2.095932 | 0.1882266 | 8.24 | 0 | 1.757573 | 2.499431 |
| Other Racial or Ethnic Group | 0.8786834 | 0.04957 | -2.29 | 0.022 | 0.7866826 | 0.9814435 |
| \| | - | - | - | - | - | - |
| FEMALE (COMPARISON GROUP: MALE) | 0.8842285 | 0.0136625 | -7.96 | 0 | 0.8578448 | 0.9114237 |
| \| | - | - | - | - | - | - |
| PRIMARY PAYOR (COMPARISON GROUP: MEDICARE) | - | - | - | - | - | - |
| Medicaid | 1.214639 | 0.0353519 | 6.68 | 0 | 1.147272 | 1.285963 |
| Private Insurance | 1.047094 | 0.0265187 | 1.82 | 0.069 | 0.9963733 | 1.100396 |
| Self-pay | 1.217758 | 0.0629311 | 3.81 | 0 | 1.100425 | 1.347601 |
| No charge | 0.9465937 | 0.1424789 | -0.36 | 0.715 | 0.7047051 | 1.27151 |
| Other | 1.198641 | 0.05191 | 4.18 | 0 | 1.101071 | 1.304856 |
| \| | - | - | - | - | - | - |
| HOSP-BEDSIZE (COMPARISON GROUP: SMALL) | - | - | - | - | - | - |
| Medium | 0.9525139 | 0.0482258 | -0.96 | 0.337 | 0.8625078 | 1.051912 |
| Large | 0.8443931 | 0.0418265 | -3.41 | 0.001 | 0.7662479 | 0.9305079 |
| \| | - | - | - | - | - | - |
| HOSP-LOCTEACH (COMPARISON GROUP: RURAL) | - | - | - | - | - | - |
| Urban Nonteaching | 0.5546906 | 0.032131 | -10.17 | 0 | 0.4951427 | 0.6213999 |
| Urban Teaching | 0.5699568 | 0.0287362 | -11.15 | 0 | 0.5163141 | 0.6291728 |
| \| | - | - | - | - | - | - |
| HOSP-REGION (COMPARISON GROUP: NORTHEAST) | - | - | - | - | - | - |
| Midwest | 1.665983 | 0.1150218 | 7.39 | 0 | 1.455077 | 1.907458 |
| South | 1.258265 | 0.088117 | 3.28 | 0.001 | 1.096846 | 1.44344 |
| West | 0.8947396 | 0.0689093 | -1.44 | 0.149 | 0.7693472 | 1.040569 |
| \| | - | - | - | - | - | - |
| ZIPINC-QRTL (COMPARISON GROUP: ZIPINC-QRTL 1) | - | - | - | - | - | - |
| 2 | 0.9793348 | 0.0300431 | -0.68 | 0.496 | 0.9221713 | 1.040042 |
| 3 | 0.9048406 | 0.0345796 | -2.62 | 0.009 | 0.8395244 | 0.9752384 |
| 4 | 0.7385518 | 0.0359733 | -6.22 | 0 | 0.6712885 | 0.8125549 |
| \| | - | - | - | - | - | - |
| ADMISSION ON THE WEEKEND | 1.053197 | 0.0175587 | 3.11 | 0.002 | 1.01933 | 1.08819 |
| \| | - | - | - | - | - | - |
| LOS (COMPARISON GROUP: 0-6 DAYS) | - | - | - | - | - | - |
| 7-13 days | 0.7534027 | 0.0240776 | -8.86 | 0 | 0.7076467 | 0.8021172 |
| 14-20 days | 0.8881316 | 0.0419101 | -2.51 | 0.012 | 0.8096529 | 0.9742173 |
| 21-29 days | 0.8122175 | 0.050601 | -3.34 | 0.001 | 0.7188329 | 0.9177338 |
| 30-45 days | 0.6291365 | 0.0513828 | -5.67 | 0 | 0.5360513 | 0.738386 |
| 45-60 days | 0.4820869 | 0.054554 | -6.45 | 0 | 0.3861667 | 0.6018328 |
| 61-780 days | 0.314549 | 0.0467401 | -7.78 | 0 | 0.2350552 | 0.4209269 |
| \| | - | - | - | - | - | - |
| TOTCHG (COMPARISON GROUP: $0-$50,000) | - | - | - | - | - | - |
| 50000-99,999 | 3.411337 | 0.1332422 | 31.42 | 0 | 3.159866 | 3.682821 |
| 100k-249,999 | 14.36185 | 0.8512349 | 44.96 | 0 | 12.78631 | 16.13153 |
| 250k-499,999 | 56.25793 | 4.86106 | 46.64 | 0 | 47.49128 | 66.64285 |
| 500k-1m | 142.843 | 17.1487 | 41.33 | 0 | 112.8864 | 180.7492 |
| \| | - | - | - | - | - | - |
| ADMISSION MONTH (COMPARISON GROUP: JANUARY) | - | - | - | - | - | - |
| 2 | 0.88066 | 0.2519619 | -0.44 | 0.657 | 0.5025823 | 1.543154 |
| 3 | 4.436371 | 0.7805923 | 8.47 | 0 | 3.142064 | 6.263841 |
| 4 | 2.327268 | 0.4047857 | 4.86 | 0 | 1.654835 | 3.272942 |
| 5 | 1.466569 | 0.261736 | 2.15 | 0.032 | 1.033589 | 2.080928 |
| 6 | 1.171263 | 0.2091322 | 0.89 | 0.376 | 0.8253304 | 1.66219 |
| 7 | 0.95287 | 0.1685947 | -0.27 | 0.785 | 0.6735748 | 1.347974 |
| 8 | 0.8754733 | 0.1564182 | -0.74 | 0.457 | 0.6167642 | 1.242701 |
| 9 | 0.9636508 | 0.1730474 | -0.21 | 0.837 | 0.6776775 | 1.370302 |
| 10 | 0.9250542 | 0.1644001 | -0.44 | 0.661 | 0.6529054 | 1.310642 |
| 11 | 0.9871739 | 0.1744074 | -0.07 | 0.942 | 0.6981799 | 1.39579 |
| 12 | 0.9893666 | 0.1744603 | -0.06 | 0.952 | 0.7001946 | 1.397963 |
| \| | - | - | - | - | - | - |
| ELIX SUM MEDIAN ≥4 | 1.199427 | 0.0309762 | 7.04 | 0 | 1.14021 | 1.26172 |
| Congestive Heart Failure | 1.326519 | 0.0386514 | 9.7 | 0 | 1.252866 | 1.404501 |
| Cardiac Arrhythmias | 1.671424 | 0.0317467 | 27.04 | 0 | 1.610329 | 1.734837 |
| Valvular Disease | 0.9035315 | 0.034575 | -2.65 | 0.008 | 0.8382272 | 0.9739235 |
| Pulmonary Circulation Disorders | 1.125777 | 0.0344952 | 3.87 | 0 | 1.06014 | 1.195477 |
| Peripheral Vascular Disorders | 0.8360789 | 0.0300396 | -4.98 | 0 | 0.7792124 | 0.8970956 |
| Hypertension Uncomplicated | 0.8257477 | 0.0178052 | -8.88 | 0 | 0.7915681 | 0.8614032 |
| Paralysis | 1.157029 | 0.0657778 | 2.57 | 0.01 | 1.034998 | 1.293447 |
| Other Neurological Disorders | 1.741465 | 0.0377017 | 25.62 | 0 | 1.669097 | 1.81697 |
| Chronic Pulmonary Disease | 1.08821 | 0.020412 | 4.51 | 0 | 1.048919 | 1.128973 |
| Diabetes Uncomplicated | 1.041331 | 0.0242794 | 1.74 | 0.082 | 0.994803 | 1.090036 |
| Hypothyroidism | 0.9039926 | 0.0208695 | -4.37 | 0 | 0.8639899 | 0.9458475 |
| Renal Failure | 0.631211 | 0.0180923 | -16.05 | 0 | 0.5967191 | 0.6676967 |
| Liver Disease | 1.496934 | 0.0419898 | 14.38 | 0 | 1.416835 | 1.581561 |
| Peptic Ulcer Disease without Bleeding | 0.9176263 | 0.1123993 | -0.7 | 0.483 | 0.7217288 | 1.166696 |
| AIDS HIV | 0.8036383 | 0.1054164 | -1.67 | 0.096 | 0.6214043 | 1.039314 |
| Metastatic Cancer | 0.7464975 | 0.0600183 | -3.64 | 0 | 0.6376361 | 0.8739444 |
| Solid Tumor Without Metastasis | 0.825808 | 0.0445691 | -3.55 | 0 | 0.7428939 | 0.9179763 |
| Lymphoma | 0.8566107 | 0.0689672 | -1.92 | 0.055 | 0.7315312 | 1.003077 |
| Rheumatoid Arthritis/Collagen Vascular | 1.05052 | 0.044116 | 1.17 | 0.241 | 0.9674954 | 1.14067 |
| Coagulopathy | 1.359522 | 0.0313946 | 13.3 | 0 | 1.299345 | 1.422486 |
| Weight Loss | 0.9095007 | 0.0283132 | -3.05 | 0.002 | 0.8556524 | 0.9667377 |
| Fluid and Electrolyte Disorders | 1.867977 | 0.040343 | 28.93 | 0 | 1.790536 | 1.948768 |
| Blood Loss Anemia | 0.874324 | 0.0809 | -1.45 | 0.147 | 0.729273 | 1.048225 |
| Deficiency Anemia | 0.7670811 | 0.0303307 | -6.71 | 0 | 0.7098639 | 0.8289101 |
| Alcohol Abuse | 0.8101965 | 0.0401063 | -4.25 | 0 | 0.7352628 | 0.8927671 |
| Psychoses | 0.8249012 | 0.039303 | -4.04 | 0 | 0.7513368 | 0.9056685 |
| Depression | 0.7858398 | 0.020728 | -9.14 | 0 | 0.7462353 | 0.8275461 |
| Hypertension Complicated | 0.7387791 | 0.0221227 | -10.11 | 0 | 0.696656 | 0.7834491 |
| Aspirin | 0.7863469 | 0.0202963 | -9.31 | 0 | 0.7475458 | 0.8271619 |
| Long Term Anti Coagulation | 0.6665493 | 0.0195764 | -13.81 | 0 | 0.6292536 | 0.7060555 |
| Long Term NSAID | 0.7666835 | 0.0639184 | -3.19 | 0.001 | 0.6510762 | 0.9028183 |
| Long Term Steroid | 1.032295 | 0.0594441 | 0.55 | 0.581 | 0.9220923 | 1.155669 |
| Tobacco Use Disorder | 0.9163275 | 0.0347407 | -2.3 | 0.021 | 0.8506878 | 0.9870321 |
| Coronary Artery Disease | 0.9114757 | 0.020213 | -4.18 | 0 | 0.872697 | 0.9519775 |
| Hypomagnesemia | 0.7591159 | 0.0333928 | -6.27 | 0 | 0.6963926 | 0.8274887 |
| Hyperhomocysteinemia | 1.586636 | 0.9597906 | 0.76 | 0.445 | 0.484646 | 5.194337 |
| Vitamin B12 Deficiency | 0.7383631 | 0.1977095 | -1.13 | 0.257 | 0.4368003 | 1.248122 |
| Vitamin D Deficiency | 0.758071 | 0.0400114 | -5.25 | 0 | 0.6835504 | 0.8407158 |
| History of Bariatric Surgery | 0.8232764 | 0.0797132 | -2.01 | 0.045 | 0.6809353 | 0.9953722 |
| AKI | 3.178667 | 0.0676846 | 54.31 | 0 | 3.048702 | 3.314172 |
| Remdesivir | 1.055568 | 0.0273558 | 2.09 | 0.037 | 1.003277 | 1.110585 |
| Baricitinib | 1.132005 | 0.2565653 | 0.55 | 0.584 | 0.7258932 | 1.765321 |
| _cons | 0.0119162 | 0.0023685 | -22.29 | 0 | 0.0080705 | 0.0175944 |
| Note: P>\|t\|=0: p≤0.001  #: Interaction Term | | | | | | |

**Supplementary TABLE 9: Logistic Regression for Outcome Acute Pulmonary Embolism among Covid-19 Hospitalizations**

| Dependent Variable: Acute PE | Odds ratio | Linearized Std. Err. | t | P>\|t\| | [95% conf. interval] | |
| --- | --- | --- | --- | --- | --- | --- |
| Independent Variable | - | - | - | - | Lower bound | Upper Bound |
| ACU | 0.5616253 | 0.094552 | -3.43 | 0.001 | 0.4037417 | 0.7812495 |
| Obesity | 0.7484431 | 0.0265726 | -8.16 | 0 | 0.6981191 | 0.8023947 |
| Diabetes Complicated | 0.5508625 | 0.0226393 | -14.51 | 0 | 0.508219 | 0.5970841 |
| \| | - | - | - | - | - | - |
| Obesity # Diabetes Complicated | 0.8973884 | 0.0548699 | -1.77 | 0.077 | 0.796013 | 1.011674 |
| \| | - | - | - | - | - | - |
| AGE GROUP (COMPARISON GROUP: 18-29 YEARS OLD) | - | - | - | - | - | - |
| AGE 30-54 | 2.033894 | 0.1626543 | 8.88 | 0 | 1.73875 | 2.379136 |
| AGE 55-64 | 2.502666 | 0.2110562 | 10.88 | 0 | 2.121285 | 2.952614 |
| AGE 65-79 | 2.634344 | 0.2264017 | 11.27 | 0 | 2.225861 | 3.117791 |
| AGE 80-85 | 2.583255 | 0.2451233 | 10 | 0 | 2.144741 | 3.111427 |
| AGE 85-120 | 2.384551 | 0.2259096 | 9.17 | 0 | 1.980352 | 2.871249 |
| \| | - | - | - | - | - | - |
| Racial or Ethnic Group (COMPARISON GROUP: White race) | - | - | - | - | - | - |
| Black | 1.360676 | 0.0447036 | 9.37 | 0 | 1.275797 | 1.451201 |
| Hispanic | 0.7334803 | 0.029647 | -7.67 | 0 | 0.6776003 | 0.7939685 |
| Asian/Pacific Islander | 0.6348342 | 0.057917 | -4.98 | 0 | 0.5308628 | 0.7591687 |
| Native American | 0.8965747 | 0.1349849 | -0.73 | 0.468 | 0.6674171 | 1.204414 |
| Other Racial or Ethnic Group | 0.8016949 | 0.0542185 | -3.27 | 0.001 | 0.7021445 | 0.9153595 |
| \| | - | - | - | - | - | - |
| FEMALE (COMPARISON GROUP: MALE) | 0.7891369 | 0.0207773 | -8.99 | 0 | 0.7494364 | 0.8309405 |
| \| | - | - | - | - | - | - |
| PRIMARY PAYOR (COMPARISON GROUP: MEDICARE) | - | - | - | - | - | - |
| Medicaid | 1.009701 | 0.0462611 | 0.21 | 0.833 | 0.9229593 | 1.104594 |
| Private Insurance | 1.262503 | 0.0456372 | 6.45 | 0 | 1.176128 | 1.355222 |
| Self-pay | 1.201549 | 0.0842421 | 2.62 | 0.009 | 1.047239 | 1.378595 |
| No charge | 1.245476 | 0.284151 | 0.96 | 0.336 | 0.7963117 | 1.947995 |
| Other | 1.118124 | 0.0716379 | 1.74 | 0.081 | 0.9861398 | 1.267772 |
| \| | - | - | - | - | - | - |
| HOSP-BEDSIZE (COMPARISON GROUP: SMALL) | - | - | - | - | - | - |
| Medium | 1.148755 | 0.045486 | 3.5 | 0 | 1.062953 | 1.241483 |
| Large | 1.092464 | 0.0398287 | 2.43 | 0.015 | 1.017105 | 1.173406 |
| \| | - | - | - | - | - | - |
| HOSP-LOCTEACH (COMPARISON GROUP: RURAL) | - | - | - | - | - | - |
| Urban Nonteaching | 0.9671505 | 0.0551715 | -0.59 | 0.558 | 0.8648155 | 1.081595 |
| Urban Teaching | 0.9886099 | 0.0500006 | -0.23 | 0.821 | 0.8952866 | 1.091661 |
| \| | - | - | - | - | - | - |
| HOSP-REGION (COMPARISON GROUP: NORTHEAST) | - | - | - | - | - | - |
| Midwest | 1.3548 | 0.0658393 | 6.25 | 0 | 1.23168 | 1.490228 |
| South | 1.006346 | 0.0481919 | 0.13 | 0.895 | 0.9161647 | 1.105404 |
| West | 0.8956287 | 0.0510063 | -1.94 | 0.053 | 0.8010108 | 1.001423 |
| \| | - | - | - | - | - | - |
| ZIPINC-QRTL (COMPARISON GROUP: ZIPINC-QRTL 1) | - | - | - | - | - | - |
| 2 | 1.042685 | 0.0326819 | 1.33 | 0.182 | 0.9805408 | 1.108768 |
| 3 | 1.03301 | 0.0377821 | 0.89 | 0.375 | 0.9615314 | 1.109803 |
| 4 | 1.039972 | 0.0443817 | 0.92 | 0.358 | 0.9565019 | 1.130726 |
| \| | - | - | - | - | - | - |
| ADMISSION ON THE WEEKEND | 0.9418051 | 0.0261017 | -2.16 | 0.031 | 0.891998 | 0.9943934 |
| \| | - | - | - | - | - | - |
| LOS (COMPARISON GROUP: 0-6 DAYS) | - | - | - | - | - | - |
| 7-13 days | 1.073611 | 0.0376102 | 2.03 | 0.043 | 1.002351 | 1.149937 |
| 14-20 days | 1.232739 | 0.0632729 | 4.08 | 0 | 1.114729 | 1.363242 |
| 21-29 days | 1.350428 | 0.0930218 | 4.36 | 0 | 1.179837 | 1.545686 |
| 30-45 days | 1.394111 | 0.1222409 | 3.79 | 0 | 1.173925 | 1.655597 |
| 45-60 days | 1.397424 | 0.2035176 | 2.3 | 0.022 | 1.050333 | 1.859215 |
| 61-780 days | 1.402318 | 0.3301743 | 1.44 | 0.151 | 0.8838456 | 2.224931 |
| \| | - | - | - | - | - | - |
| TOTCHG (COMPARISON GROUP: $0-$50,000) | - | - | - | - | - | - |
| 50000-99,999 | 1.490104 | 0.0523087 | 11.36 | 0 | 1.391001 | 1.596266 |
| 100k-249,999 | 1.903404 | 0.0966114 | 12.68 | 0 | 1.723116 | 2.102556 |
| 250k-499,999 | 2.062657 | 0.1592119 | 9.38 | 0 | 1.772991 | 2.399648 |
| 500k-1m | 1.98468 | 0.2097509 | 6.49 | 0 | 1.613268 | 2.441601 |
| \| | - | - | - | - | - | - |
| ADMISSION MONTH (COMPARISON GROUP: JANUARY) | - | - | - | - | - | - |
| 2 | 1.798203 | 0.9063566 | 1.16 | 0.244 | 0.6693986 | 4.830505 |
| 3 | 0.8599775 | 0.3341401 | -0.39 | 0.698 | 0.4014812 | 1.842082 |
| 4 | 2.059076 | 0.7821662 | 1.9 | 0.057 | 0.9777845 | 4.336123 |
| 5 | 2.109216 | 0.8011326 | 1.96 | 0.049 | 1.001669 | 4.441381 |
| 6 | 1.617096 | 0.6147769 | 1.26 | 0.206 | 0.7674362 | 3.407451 |
| 7 | 2.111916 | 0.7988356 | 1.98 | 0.048 | 1.006049 | 4.433371 |
| 8 | 2.870909 | 1.089993 | 2.78 | 0.005 | 1.363815 | 6.043424 |
| 9 | 2.412113 | 0.9204591 | 2.31 | 0.021 | 1.141538 | 5.096887 |
| 10 | 2.534311 | 0.9588709 | 2.46 | 0.014 | 1.207018 | 5.321156 |
| 11 | 2.734279 | 1.030885 | 2.67 | 0.008 | 1.305664 | 5.726035 |
| 12 | 3.122224 | 1.176339 | 3.02 | 0.003 | 1.491674 | 6.53513 |
| \| | - | - | - | - | - | - |
| ELIX SUM MEDIAN ≥4 | 9.152114 | 0.4809847 | 42.13 | 0 | 8.256093 | 10.14538 |
| Congestive Heart Failure | 1.179969 | 0.0559469 | 3.49 | 0 | 1.075228 | 1.294913 |
| Cardiac Arrhythmias | 0.5236514 | 0.0188077 | -18.01 | 0 | 0.4880471 | 0.5618531 |
| Valvular Disease | 0.7271051 | 0.0472463 | -4.9 | 0 | 0.6401356 | 0.8258904 |
| Peripheral Vascular Disorders | 0.6491426 | 0.0384682 | -7.29 | 0 | 0.5779416 | 0.7291155 |
| Hypertension Uncomplicated | 0.5704285 | 0.0186447 | -17.17 | 0 | 0.5350221 | 0.6081781 |
| Paralysis | 0.4739963 | 0.0521049 | -6.79 | 0 | 0.3821019 | 0.5879909 |
| Other Neurological Disorders | 0.5659877 | 0.0209872 | -15.35 | 0 | 0.5263022 | 0.6086657 |
| Chronic Pulmonary Disease | 0.6025482 | 0.020357 | -14.99 | 0 | 0.5639311 | 0.6438097 |
| Diabetes Uncomplicated | 0.538293 | 0.0207263 | -16.09 | 0 | 0.4991547 | 0.5805001 |
| Hypothyroidism | 0.6217292 | 0.0247764 | -11.93 | 0 | 0.5750039 | 0.6722514 |
| Renal Failure | 0.4612486 | 0.022954 | -15.55 | 0 | 0.4183726 | 0.5085186 |
| Liver Disease | 0.7001381 | 0.0366296 | -6.81 | 0 | 0.6318856 | 0.7757628 |
| Peptic Ulcer Disease without Bleeding | 0.9496747 | 0.1678833 | -0.29 | 0.77 | 0.6715185 | 1.343049 |
| AIDS HIV | 0.701154 | 0.1696583 | -1.47 | 0.142 | 0.4363059 | 1.126771 |
| Metastatic Cancer | 0.8764476 | 0.0886813 | -1.3 | 0.193 | 0.7187457 | 1.068751 |
| Solid Tumor Without Metastasis | 0.8916676 | 0.0642887 | -1.59 | 0.112 | 0.7741317 | 1.027049 |
| Lymphoma | 0.5715018 | 0.076734 | -4.17 | 0 | 0.4392355 | 0.7435974 |
| Rheumatoid Arthritis/Collagen Vascular | 0.6801813 | 0.0489323 | -5.36 | 0 | 0.5907072 | 0.783208 |
| Coagulopathy | 1.001589 | 0.0362572 | 0.04 | 0.965 | 0.9329701 | 1.075255 |
| Weight Loss | 0.6626385 | 0.0322881 | -8.45 | 0 | 0.6022671 | 0.7290617 |
| Fluid and Electrolyte Disorders | 0.5153532 | 0.0153152 | -22.31 | 0 | 0.4861856 | 0.5462707 |
| Blood Loss Anemia | 1.144382 | 0.1619048 | 0.95 | 0.341 | 0.8671843 | 1.510187 |
| Deficiency Anemia | 0.7342925 | 0.0462789 | -4.9 | 0 | 0.6489438 | 0.8308661 |
| Alcohol Abuse | 0.4006631 | 0.0356766 | -10.27 | 0 | 0.3364838 | 0.4770838 |
| Psychoses | 0.4973974 | 0.0450285 | -7.71 | 0 | 0.4165091 | 0.5939948 |
| Depression | 0.5554603 | 0.0237798 | -13.73 | 0 | 0.5107427 | 0.6040931 |
| Hypertension Complicated | 0.5247302 | 0.0257232 | -13.15 | 0 | 0.4766473 | 0.5776636 |
| Aspirin | 0.8745419 | 0.0329934 | -3.55 | 0 | 0.8121922 | 0.9416779 |
| Long Term Anticoagulation | 1.734314 | 0.0709697 | 13.46 | 0 | 1.600612 | 1.879184 |
| Long Term NSAID | 1.165305 | 0.135603 | 1.31 | 0.189 | 0.9275998 | 1.463925 |
| Long Term Steroid | 0.9147566 | 0.0863254 | -0.94 | 0.345 | 0.7602486 | 1.100666 |
| Tobacco Use Disorder | 0.9239963 | 0.0552901 | -1.32 | 0.187 | 0.8217163 | 1.039007 |
| Coronary Artery Disease | 0.772326 | 0.0287789 | -6.93 | 0 | 0.7179164 | 0.8308593 |
| Hypomagnesemia | 0.814642 | 0.0563547 | -2.96 | 0.003 | 0.7113228 | 0.9329682 |
| Hyperhomocysteinemia | 4.966945 | 2.88453 | 2.76 | 0.006 | 1.590821 | 15.50806 |
| Vitamin B12 Deficiency | 0.7103554 | 0.3362996 | -0.72 | 0.47 | 0.2807918 | 1.797078 |
| Vitamin D Deficiency | 0.903384 | 0.0774523 | -1.19 | 0.236 | 0.7636139 | 1.068737 |
| History of Bariatric Surgery | 0.9087612 | 0.1277516 | -0.68 | 0.496 | 0.6898531 | 1.197134 |
| AKI | 0.9237075 | 0.0285522 | -2.57 | 0.01 | 0.8693931 | 0.9814151 |
| Remdesivir | 0.8325244 | 0.0271568 | -5.62 | 0 | 0.7809501 | 0.8875047 |
| Baricitinib | 0.7411733 | 0.2554794 | -0.87 | 0.385 | 0.3770795 | 1.456823 |
| Mechanical Ventilation | 1.39749 | 0.0579614 | 8.07 | 0 | 1.288354 | 1.515872 |
| _cons | 0.0050893 | 0.0020005 | -13.43 | 0 | 0.0023549 | 0.0109986 |
| Note: P>\|t\|=0: p≤0.001  #: Interaction Term | | | | | | |

**Supplementary TABLE 10: Logistic Regression for Outcome Covid-19 Disease among All-Cause Hospitalizations** **March to December 2020**

| **Dependent Variable:**  Covid-19 | Odds Ratio | Linearized Std. Err. | t | P>\|t\| | [95% Confidence Interval | |
| --- | --- | --- | --- | --- | --- | --- |
|  |  |  |  |  | Lower Bound | Upper Bound |
| ACU | 0.3886603 | 0.0095309 | -38.54 | 0 | 0.370417 | 0.4078022 |
| 1.Obesity | 1.60155 | 0.0148326 | 50.85 | 0 | 1.572733 | 1.630895 |
| 1.DiabetesComplicated | 1.355762 | 0.0095086 | 43.4 | 0 | 1.337248 | 1.374532 |
| \| |  |  |  |  |  |  |
| Obesity#DiabetesComplicated |  |  |  |  |  |  |
| 1 | 0.8581794 | 0.0096357 | -13.62 | 0 | 0.8394949 | 0.8772798 |
| \| |  |  |  |  |  |  |
| AGE GROUP (COMPARISON GROUP: 18-29 YEARS OLD) |  |  |  |  |  |  |
| AGE 30-54 | 2.188446 | 0.0276705 | 61.94 | 0 | 2.134865 | 2.243372 |
| AGE 55-64 | 3.084951 | 0.0436498 | 79.62 | 0 | 3.000552 | 3.171725 |
| AGE 65-79 | 3.646963 | 0.0562914 | 83.83 | 0 | 3.538256 | 3.759009 |
| AGE 80-85 | 4.152175 | 0.0692499 | 85.36 | 0 | 4.018606 | 4.290184 |
| AGE 85-120 | 4.527384 | 0.0764855 | 89.39 | 0 | 4.37989 | 4.679845 |
| \| |  |  |  |  |  |  |
| Racial or Ethnic Group (COMPARISON GROUP: White race) |  |  |  |  |  |  |
| Black | 1.851452 | 0.0224802 | 50.73 | 0 | 1.8079 | 1.896054 |
| Hispanic | 3.095114 | 0.0509542 | 68.63 | 0 | 2.996813 | 3.196639 |
| Asian/Pacific Islander | 1.772016 | 0.0389707 | 26.01 | 0 | 1.697237 | 1.850089 |
| Native American | 2.258914 | 0.123094 | 14.95 | 0 | 2.030031 | 2.513603 |
| Other Racial or Ethnic Group | 2.186605 | 0.0603811 | 28.33 | 0 | 2.071374 | 2.308245 |
| \| |  |  |  |  |  |  |
| FEMALE (COMPARISON GROUP: MALE) | 0.652837 | 0.0037454 | -74.33 | 0 | 0.6455352 | 0.6602213 |
| \| |  |  |  |  |  |  |
| PRIMARY PAYOR (COMPARISON GROUP: MEDICARE) |  |  |  |  |  |  |
| Medicaid | 1.031749 | 0.0124003 | 2.6 | 0.009 | 1.007722 | 1.056348 |
| Private Insurance | 1.280742 | 0.012518 | 25.32 | 0 | 1.256434 | 1.30552 |
| Self-pay | 1.016199 | 0.0281652 | 0.58 | 0.562 | 0.9624545 | 1.072945 |
| No charge | 0.828711 | 0.149095 | -1.04 | 0.296 | 0.5823973 | 1.179198 |
| Other | 1.606025 | 0.0393353 | 19.34 | 0 | 1.53073 | 1.685024 |
| \| |  |  |  |  |  |  |
| HOSP_BEDSIZE (COMPARISON GROUP: SMALL) |  |  |  |  |  |  |
| Medium | 0.9713768 | 0.0203806 | -1.38 | 0.166 | 0.9322312 | 1.012166 |
| Large | 0.901165 | 0.0185789 | -5.05 | 0 | 0.8654672 | 0.9383352 |
| \| |  |  |  |  |  |  |
| HOSP_LOCTEACH (COMPARISON GROUP: RURAL) |  |  |  |  |  |  |
| Urban Nonteaching | 0.8495456 | 0.0213097 | -6.5 | 0 | 0.8087784 | 0.8923676 |
| Urban Teaching | 0.7776687 | 0.0172337 | -11.35 | 0 | 0.7446053 | 0.8122003 |
| \| |  |  |  |  |  |  |
| HOSP_REGION (COMPARISON GROUP: NORTHEAST) |  |  |  |  |  |  |
| Northeast | 1.348839 | 0.0375389 | 10.75 | 0 | 1.277215 | 1.424479 |
| Midwest | 1.452612 | 0.0366216 | 14.81 | 0 | 1.382561 | 1.526213 |
| South | 1.175873 | 0.0275487 | 6.92 | 0 | 1.123085 | 1.231142 |
| \| |  |  |  |  |  |  |
| ZIPINC_QRTL (COMPARISON GROUP: ZIPINC-QRTL 1) |  |  |  |  |  |  |
| 2 | 0.941383 | 0.010308 | -5.52 | 0 | 0.9213895 | 0.9618104 |
| 3 | 0.9217926 | 0.0122253 | -6.14 | 0 | 0.8981336 | 0.9460748 |
| 4 | 0.8338805 | 0.0145069 | -10.44 | 0 | 0.8059192 | 0.8628119 |
| \| |  |  |  |  |  |  |
| ADMISSION ON THE WEEKEND | 1.260439 | 0.0062061 | 47.01 | 0 | 1.24833 | 1.272665 |
| \| |  |  |  |  |  |  |
| ADMISSION MONTH (COMPARISON GROUP: MARCH) |  |  |  |  |  |  |
| 4 | 3.231847 | 0.0680118 | 55.74 | 0 | 3.101222 | 3.367973 |
| 5 | 1.458674 | 0.0565488 | 9.74 | 0 | 1.351918 | 1.57386 |
| 6 | 1.270559 | 0.0551236 | 5.52 | 0 | 1.166957 | 1.383358 |
| 7 | 2.321747 | 0.1009254 | 19.38 | 0 | 2.132079 | 2.528288 |
| 8 | 1.554777 | 0.0650937 | 10.54 | 0 | 1.432258 | 1.687778 |
| 9 | 1.142086 | 0.0481501 | 3.15 | 0.002 | 1.051484 | 1.240496 |
| 10 | 1.809799 | 0.0764596 | 14.04 | 0 | 1.665939 | 1.966081 |
| 11 | 4.169869 | 0.173549 | 34.31 | 0 | 3.843137 | 4.52438 |
| 12 | 4.496096 | 0.1888398 | 35.79 | 0 | 4.140707 | 4.881987 |
| \| |  |  |  |  |  |  |
| ELIXSUM MEDIAN≥4 | 0.8580397 | 0.0067469 | -19.47 | 0 | 0.8449138 | 0.8713696 |
| Congestive Heart Failure | 0.670243 | 0.0063838 | -42.01 | 0 | 0.6578436 | 0.6828761 |
| Cardiac Arrhythmias | 1.049852 | 0.0068309 | 7.48 | 0 | 1.036545 | 1.06333 |
| Valvular Disease | 0.5618195 | 0.0064849 | -49.95 | 0 | 0.5492486 | 0.5746781 |
| Pulmonary Circulation Disorders | 1.00322 | 0.0105071 | 0.31 | 0.759 | 0.9828309 | 1.024032 |
| Peripheral Vascular Disorders | 0.6638488 | 0.0074568 | -36.47 | 0 | 0.6493894 | 0.6786302 |
| Hypertension Uncomplicated | 1.07016 | 0.006946 | 10.45 | 0 | 1.056629 | 1.083865 |
| Paralysis | 0.4246482 | 0.0077058 | -47.2 | 0 | 0.4098064 | 0.4400275 |
| Other Neurological Disorders | 1.170059 | 0.0082421 | 22.3 | 0 | 1.154012 | 1.18633 |
| Chronic Pulmonary Disease | 1.180714 | 0.0071825 | 27.31 | 0 | 1.166716 | 1.19488 |
| Diabetes Uncomplicated | 1.24633 | 0.008933 | 30.72 | 0 | 1.228939 | 1.263967 |
| Hypothyroidism | 1.031158 | 0.0066343 | 4.77 | 0 | 1.018233 | 1.044247 |
| Renal Failure | 0.8472828 | 0.0073771 | -19.03 | 0 | 0.8329427 | 0.8618698 |
| Liver Disease | 0.7178645 | 0.0076608 | -31.06 | 0 | 0.7030014 | 0.7330417 |
| Peptic Ulcer Disease without Bleeding | 0.4332885 | 0.0174608 | -20.75 | 0 | 0.4003738 | 0.4689091 |
| AIDSHIV | 0.7220917 | 0.0345841 | -6.8 | 0 | 0.6573752 | 0.7931792 |
| Metastatic Cancer | 0.4351702 | 0.0088832 | -40.76 | 0 | 0.4180985 | 0.4529391 |
| Solid Tumor Without Metastasis | 0.3627959 | 0.0051372 | -71.6 | 0 | 0.3528629 | 0.3730084 |
| Lymphoma | 0.5004732 | 0.0133598 | -25.93 | 0 | 0.4749549 | 0.5273626 |
| Rheumatoid Arthritis/ Collagen Vascular | 0.9329407 | 0.012037 | -5.38 | 0 | 0.909638 | 0.9568403 |
| Coagulopathy | 1.834473 | 0.0264152 | 42.14 | 0 | 1.783409 | 1.886998 |
| Weight Loss | 0.9941518 | 0.012266 | -0.48 | 0.635 | 0.9703927 | 1.018493 |
| Fluid and Electrolyte Disorders | 2.1279 | 0.0145574 | 110.38 | 0 | 2.099551 | 2.156633 |
| Blood Loss Anemia | 0.5459222 | 0.0154963 | -21.32 | 0 | 0.5163716 | 0.577164 |
| Deficiency Anemia | 0.7477331 | 0.0087699 | -24.79 | 0 | 0.7307359 | 0.7651256 |
| Alcohol Abuse | 0.3532384 | 0.0052662 | -69.8 | 0 | 0.3430634 | 0.3637152 |
| Psychoses | 0.8622127 | 0.0172479 | -7.41 | 0 | 0.8290525 | 0.8966991 |
| Depression | 0.8800997 | 0.0067553 | -16.64 | 0 | 0.8669551 | 0.8934437 |
| Hypertension Complicated | 1.007202 | 0.0101882 | 0.71 | 0.478 | 0.9874243 | 1.027375 |
| Aspirin | 0.9054568 | 0.0076638 | -11.73 | 0 | 0.8905558 | 0.920607 |
| Long Term Anticoagulation | 0.9070741 | 0.0083445 | -10.6 | 0 | 0.8908613 | 0.923582 |
| Long Term NSAID | 0.8486597 | 0.0224984 | -6.19 | 0 | 0.805678 | 0.8939343 |
| Long Term Steroid | 1.154856 | 0.0218829 | 7.6 | 0 | 1.112741 | 1.198564 |
| Tobacco Use Disorder | 0.3591396 | 0.0040805 | -90.13 | 0 | 0.3512282 | 0.3672291 |
| Coronary Artery Disease | 0.7616575 | 0.0052405 | -39.57 | 0 | 0.7514525 | 0.7720011 |
| Hypomagnesemia | 0.703734 | 0.0106216 | -23.28 | 0 | 0.6832154 | 0.7248688 |
| Hyperhomocysteinemia | 0.6387634 | 0.1312075 | -2.18 | 0.029 | 0.4270193 | 0.955504 |
| Vitamin D Deficiency | 1.081211 | 0.0248809 | 3.39 | 0.001 | 1.033516 | 1.131108 |
| History of Bariatric Surgery | 0.5875282 | 0.0140667 | -22.21 | 0 | 0.5605876 | 0.6157635 |
| _cons | 0.0109147 | 0.0005067 | -97.31 | 0 | 0.0099651 | 0.0119547 |
| Note: P>\|t\|=0: p≤0.001  #: Interaction Term | | | | | | |

**Supplementary TABLE 11: Propensity Score Matched Sample Logistic Regression for Primary Outcome Death among Covid-19 Disease Hospitalizations** **January to December 2020**

| Log likelihood = -17370.266 | Number of obs = 283,462  LR chi2(73) = 152408.91  Prob > chi2 = 0.0000  Pseudo R2 = 0.8144 | | | | | |
| --- | --- | --- | --- | --- | --- | --- |
| **Dependent Variable:**  DEATH | Odds Ratio | Linearized Std. Err. | t | P>\|t\| | [95% Confidence Interval | |
|  |  |  |  |  | Lower Bound | Upper Bound |

| ACU | 0.1602586 | 0.0375478 | -7.81 | 0 | 0.1012486 | 0.253661 |
| --- | --- | --- | --- | --- | --- | --- |
| \| |  |  |  |  |  |  |
| Length of Stay in days (Comparison group 0-6 days) |  |  |  |  |  |  |
| 7-13 days | 0.0704698 | 0.0045349 | -41.22 | 0 | 0.0621193 | 0.0799429 |
| 14-20 days | 1.252499 | 0.0955513 | 2.95 | 0.003 | 1.07855 | 1.454502 |
| 21-29 days | 0.0590975 | 0.0059401 | -28.14 | 0 | 0.0485301 | 0.071966 |
| 30-45 days | 0.0013672 | 0.0002459 | -36.66 | 0 | 0.000961 | 0.0019451 |
| 45-60 days | 8.261513 | 1.178828 | 14.8 | 0 | 6.246007 | 10.9274 |
| 61-780 days | 0.0973757 | 0.1834251 | -1.24 | 0.216 | 0.0024269 | 3.907018 |
| \| |  |  |  |  |  |  |
| AGE in years, y (Comparison group 18-29y) |  |  |  |  |  |  |
| 30-54 years old | 2.711682 | 0.4010581 | 6.74 | 0 | 2.029298 | 3.623527 |
| 55-64 years old | 11.85777 | 1.810328 | 16.2 | 0 | 8.791236 | 15.99396 |
| 65-79 years old | 122.1825 | 19.94878 | 29.43 | 0 | 88.72235 | 168.2616 |
| 80-85 years old | 0.6291449 | 0.1121307 | -2.6 | 0.009 | 0.443653 | 0.8921911 |
| 85-120 years old | 0.0064795 | 0.004917 | -6.64 | 0 | 0.0014642 | 0.0286735 |
| \| |  |  |  |  |  |  |
| Racial or Ethnic Group (Comparison group White race) |  |  |  |  |  |  |
| Black | 0.6680249 | 0.0381451 | -7.07 | 0 | 0.5972936 | 0.747132 |
| Hispanic | 0.0145262 | 0.0017271 | -35.59 | 0 | 0.0115066 | 0.0183382 |
| Asian/Pacific Islander | 8.87673 | 1.940717 | 9.99 | 0 | 5.783025 | 13.62545 |
| Native American | 0.0466698 | 0.0080917 | -17.68 | 0 | 0.033224 | 0.065557 |
| Other Racial or Ethnic Group | 0.4004107 | 0.051788 | -7.08 | 0 | 0.3107517 | 0.5159383 |
| \| |  |  |  |  |  |  |
| FEMALE (Comparison group Male Sex) | 3.160269 | 0.2080011 | 17.48 | 0 | 2.777794 | 3.595407 |
| \| |  |  |  |  |  |  |
| Primary Payor (Comparison group Medicare) |  |  |  |  |  |  |
| Medicaid | 0.0420406 | 0.0043998 | -30.28 | 0 | 0.0342441 | 0.0516123 |
| Private | 0.0209053 | 0.0019489 | -41.49 | 0 | 0.0174142 | 0.0250962 |
| Self-pay | 28.65101 | 4.029241 | 23.86 | 0 | 21.74874 | 37.74382 |
| No Charge | 0.1702455 | 0.999217 | -0.3 | 0.763 | 1.72E-06 | 16865.81 |
| Other | 1.851246 | 0.2576205 | 4.43 | 0 | 1.409322 | 2.431744 |
| \| |  |  |  |  |  |  |
| HOSPITAL BEDSIZE (Comparison group small bed size) |  |  |  |  |  |  |
| Medium | 0.0473899 | 0.0038857 | -37.19 | 0 | 0.0403545 | 0.0556519 |
| Large | 0.7896827 | 0.0585981 | -3.18 | 0.001 | 0.6827939 | 0.9133047 |
| \| |  |  |  |  |  |  |
| HOSPITAL LOCATION/TEACH STATUS (Comparison group Rural) |  |  |  |  |  |  |
| Urban | 0.7273998 | 0.0600179 | -3.86 | 0 | 0.6187858 | 0.8550786 |
| Urban | 0.0110546 | 0.001015 | -49.06 | 0 | 0.009234 | 0.0132342 |
| \| |  |  |  |  |  |  |
| HOSPITAL REGION (Comparison group Northeast) |  |  |  |  |  |  |
| Midwest | 0.1348315 | 0.011654 | -23.18 | 0 | 0.11382 | 0.1597216 |
| South | 0.0259027 | 0.002473 | -38.27 | 0 | 0.0214821 | 0.0312329 |
| West | 0.0543209 | 0.0052824 | -29.95 | 0 | 0.0448944 | 0.0657267 |
| \| |  |  |  |  |  |  |
| ZIPINC_QRTL (Comparison group ZIPINC_QRTL1) |  |  |  |  |  |  |
| 2 | 0.0654722 | 0.005126 | -34.82 | 0 | 0.0561583 | 0.0763309 |
| 3 | 1.677696 | 0.1200757 | 7.23 | 0 | 1.458113 | 1.930346 |
| 4 | 0.008627 | 0.0012351 | -33.2 | 0 | 0.0065161 | 0.0114216 |
| \| |  |  |  |  |  |  |
| ADMISSION ON THE WEEKEND | 1.598457 | 0.0871597 | 8.6 | 0 | 1.436439 | 1.77875 |
| Admission Month: April | 8.690421 | 0.8387626 | 22.4 | 0 | 7.192609 | 10.50014 |
| Congestive Heart Failure | 10.1385 | 0.9248424 | 25.39 | 0 | 8.478646 | 12.12331 |
| Cardiac Arrhythmias | 15.74772 | 0.9914401 | 43.79 | 0 | 13.91964 | 17.81589 |
| Valvular Disease | 0.37986 | 0.0418724 | -8.78 | 0 | 0.3060516 | 0.4714681 |
| Pulmonary Circulation Disorders | 39.74407 | 3.521909 | 41.56 | 0 | 33.40745 | 47.2826 |
| Peripheral Vascular Disorders | 0.0103924 | 0.0012058 | -39.36 | 0 | 0.0082785 | 0.0130461 |
| Paralysis | 2.788184 | 0.3627847 | 7.88 | 0 | 2.160565 | 3.598119 |
| Other Neurological Disorders | 0.7067249 | 0.0527387 | -4.65 | 0 | 0.6105627 | 0.8180325 |
| Chronic Pulmonary Disease | 10.78528 | 0.6892894 | 37.21 | 0 | 9.515486 | 12.22452 |
| Diabetes Uncomplicated | 38.94945 | 3.377112 | 42.24 | 0 | 32.8623 | 46.16414 |
| Diabetes Complicated | 3.049772 | 0.1870251 | 18.18 | 0 | 2.704382 | 3.439273 |
| Hypothyroidism | 70.52096 | 7.099106 | 42.28 | 0 | 57.89361 | 85.9025 |
| Renal Failure | 9.200586 | 0.7080732 | 28.84 | 0 | 7.912385 | 10.69852 |
| Liver Disease | 2.646365 | 0.210432 | 12.24 | 0 | 2.264459 | 3.092681 |
| Peptic Ulcer Disease without Bleeding | 1 | (omitted) |  |  |  |  |
| Rheumatoid Arthritis/Collagen Vascular Disease | 1.59725 | 0.1559829 | 4.8 | 0 | 1.319006 | 1.934188 |
| Coagulopathy | 10.50569 | 0.6714709 | 36.8 | 0 | 9.268724 | 11.90773 |
| Obesity | 3.44444 | 0.2027645 | 21.01 | 0 | 3.069098 | 3.865685 |
| Weight Loss | 100.3143 | 7.982471 | 57.91 | 0 | 85.82798 | 117.2457 |
| Fluid and Electrolyte Disorders | 0.7370254 | 0.0423445 | -5.31 | 0 | 0.658534 | 0.8248723 |
| Hypomagnesemia | 0.0051771 | 0.0010872 | -25.06 | 0 | 0.0034302 | 0.0078134 |
| Blood Loss Anemia | 1 | (omitted) |  |  |  |  |
| Deficiency Anemia | 0.0024682 | 0.000782 | -18.95 | 0 | 0.0013264 | 0.0045928 |
| Alcohol Abuse | 21.05258 | 2.799077 | 22.92 | 0 | 16.22305 | 27.31984 |
| Tobacco Use Disorder | 0.0647903 | 0.0067816 | -26.14 | 0 | 0.0527733 | 0.0795437 |
| Psychoses | 0.8882633 | 0.2018608 | -0.52 | 0.602 | 0.5689883 | 1.386692 |
| Depression | 0.0646462 | 0.005907 | -29.97 | 0 | 0.0540461 | 0.0773253 |
| Hypertension Uncomplicated | 2.155634 | 0.1441913 | 11.48 | 0 | 1.890766 | 2.457606 |
| Hypertension Complicated | 1.371704 | 0.1200683 | 3.61 | 0 | 1.155454 | 1.628425 |
| Coronary Artery Disease | 0.1641975 | 0.0101822 | -29.13 | 0 | 0.1454058 | 0.1854178 |
| Aspirin | 6.732738 | 0.513599 | 25 | 0 | 5.797741 | 7.818521 |
| Long Term Anticoagulation | 0.0281817 | 0.0035076 | -28.68 | 0 | 0.0220812 | 0.0359676 |
| Long Term NSAID | 0.0011902 | 0.0001639 | -48.91 | 0 | 0.0009087 | 0.0015589 |
| Long Term Steroid | 33.52 | 4.74508 | 24.81 | 0 | 25.39851 | 44.23844 |
| Vitamin D Deficiency | 0.1957612 | 0.0197162 | -16.19 | 0 | 0.1606932 | 0.238482 |
| History of Bariatric Surgery | 5.237091 | 6.82257 | 1.27 | 0.204 | 0.4075638 | 67.2953 |
| Acute Kidney Injury (AKI) | 27.8556 | 1.861625 | 49.78 | 0 | 24.43575 | 31.75407 |
| Vasopressor Use | 7.527948 | 0.7362585 | 20.64 | 0 | 6.214787 | 9.118575 |
| Mechanical Ventilation | 64570.76 | 7825.632 | 91.39 | 0 | 50918.4 | 81883.61 |
| Remdesivir Administration | 4.305111 | 0.292616 | 21.48 | 0 | 3.768154 | 4.918584 |
| ELIXSUM MEDIAN≥4 | 0.0159317 | 0.0016536 | -39.88 | 0 | 0.0129992 | 0.0195259 |
| _cons | 0.0146406 | 0.0042842 | -14.43 | 0 | 0.0082505 | 0.02598 |
| Note: P>\|t\|=0: p≤0.001  #: Interaction Term | | | | | | |

| **TABLE 12: Baseline Characteristics after Propensity Score Matching Among Covid-19 Hospitalizations with and without Active Cannabis Use for Secondary Outcome: Acute Pulmonary Embolism** | | | | |
| --- | --- | --- | --- | --- |
|  | **Mean (Std. Err.)** | | **t-test** | |
| **Variable:** | **ACU  (N= 2,382)** | **No ACU (N=275,200)** | **t** | **p>\| t \|** |
| **Outcome:** |  |  |  |  |
| **Acute Pulmonary Embolism (PE)** | 0.01427(0.002) | 0.02267(0.003) | -2.15 | 0.031 |
| \| |  |  |  |  |
| **Age in years at admission, y** | 40.733(0.33)* | 42.255(0.32)* | -3.31 | 0.001 |
| **Age group (Continuous variable 1-6)** | 2.0063(0.02) | 1.9878(0.02) | 0.69 | 0.488 |
| **Length of Stay in Days** | 6.9353 (0.26) | 6.2477 (0.16) | 2.29 | 0.022 |
| **LENGTH OF STAY Group** |  |  |  |  |
| **Less than 7 Days** | 0.70571(0.01) | 0.72418 (0.01) | -1.41 | 0.158 |
| **7-13 Days** | 0.18682(0.01) | 0.17968(0.01) | 0.64 | 0.524 |
| **14-20 Days** | 0.05164(0.005) | 0.04282(0.004) | 1.43 | 0.152 |
| **21-29 Days** | 0.03107(0.004) | 0.02939(0.003) | 0.34 | 0.735 |
| **30-45 Days** | 0.01217(0.002) | 0.01763(0.003) | -1.55 | 0.12 |
| **45-60 Days** | 0.0063(0.002) | 0.00378(0.001) | 1.23 | 0.22 |
| **61-780 Days** | 0.0063(0.002) | 0.00252(0.001) | 1.97 | 0.049 |
| **Total Charges in US Dollars, $** | 71,652 (2937.96) | 68,384 (3256.90) | 0.75 | 0.456 |
| **\|** |  |  |  |  |
| **AGE GROUP 1** | 0.32032 (0.01) | 0.322 (0.01) | -0.12 | 0.901 |
| **AGE GROUP 2** | 0.44878 (0.01) | 0.45928 (0.01) | -0.73 | 0.467 |
| **AGE GROUP 3** | 0.14442 (0.01) | 0.13518(0.01) | 0.92 | 0.358 |
| **AGE GROUP 4** | 0.07893(0.01) | 0.07725(0.01) | 0.22 | 0.829 |
| **AGE GROUP 5** | 0.00588(0.002) | 0.00504(0.001) | 0.39 | 0.694 |
| **AGE GROUP 6** | 0.00168(0.001) | 0.00126(0.001) | 0.38 | 0.705 |
| **Racial or Ethnic Group 1: White** | 0.39379(0.01) | 0.38749(0.01) | 0.45 | 0.656 |
| **Racial or Ethnic Group 2: Black/African American** | 0.36314(0.01) | 0.38119(0.01) | -1.29 | 0.198 |
| **Racial or Ethnic Group 3: Hispanic** | 0.16541(0.01) | 0.15743(0.01) | 0.75 | 0.454 |
| **Racial or Ethnic Group 4: Pacific Islander** | 0.01427(0.002) | 0.01637(0.003) | -0.59 | 0.555 |
| **Racial or Ethnic Group 5: Native American** | 0.02729(0.003) | 0.02477(0.003) | 0.55 | 0.585 |
| **Racial or Ethnic Group 6: Other** | 0.0361(0.004) | 0.03275(0.004) | 0.64 | 0.525 |
| **FEMALE** | 0.34677(0.01) | 0.35264(0.01) | -0.43 | 0.671 |
| **Primary Payor: Medicare** | 0.18556(0.01) | 0.1885(0.01) | -0.26 | 0.795 |
| **Primary Payor: Medicaid** | 0.42485(0.01) | 0.41562(0.01) | 0.65 | 0.519 |
| **Primary Payor: Private Insurance** | 0.233(0.01) | 0.22796(0.01) | 0.41 | 0.68 |
| **Primary Payor: Self** | 0.09278(0.01) | 0.10243(0.01) | -1.12 | 0.262 |
| **Primary Payor: No Charge** | 0.00588(0.002) | 0.00798(0.002) | -0.87 | 0.383 |
| **Primary Payor: Other** | 0.05793(0.01) | 0.05751(0.01) | 0.06 | 0.95 |
| **HOSPITAL BEDSIZE 1: Small** | 0.21914(0.01) | 0.21704(0.01) | 0.18 | 0.861 |
| **HOSPITAL BEDSIZE 2: Medium** | 0.27288(0.01) | 0.27834(0.01) | -0.42 | 0.673 |
| **HOSPITAL BEDSIZE 3: Large** | 0.50798(0.01) | 0.50462(0.01) | 0.23 | 0.817 |
| **HOSPITAL LOCTEACH 1: Rural** | 0.04114(0.004) | 0.03904(0.004) | 0.37 | 0.712 |
| **HOSPITAL LOCTEACH 2: Urban Nonteaching** | 0.144(0.01) | 0.14232(0.01) | 0.17 | 0.869 |
| **HOSPITAL LOCTEACH 3: Urban Teaching** | 0.81486(0.01) | 0.81864(0.01) | -0.34 | 0.736 |
| **HOSPITAL REGION 1: Northeast** | 0.17632(0.01) | 0.17338(0.01) | 0.27 | 0.79 |
| **HOSPITAL REGION 2: Midwest** | 0.24433(0.01) | 0.22922(0.01) | 1.23 | 0.22 |
| **HOSPITAL REGION 3: South** | 0.35978(0.01) | 0.3707(0.01) | -0.78 | 0.434 |
| **HOSPITAL REGION 4: West** | 0.21956(0.01) | 0.2267(0.01) | -0.59 | 0.554 |
| **ZIPINC_QRTL1** | 0.42569(0.01) | 0.42024(0.01) | 0.38 | 0.703 |
| **ZIPINC_QRTL2** | 0.25105(0.01) | 0.25525(0.01) | -0.33 | 0.739 |
| **ZIPINC_QRTL3** | 0.19186(0.01) | 0.19186(0.01) | 0 | 1 |
| **ZIPINC_QRTL4** | 0.1314(0.01) | 0.13266(0.01) | -0.13 | 0.898 |
| **ADMISSION ON THE WEEKEND** | 0.26994(0.01) | 0.26406(0.01) | 0.46 | 0.647 |
| **Admission Month 1: January** | 0.0042(0.001) | 0.00462(0.001) | -0.22 | 0.827 |
| **Admission Month 2: February** | 0.00462(0.001) | 0.00462(0.001) | 0 | 1 |
| **Admission Month 3: March** | 0.02939(0.003) | 0.03023(0.004) | -0.17 | 0.865 |
| **Admission Month 4: April** | 0.07473(0.01) | 0.07599(0.01) | -0.16 | 0.869 |
| **Admission Month 5: May** | 0.06591(0.01) | 0.05122(0.01) | 2.16 | 0.031 |
| **Admission Month 6: June** | 0.07557(0.01) | 0.09614(0.01) | -2.54 | 0.011 |
| **Admission Month 7: July** | 0.13056(0.01) | 0.13476(0.01) | -0.43 | 0.669 |
| **Admission Month 8: August** | 0.0974(0.01) | 0.09278(0.01) | 0.54 | 0.587 |
| **Admission Month 9: September** | 0.06801(0.01) | 0.05877(0.01) | 1.31 | 0.191 |
| **Admission Month 10: October** | 0.08564(0.01) | 0.08396(0.01) | 0.21 | 0.835 |
| **Admission Month 11: November** | 0.17045(0.01) | 0.17086(0.01) | -0.04 | 0.969 |
| **Admission Month 12: December** | 0.19353(0.01) | 0.19605(0.01) | -0.22 | 0.826 |
| **ELIXSUM MEDIAN≥4** | 0.49748(0.01) | 0.48699(0.01) | 0.72 | 0.469 |
| **Congestive Heart Failure** | 0.10663(0.01) | 0.11125(0.01) | -0.51 | 0.609 |
| **Cardiac Arrhythmias** | 0.13644(0.01) | 0.12427(0.01) | 1.25 | 0.212 |
| **Valvular Disease** | 0.02183(0.003) | 0.02141(0.003) | 0.1 | 0.921 |
| **Peripheral Vascular Disorders** | 0.02687(0.003) | 0.02729(0.003) | -0.09 | 0.929 |
| **Hypertension Uncomplicated** | 0.36566(0.01) | 0.37112(0.01) | -0.39 | 0.696 |
| **Paralysis** | 0.01805(0.003) | 0.02057(0.003) | -0.63 | 0.528 |
| **Other Neurological Disorders** | 0.13476(0.01) | 0.1356(0.01) | -0.08 | 0.932 |
| **Chronic Pulmonary Disease** | 0.2204(0.01) | 0.22754(0.01) | -0.59 | 0.555 |
| **Diabetes Uncomplicated** | 0.08732(0.01) | 0.0806(0.01) | 0.84 | 0.403 |
| **Diabetes Complicated** | 0.15071(0.01) | 0.14861(0.01) | 0.2 | 0.839 |
| **Hypothyroidism** | 0.03904(0.004) | 0.03568(0.004) | 0.61 | 0.541 |
| **Renal Failure** | 0.10705(0.01) | 0.0995(0.01) | 0.86 | 0.392 |
| **Liver Disease** | 0.08144(0.01) | 0.09446(0.01) | -1.59 | 0.113 |
| **Peptic Ulcer Disease without Bleeding** | 0.0063(0.002) | 0.0042(0.001) | 1 | 0.316 |
| **Rheumatoid Arthritis Collagen Vascular Disorder** | 0.01763(0.003) | 0.02267(0.003) | -1.24 | 0.216 |
| **Coagulopathy** | 0.08354(0.01) | 0.07431(0.01) | 1.18 | 0.237 |
| **Obesity** | 0.20865(0.01) | 0.21704(0.01) | -0.71 | 0.479 |
| **Weight Loss** | 0.04534(0.004) | 0.04408(0.004) | 0.21 | 0.833 |
| **Fluid and Electrolyte Disorders** | 0.39337(0.01) | 0.39505(0.01) | -0.12 | 0.906 |
| **Blood Loss Anemia** | 0.00252(0.001) | 0.00336(0.001) | -0.54 | 0.593 |
| **Deficiency Anemia** | 0.03988(0.004) | 0.0403(0.004) | -0.07 | 0.941 |
| **Alcohol Abuse** | 0.17506(0.01) | 0.16919(0.01) | 0.54 | 0.591 |
| **Psychoses** | 0.14316(0.01) | 0.13266(0.01) | 1.05 | 0.294 |
| **Depression** | 0.20571(0.01) | 0.20319(0.01) | 0.22 | 0.829 |
| **Hypertension Complicated** | 0.144(0.01) | 0.14022(0.01) | 0.37 | 0.709 |
| **Aspirin** | 0.07137(0.01) | 0.06339(0.01) | 1.1 | 0.272 |
| **Long Term Anticoagulation** | 0.01259(0.004) | 0.01301(0.004) | -0.13 | 0.897 |
| **Long Term NSAID** | 0.01175(0.002) | 0.00882(0.002) | 1.01 | 0.315 |
| **Long Term Steroid** | 0.3283(0.002) | 0.32242(0.002) | 0.43 | 0.665 |
| **Tobacco Use Disorder** | 0.07431(0.01) | 0.07179(0.01) | 0.33 | 0.738 |
| **Coronary Artery Disease** | 0.06927(0.01) | 0.07137(0.01) | -0.28 | 0.777 |
| **Acute Kidney Injury** | 0.20949(0.01) | 0.20445(0.01) | 0.43 | 0.668 |
| **Remdesivir Administration** | 0.09068(0.01) | 0.0869(0.01) | 0.46 | 0.647 |
| **Vasopressor Use** | 0.01301(0.002) | 0.01385(0.002) | -0.25 | 0.801 |
| **Hypomagnesemia** | 0.04744(0.004) | 0.055(0.01) | -1.18 | 0.237 |
| **Vitamin D Deficiency** | 0.02225(0.003) | 0.02225(0.003) | 0 | 1 |
| **History of Bariatric Surgery** | 0.00546(0.002) | 0.00546(0.002) | 0 | 1 |
| Note: P>\|t\|=0: p≤0.001  *: Sample was matched for utilizing Age Group not Age | | | | |

**Supplementary TABLE 13: Propensity Score Matched Sample Logistic Regression for Secondary Outcome Acute Pulmonary Embolism among Covid-19 Disease Hospitalizations** **January to December 2020**

| Log likelihood = -9641.0983 | Number of obs = 265,559  LR chi2(73) = 18878.15  Prob > chi2 = 0.0000  Pseudo R2 = 0.4947 | | | | | |
| --- | --- | --- | --- | --- | --- | --- |
| **Dependent Variable:**  Acute Pulmonary Embolism | Odds Ratio | Linearized Std. Err. | t | P>\|t\| | [95% Confidence Interval | |
|  |  |  |  |  | Lower Bound | Upper Bound |

| ACU | 0.5714432 | 0.1119591 | -2.86 | 0.004 | 0.3892268 | 0.838964 |
| --- | --- | --- | --- | --- | --- | --- |
| \| |  |  |  |  |  |  |
| Length of Stay in days (Comparison group 0-6 days) |  |  |  |  |  |  |
| 7-13 days | 1.967632 | 0.1632576 | 8.16 | 0 | 1.672316 | 2.315098 |
| 14-20 days | 23.61402 | 2.244874 | 33.26 | 0 | 19.59973 | 28.45048 |
| 21-29 days | 11.47321 | 1.300784 | 21.52 | 0 | 9.187117 | 14.32816 |
| 30-45 days | 24.64008 | 3.659862 | 21.57 | 0 | 18.41665 | 32.96655 |
| 45-60 days | 0.1556454 | 0.1579269 | -1.83 | 0.067 | 0.0213038 | 1.137143 |
| 61-780 days | 1 | (empty) |  |  |  |  |
| \| |  |  |  |  |  |  |
| AGE in years, y (Comparison group 18-29y) |  |  |  |  |  |  |
| 30-54 years old | 20.23508 | 4.226073 | 14.4 | 0 | 13.43794 | 30.47035 |
| 55-64 years old | 14.18205 | 3.047168 | 12.34 | 0 | 9.307845 | 21.60872 |
| 65-79 years old | 13.18693 | 3.016529 | 11.28 | 0 | 8.422284 | 20.64701 |
| 80-85 years old | 301.3902 | 81.68389 | 21.06 | 0 | 177.1876 | 512.6547 |
| 85-120 years old | 1 | (empty) |  |  |  |  |
| \| |  |  |  |  |  |  |
| Racial or Ethnic Group (Comparison group White race) |  |  |  |  |  |  |
| Black | 4.591587 | 0.3351537 | 20.88 | 0 | 3.979524 | 5.297788 |
| Hispanic | 0.5623174 | 0.0561828 | -5.76 | 0 | 0.4623123 | 0.683955 |
| Asian/Pacific Islander | 8.754217 | 1.539737 | 12.33 | 0 | 6.201598 | 12.35751 |
| Native American | 180.1136 | 20.36596 | 45.93 | 0 | 144.3107 | 224.7989 |
| Other Racial or Ethnic Group | 14.85633 | 1.575641 | 25.44 | 0 | 12.06798 | 18.28895 |
| \| |  |  |  |  |  |  |
| FEMALE (Comparison group Male Sex) | 0.2347939 | 0.0153918 | -22.1 | 0 | 0.2064842 | 0.266985 |
| \| |  |  |  |  |  |  |
| Primary Payor (Comparison group Medicare) |  |  |  |  |  |  |
| Medicaid | 1.840408 | 0.1726352 | 6.5 | 0 | 1.531331 | 2.211867 |
| Private | 4.245674 | 0.4164049 | 14.74 | 0 | 3.503184 | 5.145532 |
| Self-pay | 1.52693 | 0.2598871 | 2.49 | 0.013 | 1.093811 | 2.13155 |
| No Charge | 6.54293 | 4.743244 | 2.59 | 0.01 | 1.580158 | 27.09218 |
| Other | 7.258877 | 1.053616 | 13.66 | 0 | 5.461584 | 9.64762 |
| \| |  |  |  |  |  |  |
| HOSPITAL BEDSIZE (Comparison group small bed size) |  |  |  |  |  |  |
| Medium | 1.454858 | 0.1228007 | 4.44 | 0 | 1.233028 | 1.716596 |
| Large | 0.5583477 | 0.0454461 | -7.16 | 0 | 0.4760166 | 0.6549187 |
| \| |  |  |  |  |  |  |
| HOSPITAL LOCATION/TEACH STATUS (Comparison group Rural) |  |  |  |  |  |  |
| Urban | 0.4843685 | 0.0427283 | -8.22 | 0 | 0.4074625 | 0.5757901 |
| Urban | 0.1350548 | 0.0104593 | -25.85 | 0 | 0.1160349 | 0.1571923 |
| \| |  |  |  |  |  |  |
| HOSPITAL REGION (Comparison group Northeast) |  |  |  |  |  |  |
| Midwest | 0.635713 | 0.0638888 | -4.51 | 0 | 0.5220545 | 0.7741165 |
| South | 0.5924777 | 0.0616602 | -5.03 | 0 | 0.4831545 | 0.7265374 |
| West | 1.783949 | 0.1788594 | 5.77 | 0 | 1.465685 | 2.171322 |
| \| |  |  |  |  |  |  |
| ZIPINC_QRTL (Comparison group ZIPINC_QRTL1) |  |  |  |  |  |  |
| 2 | 11.5073 | 0.872862 | 32.21 | 0 | 9.917613 | 13.35179 |
| 3 | 2.83899 | 0.2486533 | 11.91 | 0 | 2.391174 | 3.370671 |
| 4 | 0.3719932 | 0.0487484 | -7.55 | 0 | 0.2877319 | 0.4809301 |
| \| |  |  |  |  |  |  |
| ADMISSION ON THE WEEKEND | 3.998571 | 0.2152444 | 25.75 | 0 | 3.598192 | 4.443501 |
| Admission Month: April | 0.9943103 | 0.108923 | -0.05 | 0.958 | 0.8021876 | 1.232446 |
| Congestive Heart Failure | 3.3939 | 0.4363169 | 9.51 | 0 | 2.637966 | 4.366453 |
| Cardiac Arrhythmias | 6.190984 | 0.394755 | 28.59 | 0 | 5.463672 | 7.015114 |
| Valvular Disease | 0.0310863 | 0.0185144 | -5.83 | 0 | 0.0096741 | 0.0998917 |
| Peripheral Vascular Disorders | 5.993714 | 0.6275319 | 17.1 | 0 | 4.881762 | 7.358942 |
| Paralysis | 0.2081339 | 0.0656191 | -4.98 | 0 | 0.112197 | 0.3861038 |
| Other Neurological Disorders | 0.3785059 | 0.0284019 | -12.95 | 0 | 0.3267391 | 0.4384743 |
| Chronic Pulmonary Disease | 1.742241 | 0.1273558 | 7.59 | 0 | 1.509685 | 2.010621 |
| Diabetes Uncomplicated | 5.683789 | 0.3923939 | 25.17 | 0 | 4.964474 | 6.507327 |
| Diabetes Complicated | 0.3753587 | 0.026309 | -13.98 | 0 | 0.3271791 | 0.4306331 |
| Hypothyroidism | 4.35E+00 | 3.77E-01 | 17 | 0 | 3.67E+00 | 5.157938 |
| Renal Failure | 0.1054255 | 0.0146007 | -16.24 | 0 | 0.0803637 | 0.1383029 |
| Liver Disease | 0.3926581 | 0.0453903 | -8.09 | 0 | 0.3130529 | 0.4925059 |
| Peptic Ulcer Disease without Bleeding | 1.255764 | 1.276703 | 0.22 | 0.823 | 0.1712037 | 9.210913 |
| Rheumatoid Arthritis/Collagen Vascular Disease | 7.61E-06 | 7.81E-06 | -11.49 | 0 | 1.02E-06 | 0.0000569 |
| Coagulopathy | 3.536069 | 0.2606331 | 17.14 | 0 | 3.060421 | 4.085642 |
| Obesity | 11.22671 | 0.7711337 | 35.21 | 0 | 9.812635 | 12.84456 |
| Weight Loss | 0.1678565 | 0.0252374 | -11.87 | 0 | 0.1250141 | 0.2253809 |
| Fluid and Electrolyte Disorders | 4.232015 | 0.2612863 | 23.37 | 0 | 3.749676 | 4.776401 |
| Hypomagnesemia | 2.213886 | 0.2856161 | 6.16 | 0 | 1.719257 | 2.85082 |
| Blood Loss Anemia | 0.1943015 | 0.2023659 | -1.57 | 0.116 | 0.0252316 | 1.496258 |
| Deficiency Anemia | 0.1674016 | 0.0299534 | -9.99 | 0 | 0.1178835 | 0.2377204 |
| Alcohol Abuse | 0.0963089 | 0.0157121 | -14.34 | 0 | 0.0699519 | 0.132597 |
| Tobacco Use Disorder | 0.2666696 | 0.0355942 | -9.9 | 0 | 0.2052853 | 0.3464089 |
| Hypertension Complicated | 0.2594427 | 0.0384054 | -9.11 | 0 | 0.1941054 | 0.3467729 |
| Schizophrenia | 0.788249 | 0.3230383 | -0.58 | 0.562 | 0.3530417 | 1.759952 |
| Coronary Artery Disease | 4.844336 | 0.4018926 | 19.02 | 0 | 4.117347 | 5.699688 |
| Aspirin | 0.3814111 | 0.0363412 | -10.12 | 0 | 0.316439 | 0.4597235 |
| Vitamin D Deficiency | 0.0077184 | 0.0079745 | -4.71 | 0 | 0.0010188 | 0.0584753 |
| Acute Kidney Injury (AKI) | 0.9984341 | 0.0779017 | -0.02 | 0.984 | 0.8568511 | 1.163412 |
| Vasopressor Use | 0.0543277 | 0.0100551 | -15.74 | 0 | 0.0377988 | 0.0780843 |
| Mechanical Ventilation | 0.4845714 | 0.0473018 | -7.42 | 0 | 0.4001908 | 0.5867438 |
| Remdesivir Administration | 0.2360025 | 0.019082 | -17.86 | 0 | 0.2014153 | 0.2765289 |
| _cons | 0.0000307 | 0.0000105 | -30.41 | 0 | 0.0000157 | 0.00006 |
| Note: P>\|t\|=0: p≤0.001  Note: 61-780 days not= 0 predicts failure perfectly;  61-780 days omitted and 21 obs not used.  Note: 85-120 years old not= 0 predicts failure perfectly;  85-120 years old omitted and 7 obs not used. Note: 7412 failures and 0 successes completely determined. | | | | | | |

| **TABLE 14: Baseline Characteristics after Propensity Score Matching Among Covid-19 Hospitalizations with and without Active Cannabis Use for Secondary Outcome: Mechanical Ventilation** | | | | |
| --- | --- | --- | --- | --- |
|  | **Mean (Std. Err.)** | | **t-test** | |
| **Variable:** | **ACU (N= 2,382)** | **No ACU (N=281,051)** | **t** | **p>\| t \|** |
| **Outcomes:** |  |  |  |  |
| **Mechanical Vent** | 0.06927(0.01) | 0.0911(0.01) | -2.78 | 0.006 |
| \| |  |  |  |  |
| **Age in years at admission, y** | 40.733(0.33) * | 42.194(0.32) * | -3.18 | 0.001 |
| **Age group (Continuous variable 1-6)** | 2.0063(0.02) | 1.9958(0.02) | 0.39 | 0.693 |
| **Length of Stay in Days** | 6.9353(0.26) | 7.2254(0.23) | -0.84 | 0.399 |
| **Total Charges in US Dollars, $** | $71,652(2937.96) | $83,809(5323.83) | -2 | 0.046 |
| **\|** |  |  |  |  |
| **AGE GROUP 1** | 0.32032(0.01) | 0.3178(0.01) | 0.19 | 0.852 |
| **AGE GROUP 2** | 0.44878(0.01) | 0.45214(0.01) | -0.23 | 0.816 |
| **AGE GROUP 3** | 0.14442(0.01) | 0.15617(0.01) | -1.14 | 0.256 |
| **AGE GROUP 4** | 0.07893(0.01) | 0.06675(0.01) | 1.62 | 0.106 |
| **AGE GROUP 5** | 0.00588(0.002) | 0.00462(0.001) | 0.6 | 0.548 |
| **AGE GROUP 6** | 0.00168(0.001) | 0.00252(0.001) | -0.63 | 0.527 |
| **Racial or Ethnic Group 1: White** | 0.39379(0.01) | 0.39715(0.01) | -0.24 | 0.813 |
| **Racial or Ethnic Group 2: Black/African American** | 0.36314(0.01) | 0.37238(0.01) | -0.66 | 0.509 |
| **Racial or Ethnic Group 3: Hispanic** | 0.16541(0.01) | 0.14232(0.01) | 2.21 | 0.027 |
| **Racial or Ethnic Group 4: Pacific Islander** | 0.01427(0.002) | 0.01175(0.002) | 0.77 | 0.443 |
| **Racial or Ethnic Group 5: Native American** | 0.02729(0.003) | 0.0382(0.004) | -2.12 | 0.034 |
| **Racial or Ethnic Group 6: Other** | 0.0361(0.004) | 0.0382(0.004) | -0.38 | 0.702 |
| **FEMALE** | 0.34677(0.01) | 0.35516(0.01) | -0.61 | 0.544 |
| **Primary Payor: Medicare** | 0.18556(0.01) | 0.17842(0.01) | 0.64 | 0.523 |
| **Primary Payor: Medicaid** | 0.42485(0.01) | 0.42149(0.01) | 0.23 | 0.815 |
| **Primary Payor: Private Insurance** | 0.233(0.01) | 0.24349(0.01) | -0.85 | 0.395 |
| **Primary Payor: Self** | 0.09278(0.01) | 0.09698(0.01) | -0.49 | 0.621 |
| **Primary Payor: No Charge** | 0.00588(0.002) | 0.00462(0.001) | 0.6 | 0.548 |
| **Primary Payor: Other** | 0.05793(0.01) | 0.055(0.01) | 0.44 | 0.66 |
| **HOSPITAL BEDSIZE 1: Small** | 0.21914(0.01) | 0.22628(0.01) | -0.59 | 0.554 |
| **HOSPITAL BEDSIZE 2: Medium** | 0.27288(0.01) | 0.28338(0.01) | -0.81 | 0.419 |
| **HOSPITAL BEDSIZE 3: Large** | 0.50798(0.01) | 0.49034(0.01) | 1.22 | 0.224 |
| **HOSPITAL LOCTEACH 1: Rural** | 0.04114(0.004) | 0.04954(0.004) | -1.39 | 0.164 |
| **HOSPITAL LOCTEACH 2: Urban Nonteaching** | 0.144(0.01) | 0.13686(0.01) | 0.71 | 0.478 |
| **HOSPITAL LOCTEACH 3: Urban Teaching** | 0.81486(0.01) | 0.8136(0.01) | 0.11 | 0.911 |
| **HOSPITAL REGION 1: Northeast** | 0.17632(0.01) | 0.17716(0.01) | -0.08 | 0.939 |
| **HOSPITAL REGION 2: Midwest** | 0.24433(0.01) | 0.24223(0.01) | 0.17 | 0.866 |
| **HOSPITAL REGION 3: South** | 0.35978(0.01) | 0.36272(0.01) | -0.21 | 0.833 |
| **HOSPITAL REGION 4: West** | 0.21956(0.01) | 0.21788(0.01) | 0.14 | 0.889 |
| **ZIPINC_QRTL1** | 0.42569(0.01) | 0.43997(0.01) | -0.99 | 0.32 |
| **ZIPINC_QRTL2** | 0.25105(0.01) | 0.25105(0.01) | 0 | 1 |
| **ZIPINC_QRTL3** | 0.19186(0.01) | 0.18178(0.01) | 0.89 | 0.372 |
| **ZIPINC_QRTL4** | 0.1314(0.01) | 0.1272(0.01) | 0.43 | 0.666 |
| **ADMISSION ON THE WEEKEND** | 0.26994(0.01) | 0.28338(0.01) | -1.04 | 0.3 |
| **Admission Month 1: January** | 0.0042(0.001) | 0.00588(0.002) | -0.82 | 0.413 |
| **Admission Month 2: February** | 0.00462(0.001) | 0.00462(0.001) | 0 | 1 |
| **Admission Month 3: March** | 0.02939(0.003) | 0.03191(0.004) | -0.5 | 0.614 |
| **Admission Month 4: April** | 0.07473(0.01) | 0.08102(0.01) | -0.81 | 0.417 |
| **Admission Month 5: May** | 0.06591(0.01) | 0.06549(0.01) | 0.06 | 0.953 |
| **Admission Month 6: June** | 0.07557(0.01) | 0.07809(0.01) | -0.33 | 0.744 |
| **Admission Month 7: July** | 0.13056(0.01) | 0.12259(0.01) | 0.83 | 0.408 |
| **Admission Month 8: August** | 0.0974(0.01) | 0.09824(0.01) | -0.1 | 0.922 |
| **Admission Month 9: September** | 0.06801(0.01) | 0.06759(0.01) | 0.06 | 0.954 |
| **Admission Month 10: October** | 0.08564(0.01) | 0.08396(0.01) | 0.21 | 0.835 |
| **Admission Month 11: November** | 0.17045(0.01) | 0.15575(0.01) | 1.37 | 0.17 |
| **Admission Month 12: December** | 0.19353(0.01) | 0.20487(0.01) | -0.98 | 0.327 |
| **ELIXSUM MEDIAN≥4** | 0.49748(0.01) | 0.51385(0.01) | -1.13 | 0.259 |
| **Congestive Heart Failure** | 0.10663(0.01) | 0.10957(0.01) | -0.33 | 0.744 |
| **Cardiac Arrhythmias** | 0.13644(0.01) | 0.14694(0.01) | -1.04 | 0.299 |
| **Valvular Disease** | 0.02183(0.003) | 0.02309(0.003) | -0.29 | 0.769 |
| **Pulmonary Circulation Disorders** | 0.03442(0.004) | 0.03442(0.004) | 0 | 1 |
| **Peripheral Vascular Disorders** | 0.02687(0.003) | 0.02813(0.003) | -0.27 | 0.79 |
| **Hypertension Uncomplicated** | 0.36566(0.01) | 0.36902(0.01) | -0.24 | 0.81 |
| **Paralysis** | 0.01805(0.003) | 0.01847(0.003) | -0.11 | 0.914 |
| **Other Neurological Disorders** | 0.13476(0.01) | 0.14148(0.01) | -0.67 | 0.502 |
| **Chronic Pulmonary Disease** | 0.2204(0.01) | 0.2246(0.01) | -0.35 | 0.728 |
| **Diabetes Uncomplicated** | 0.08732(0.01) | 0.08984(0.01) | -0.31 | 0.76 |
| **Diabetes Complicated** | 0.15071(0.01) | 0.15743(0.01) | -0.64 | 0.521 |
| **Hypothyroidism** | 0.03904(0.004) | 0.04072(0.004) | -0.3 | 0.767 |
| **Renal Failure** | 0.10705(0.01) | 0.10076(0.01) | 0.71 | 0.476 |
| **Liver Disease** | 0.08144(0.01) | 0.07725(0.01) | 0.54 | 0.592 |
| **Peptic Ulcer Disease without Bleeding** | 0.0063(0.002) | 0.00504(0.001) | 0.58 | 0.563 |
| **Rheumatoid Arthritis Collagen Vascular Disorder** | 0.01763(0.003) | 0.01679(0.003) | 0.22 | 0.824 |
| **Coagulopathy** | 0.08354(0.01) | 0.10076(0.01) | -2.05 | 0.04 |
| **Obesity** | 0.20865(0.01) | 0.21914(0.01) | -0.88 | 0.377 |
| **Weight Loss** | 0.04534(0.004) | 0.05542(0.01) | -1.59 | 0.112 |
| **Fluid and Electrolyte Disorders** | 0.39337(0.01) | 0.40386(0.01) | -0.74 | 0.46 |
| **Blood Loss Anemia** | 0.00252(0.001) | 0.00168(0.001) | 0.63 | 0.527 |
| **Deficiency Anemia** | 0.03988(0.004) | 0.04366(0.004) | -0.65 | 0.515 |
| **Alcohol Abuse** | 0.17506(0.01) | 0.18766(0.01) | -1.13 | 0.259 |
| **Psychoses** | 0.14316(0.01) | 0.14064(0.01) | 0.25 | 0.803 |
| **Depression** | 0.20571(0.01) | 0.19228(0.01) | 1.16 | 0.246 |
| **Hypertension Complicated** | 0.144(0.01) | 0.14232(0.01) | 0.17 | 0.869 |
| **Aspirin** | 0.07137(0.01) | 0.07389(0.01) | -0.33 | 0.738 |
| **Long Term Anticoagulation** | 0.0424(0.004) | 0.04198(0.004) | 0.07 | 0.943 |
| **Long Term NSAID** | 0.01259(0.002) | 0.01134(0.002) | 0.4 | 0.689 |
| **Long Term Steroid** | 0.01175(0.002) | 0.0105(0.002) | 0.41 | 0.679 |
| **Tobacco Use Disorder** | 0.3283(0.01) | 0.31822(0.01) | 0.74 | 0.457 |
| **Coronary Artery Disease** | 0.07431(0.01) | 0.07305(0.01) | 0.17 | 0.868 |
| **Acute Kidney Injury** | 0.20949(0.01) | 0.21243(0.01) | -0.25 | 0.804 |
| **Remdesivir Administration** | 0.09068(0.01) | 0.08186(0.01) | 1.08 | 0.279 |
| **Vasopressor Use** | 0.01301(0.002) | 0.01385(0.002) | -0.25 | 0.801 |
| **Hypomagnesemia** | 0.04744(0.004) | 0.04954(0.004) | -0.34 | 0.736 |
| **Vitamin D Deficiency** | 0.02225(0.003) | 0.02015(0.003) | 0.5 | 0.615 |
| **History of Bariatric Surgery** | 0.00546(0.002) | 0.0063(0.002) | -0.38 | 0.705 |
| Note: P>\|t\|=0: p≤0.001  *: Sample was matched for utilizing Age Group not Age | | | | |

**Supplementary TABLE 15: Propensity Score Matched Sample Logistic Regression for Secondary Outcome Mechanical Ventilation among Covid-19 Disease Hospitalizations** **January to December 2020**

| Log likelihood = -42038.619 | Number of obs = 272,729  LR chi2(73) = 135629.95  Prob > chi2 = 0.0000  Pseudo R2 = 0.6173 | | | | | |
| --- | --- | --- | --- | --- | --- | --- |
| **Dependent Variable:**  Mechanical Ventilation | Odds Ratio | Linearized Std. Err. | t | P>\|t\| | [95% Confidence Interval | |
|  |  |  |  |  | Lower Bound | Upper Bound |

| ACU | 0.8136229 | 0.0824482 | -2.04 | 0.042 | 0.6670631 | 0.9923832 |
| --- | --- | --- | --- | --- | --- | --- |
| \| |  |  |  |  |  |  |
| AGE in years, y (Comparison group 18-29y) |  |  |  |  |  |  |
| 30-54 years old | 1.769944 | 0.0935932 | 10.8 | 0 | 1.595691 | 1.963227 |
| 55-64 years old | 1.603719 | 0.0910086 | 8.32 | 0 | 1.434907 | 1.792391 |
| 65-79 years old | 2.000569 | 0.1177651 | 11.78 | 0 | 1.782571 | 2.245226 |
| 80-85 years old | 1.174499 | 0.1007974 | 1.87 | 0.061 | 0.992661 | 1.389645 |
| 85-120 years old | 1 | (empty) |  |  |  |  |
| \| |  |  |  |  |  |  |
| Racial or Ethnic Group (Comparison group White race) |  |  |  |  |  |  |
| Black | 0.6336706 | 0.0186548 | -15.5 | 0 | 0.5981427 | 0.6713088 |
| Hispanic | 2.492071 | 0.0763714 | 29.8 | 0 | 2.346792 | 2.646343 |
| Asian/Pacific Islander | 1.00467 | 0.1392336 | 0.03 | 0.973 | 0.7656995 | 1.318221 |
| Native American | 5.658924 | 0.3696559 | 26.53 | 0 | 4.978874 | 6.43186 |
| Other Racial or Ethnic Group | 1.232801 | 0.0820816 | 3.14 | 0.002 | 1.081979 | 1.404647 |
| \| |  |  |  |  |  |  |
| FEMALE (Comparison group Male Sex) | 2.557726 | 0.0688224 | 34.9 | 0 | 2.426332 | 2.696236 |
| \| |  |  |  |  |  |  |
| Primary Payor (Comparison group Medicare) |  |  |  |  |  |  |
| Medicaid | 0.6075047 | 0.022669 | -13.36 | 0 | 0.5646601 | 0.6536003 |
| Private | 0.8502282 | 0.0303227 | -4.55 | 0 | 0.7928264 | 0.911786 |
| Self-pay | 0.6665322 | 0.0403785 | -6.7 | 0 | 0.5919097 | 0.7505625 |
| No Charge | 0.6570306 | 0.111786 | -2.47 | 0.014 | 0.4707211 | 0.9170806 |
| Other | 1.605834 | 0.086746 | 8.77 | 0 | 1.444506 | 1.785179 |
| \| |  |  |  |  |  |  |
| HOSPITAL BEDSIZE (Comparison group small bed size) |  |  |  |  |  |  |
| Medium | 1.174339 | 0.0384275 | 4.91 | 0 | 1.101386 | 1.252123 |
| Large | 1.464007 | 0.0437129 | 12.77 | 0 | 1.38079 | 1.552239 |
| \| |  |  |  |  |  |  |
| HOSPITAL LOCATION/TEACH STATUS (Comparison group Rural) |  |  |  |  |  |  |
| Urban | 0.1137886 | 0.0052885 | -46.76 | 0 | 0.1038814 | 0.1246407 |
| Urban | 0.1270641 | 0.0051102 | -51.3 | 0 | 0.1174329 | 0.1374852 |
| \| |  |  |  |  |  |  |
| HOSPITAL REGION (Comparison group Northeast) |  |  |  |  |  |  |
| Midwest | 5.176394 | 0.2360357 | 36.06 | 0 | 4.733842 | 5.660318 |
| South | 5.858474 | 0.2543875 | 40.71 | 0 | 5.38051 | 6.378895 |
| West | 2.530832 | 0.1186124 | 19.81 | 0 | 2.308713 | 2.77432 |
| \| |  |  |  |  |  |  |
| ZIPINC_QRTL (Comparison group ZIPINC_QRTL1) |  |  |  |  |  |  |
| 2 | 0.2941762 | 0.0096437 | -37.32 | 0 | 0.2758694 | 0.3136979 |
| 3 | 0.3684912 | 0.0131852 | -27.9 | 0 | 0.343534 | 0.3952614 |
| 4 | 0.3529731 | 0.0137762 | -26.68 | 0 | 0.3269791 | 0.3810336 |
| \| |  |  |  |  |  |  |
| ADMISSION ON THE WEEKEND | 0.7404038 | 0.0198806 | -11.19 | 0 | 0.702446 | 0.7804126 |
| Admission Month: April | 1.748017 | 0.0739984 | 13.19 | 0 | 1.608836 | 1.899238 |
| Congestive Heart Failure | 2.9009 | 0.1449466 | 21.31 | 0 | 2.630278 | 3.199366 |
| Cardiac Arrhythmias | 1.74907 | 0.0469031 | 20.85 | 0 | 1.659516 | 1.843458 |
| Valvular Disease | 0.1648212 | 0.0111004 | -26.77 | 0 | 0.1444396 | 0.1880788 |
| Peripheral Vascular Disorders | 0.7762025 | 0.0464126 | -4.24 | 0 | 0.6903637 | 0.8727144 |
| Paralysis | 52.27188 | 2.800294 | 73.85 | 0 | 47.06172 | 58.05885 |
| Other Neurological Disorders | 8.743032 | 0.2427988 | 78.08 | 0 | 8.279874 | 9.232098 |
| Chronic Pulmonary Disease | 2.370607 | 0.063484 | 32.23 | 0 | 2.24939 | 2.498357 |
| Diabetes Uncomplicated | 0.2061845 | 0.0087835 | -37.06 | 0 | 0.1896682 | 0.2241391 |
| Diabetes Complicated | 1.34704 | 0.0382606 | 10.49 | 0 | 1.2741 | 1.424156 |
| Hypothyroidism | 0.1546021 | 0.0086679 | -33.3 | 0 | 0.1385133 | 0.1725595 |
| Renal Failure | 0.2378552 | 0.0097159 | -35.16 | 0 | 0.2195547 | 0.257681 |
| Liver Disease | 1.639733 | 0.0596704 | 13.59 | 0 | 1.526854 | 1.760956 |
| Peptic Ulcer Disease without Bleeding | 0.0086237 | 0.0039469 | -10.39 | 0 | 0.0035166 | 0.0211481 |
| Rheumatoid Arthritis/Collagen Vascular Disease | 0.3027846 | 0.018987 | -19.05 | 0 | 0.2677668 | 0.342382 |
| Coagulopathy | 1.305948 | 0.0341756 | 10.2 | 0 | 1.240653 | 1.374678 |
| Obesity | 1.405373 | 0.0372201 | 12.85 | 0 | 1.334284 | 1.48025 |
| Weight Loss | 1.71855 | 0.0692984 | 13.43 | 0 | 1.587956 | 1.859883 |
| Fluid and Electrolyte Disorders | 7.18775 | 0.2190512 | 64.72 | 0 | 6.770988 | 7.630164 |
| Hypomagnesemia | 0.3501061 | 0.0180093 | -20.4 | 0 | 0.3165296 | 0.3872444 |
| Blood Loss Anemia | 0.7311473 | 0.1282236 | -1.79 | 0.074 | 0.518474 | 1.031057 |
| Deficiency Anemia | 2.483711 | 0.1102977 | 20.49 | 0 | 2.276672 | 2.709578 |
| Alcohol Abuse | 2.362128 | 0.0994187 | 20.42 | 0 | 2.175092 | 2.565248 |
| Tobacco Use Disorder | 0.556956 | 0.0248225 | -13.13 | 0 | 0.5103691 | 0.6077954 |
| Psychoses | 1.706877 | 0.1235099 | 7.39 | 0 | 1.481184 | 1.966959 |
| Depression | 0.9029831 | 0.0302098 | -3.05 | 0.002 | 0.8456724 | 0.9641777 |
| Hypertension Uncomplicated | 0.7011589 | 0.0204987 | -12.14 | 0 | 0.6621115 | 0.7425091 |
| Hypertension Complicated | 0.1804289 | 0.0090025 | -34.32 | 0 | 0.1636197 | 0.1989649 |
| Coronary Artery Disease | 0.1591498 | 0.0065528 | -44.64 | 0 | 0.1468112 | 0.1725255 |
| Aspirin | 0.1285245 | 0.0064073 | -41.15 | 0 | 0.1165605 | 0.1417165 |
| Long Term Anticoagulation | 1.580254 | 0.0771721 | 9.37 | 0 | 1.436012 | 1.738983 |
| Long Term NSAID | 0.2814453 | 0.0201059 | -17.75 | 0 | 0.2446728 | 0.3237444 |
| Long Term Steroid | 0.0973572 | 0.0238198 | -9.52 | 0 | 0.0602712 | 0.1572628 |
| Vitamin D Deficiency | 1.616639 | 0.1070108 | 7.26 | 0 | 1.419937 | 1.840589 |
| History of Bariatric Surgery | 0.0087535 | 0.0065903 | -6.29 | 0 | 0.0020014 | 0.0382854 |
| Acute Kidney Injury (AKI) | 7.023624 | 0.1967026 | 69.6 | 0 | 6.648484 | 7.419931 |
| Vasopressor Use | 45.31263 | 2.01161 | 85.9 | 0 | 41.53661 | 49.43193 |
| Remdesivir Administration | 2.138419 | 0.0595806 | 27.28 | 0 | 2.024774 | 2.258442 |
| _cons | 0.0146406 | 0.0042842 | -14.43 | 0 | 0.0082505 | 0.02598 |
| Note: P>\|t\|=0: p≤0.001 | | | | | | |

| **Hospital Division**  **Frequency (N, (weighted)** | **ACU-Dep** | **No ACU-Dep** |
| --- | --- | --- |
| **New England** |  |  |
| **Frequency: 41,055** | 9.29% | 90.71% |
|  |  |  |
| **Mid-Atlantic** |  |  |
| **Frequency: 121,920** | 13.74% | 86.26% |
|  |  |  |
| **East North Central** |  |  |
| **Frequency: 143,895** | 8.14% | 91.86% |
|  |  |  |
| **West North Central** |  |  |
| **Frequency: 76,740** | 13.00% | 87% |
|  |  |  |
| **South Atlantic** |  |  |
| **Frequency: 199,530** | 9.51% | 90.49% |
|  |  |  |
| **East South Central** |  |  |
| **Frequency: 58,830** | 7.54% | 92.46% |
|  |  |  |
| **West South Central** |  |  |
| **Frequency: 86,515** | 7.06% | 92.94% |
|  |  |  |
| **Mountain** |  |  |
| **Frequency: 64,575** | 8.04% | 91.96% |
|  |  |  |
| **Pacific** |  |  |
| **Frequency: 121,110** | 8.39% | 91.61% |

**Table 16 Pearson Chi-square: Cannabis Dependence among 2020 Cannabis Use Encounters**

Pearson chi2(8) =1.0e+03 P-value: <0.001

**Legend:**Division 1 (New England): Maine, New Hampshire, Vermont, Massachusetts, Rhode Island, Connecticut

Division 2 (Mid-Atlantic): New York, Pennsylvania, New Jersey

Division 3 (East North Central): Wisconsin, Michigan, Illinois, Indiana, Ohio

Division 4 (West North Central): Missouri, North Dakota, South Dakota, Nebraska, Kansas, Minnesota, Iowa

Division 5 (South Atlantic): Delaware, Maryland, District of Columbia, Virginia, West Virginia, North Carolina, South Carolina, Georgia, Florida

Division 6 (East South Central): Kentucky, Tennessee, Mississippi, Alabama

Division 7 (West South Central): Oklahoma, Texas, Arkansas, Louisiana

Division 8 (Mountain): Idaho, Montana, Wyoming, Nevada, Utah, Colorado, Arizona, New Mexico

Division 9 (Pacific): Alaska, Washington, Oregon, California, Hawaii

**Supplementary Figure 4: Covid-19 Cannabis Use-related Discharges over Time by Hospital Division Legend:** Cannabis-related discharges for all cannabis-related users (cannabis use or cannabis dependence with or without remission) over time among the Covid-19 population.

Division 1 (New England): Maine, New Hampshire, Vermont, Massachusetts, Rhode Island, Connecticut

Division 2 (Mid-Atlantic): New York, Pennsylvania, New Jersey

Division 3 (East North Central): Wisconsin, Michigan, Illinois, Indiana, Ohio

Division 4 (West North Central): Missouri, North Dakota, South Dakota, Nebraska, Kansas, Minnesota, Iowa

Division 5 (South Atlantic): Delaware, Maryland, District of Columbia, Virginia, West Virginia, North Carolina, South Carolina, Georgia, Florida

Division 6 (East South Central): Kentucky, Tennessee, Mississippi, Alabama

Division 7 (West South Central): Oklahoma, Texas, Arkansas, Louisiana

Division 8 (Mountain): Idaho, Montana, Wyoming, Nevada, Utah, Colorado, Arizona, New Mexico

Division 9 (Pacific): Alaska, Washington, Oregon, California, Hawaii

**Supplementary Figure 5: Covid-19 Active Cannabis Abuse and Active Cannabis Dependence Discharges over Time
**

**Legend:** Cannabis-related Discharges for ACU-Abs and ACU-Dep over time among the Covid-19 Population by hospital region.

**Supplementary Figure 6: Covid-19 Active Cannabis Abuse and Active Cannabis Dependence Discharges over Time Legend:** Cannabis-related Discharges for ACU-Abs and ACU-Dep over time among the Covid-19 Population by hospital division.

Division 1 (New England): Maine, New Hampshire, Vermont, Massachusetts, Rhode Island, Connecticut

Division 2 (Mid-Atlantic): New York, Pennsylvania, New Jersey

Division 3 (East North Central): Wisconsin, Michigan, Illinois, Indiana, Ohio

Division 4 (West North Central): Missouri, North Dakota, South Dakota, Nebraska, Kansas, Minnesota, Iowa

Division 5 (South Atlantic): Delaware, Maryland, District of Columbia, Virginia, West Virginia, North Carolina, South Carolina, Georgia, Florida

Division 6 (East South Central): Kentucky, Tennessee, Mississippi, Alabama

Division 7 (West South Central): Oklahoma, Texas, Arkansas, Louisiana

Division 8 (Mountain): Idaho, Montana, Wyoming, Nevada, Utah, Colorado, Arizona, New Mexico

Division 9 (Pacific): Alaska, Washington, Oregon, California, Hawaii

**Supplementary Figure 7: Covid-19 Cannabis Remission Discharges over Time

Legend:** Cannabis-related Discharges and trend of CU-Rem over time among the Covid-19 Population by hospital region.

**Supplementary Figure 8: Covid-19 Cannabis Dependence Discharges over Time**

**
Legend:** Cannabis-related Discharges for Cannabis Dependence and ACU-Dep over time among the Covid-19 Population
